# dCas9-SPO11-1 locally stimulates meiotic recombination in rice

**DOI:** 10.3389/fpls.2025.1580225

**Published:** 2025-05-01

**Authors:** Léo Herbert, Aurore Vernet, Julien Frouin, Anne Cécile Meunier, Jeremy Di Mattia, Minghui Wang, Gaganpreet K. Sidhu, Luc Mathis, Alain Nicolas, Emmanuel Guiderdoni, Ian Fayos

**Affiliations:** ^1^ Meiogenix SA, Paris, France; ^2^ Centre de coopération internationale en recherche agronomique pour le développement (CIRAD), Unité mixte de recherche - Amélioration génétique et adaptation des plantes méditerranéennes et tropicales (UMR AGAP) Institut, Montpellier, France; ^3^ Unité mixte de recherche - Amélioration génétique et adaptation des plantes méditerranéennes et tropicales (UMR AGAP) Institut, Université de Montpellier, Centre de coopération internationale en recherche agronomique pour le développement (CIRAD), Institut national de recherche pour l'agriculture, l'alimentation et l'environnement (INRAE), Institut Agro, Montpellier, France; ^4^ Ingénierie et Analyse en Génétique Environnementale (IAGE), Montpellier, France; ^5^ Meiogenix Inc., Center for Life Science Ventures Cornell University, Ithaca, NY, United States; ^6^ IRCAN (Institute for Research on Cancer and Aging), CNRS (Centre national de la recherche scientifique) UMR7284, INSERM (Institut national de la santé et de la recherche médicale) U1081, Université Côte d’Azur, Nice, France

**Keywords:** CRISPR/dCas9, meiosis, plant breeding, pollen typing, rice, Spo11, targeted recombination

## Abstract

**Introduction:**

Meiotic crossovers shuffle the genetic information transmitted by the gametes. However, the potential to recover all the combinations of the parental alleles remains limited in most organisms, including plants, by the occurrence of only few crossovers *per *chromosome and a prominent bias in their spatial distribution. Thus, novel methods for stimulating recombination frequencies and/or modifying their location are highly desired to accelerate plant breeding.

**Methods:**

Here, we investigate the use of a dCas9-SPO11-1 fusion and clusters of 11 gRNAs to alter meiotic recombination in two chromosomal regions of a rice hybrid (KalingaIII/Kitaake). To accurately genotype rare recombinants in regions of few kbp, we improved the digital PCR-based pollen-typing method in parallel.

**Results:**

Expression of the dCas9-SPO11-1 fusion protein under the ubiquitous *ZmUbi1 *promoter was obtained in leaves/anthers/meiocytes and found to complement the sterility of the *Osspo11-1 * mutant line. We observed a 3.27-fold increase over wild-type (p<0.001) of recombinant pollens in a transgenic hybrid line (7a) targeting a chromosome 7 region. In the offspring plant 7a1, a significant 2.05-fold increase (p=0.048) was observed in the central interval (7.2 kb) of the Chr. 7 target region. This stimulation of meiotic recombination is consistent with the expression of the dCas9-SPO11-1 fusion and gRNAs as well as with the ChIP-revealed binding of dCas9-SPO11-1 to the targeted region. In contrast, no stimulation was observed in other transgenic lines deficient in the above pre-requisite features, expressing the dCas9-SPO11-1 fusion but no gRNAs or targeting a Chr.9 region.

**Discussion:**

These results open new avenues to locally stimulate meiotic recombination in crop genomes and paves the way for a future implementation in plant breeding programs.

## 1 Introduction

Global climate change in the context of continuous growth of the human population challenges the agriculture sustainability. This raises the need to develop novel methods to create new varieties of crops with improved agronomic traits such as higher yield, water and fertilizer use efficiencies, abiotic stress tolerances, pest and disease resistances as well as nutritional qualities (Atlin et al., 2017). To make it feasible, one can explore the extensive repertoire of natural polymorphisms and phenotypic variations existing in genetic resources, create new allelic series through conventional mutagenesis or gene editing while improving the molecular strategies to introduce the superior alleles in elite lines (Scheben and Edwards, 2018).

Meiosis is a unique and evolutionary conserved cell differentiation process shared by all eukaryotes, with the key function to halve the parental genome transmitted by the paternal and maternal gametes. It depends on the formation of bivalent structures that connect the homologous chromosomes, held together by sister chromatid cohesion and crossovers. Thus, meiotic recombination has the dual role to generate the genetic diversity transmitted by the gametes and ensure the reductional segregation of the homologs (Mercier et al., 2015). Independently of the physical length of the genomes, there are only 1 or 2 crossing-over (CO) *per* chromosome *per* generation in plants (Mercier et al., 2015). The additional limitation are the strong biases of CO distribution along the chromosomes, creating cold if not refractory recombination domains. In rice, bread wheat, barley, maize and other crops, COs occur in the euchromatic region of the chromosomes and more predominantly towards their distal parts (Lambing et al., 2017). Most dramatically, COs are scarce in large heterochromatic and peri-centromere regions that can represent more than 80% of the physical chromosome length (Darrier et al., 2017). Consequently, a large number of alleles/genes remain in their parental configuration over multiple generations. This constrains the recovery of the full breadth of novel allele combinations and prevents to resolve linkage drags when beneficial and detrimental alleles happen to be genetically linked on the same chromosome. Consequently, a large number of alleles/genes remain in their parental configuration over multiple generations, constraining the recovery of the full breadth of novel allele combinations. Also, tight associations between beneficial and detrimental alleles at genetically linked loci, the so called linkage drag in breeding, cannot be broken (e.g (Vikram et al., 2015; Voss-Fels et al., 2017)). Thus, biotechnological innovations to engineer the CO landscapes are highly desirable.

Meiotic recombination is initiated by hundreds of programmed DNA double strand breaks (DSB) catalyzed by the evolutionary conserved Spo11 transesterase and associated core complex proteins (Bergerat et al., 1997; Keeney et al., 1997; Yadav and Claeys Bouuaert, 2021). Spo11 is a ortholog of the archaeal topoisomerase VI catalytic A subunit that acts with the non-catalytic B subunit encoded by the Rec102/Rec104 proteins in *S. cerevisiae* (Claeys Bouuaert et al., 2021b) or MTOPVIB in plants (Vrielynck et al., 2016, Vrielynck et al., 2021). While in many species, a single *SPO11* gene is present (e.g., fungi, invertebrates and mammals), the plant genomes encode several non-redundant SPO11-related genes (Stacey et al., 2006; Hartung et al., 2007; An et al., 2011; Da Ines et al., 2020). Amino acid sequence comparison and mutants in *Arabidopsis*, rice, maize and wheat demonstrated that SPO11-1 and SPO11-2 are required for meiotic recombination and their individual inactivation leads to severely reduced fertility or complete sterility (Grelon, 2001; Stacey et al., 2006; Hartung et al., 2007; Yu et al., 2010; Fayos et al., 2020; Benyahya et al., 2020; Ku et al., 2020; Li et al., 2022; Hyde et al., 2023). Biochemically, DSB formation in plants is likely catalyzed by a hetero-tetramer protein complex composed of a SPO11-1/SPO11-2 heterodimer and a homodimer of MTOPVIB (Vrielynck et al., 2016). Additional evolutionary conserved proteins are also essential to achieve/modulate DSB formation (Claeys Bouuaert et al., 2021b; Yadav and Claeys Bouuaert, 2021). Their functions relate to a large panel of biological processes, comprising: (i) replication that occurs before DSB formation (Smith et al., 2001; Murakami et al., 2003; Murakami and Keeney, 2014), (ii) chromosome axis structure where DSBs occur (Klein et al., 1999; Zickler and Kleckner, 1999), (iii) accessory factors of the core Spo11 complex (Sasanuma et al., 2007; Claeys Bouuaert et al., 2021b, Claeys Bouuaert et al., 2021a), (iv) DSB recognition ability of ligation enzymes involved in NHEJ (Lascarez-Lagunas et al., 2023), (v) fine scale and genome wide chromatin features that dictate the DNA accessibility (Ohta et al., 1994; Wu and Lichten, 1994; Lascarez-Lagunas et al., 2023), (vi) histone modifications (Petes, 2001; Sollier et al., 2004; Borde et al., 2009; Acquaviva et al., 2013; Choi et al., 2013; Lascarez-Lagunas et al., 2023) or (vii) DNA methylation (Choi et al., 2013, Choi et al., 2018). Environmental cues, such as temperature, also moderately modulate meiotic recombination frequencies (Modliszewski and Copenhaver, 2017).

Once DSBs are formed, numerous proteins control their repair including the key steps of DSB end resection, choice and strand invasion of the homologous template and subsequent maturation of the DNA intermediates that ultimately lead to the production of non-crossovers (NCO) or crossovers (CO) recombinant chromosomes (Mercier et al., 2015). In rice male meiocytes, only approximately 5-10% DSBs mature into COs (Wang and Copenhaver, 2018) and cytological analyses of the DMC1/gammaH2AX foci estimated that 200-300 DSBs are formed *per* meiosis, leading to an average of 17 COs genome-wide (Si et al., 2015; Zhang et al., 2020). The number and the position of the recombination events *per* chromosome are also tightly regulated by the phenomena of CO designation, homeostasis and interference that ensure an obligatory CO *per* chromosome and control their spatial distribution (Martini et al., 2006; Muyt et al., 2008; Ziolkowski and Henderson, 2017; Girard et al., 2023).

Nevertheless, although homologous recombination is obligatory, the variable location of the recombination events from one meiosis to another, the variation of the genetic map between closely related species and the result of genetically engineered cells revealed flexibility (Székvölgyi and Nicolas, 2010). Operationally, the large diversity of factors modulating meiotic recombination also raised the perspective that biotechnological methods could be developed to modify the meiotic recombination landscape. In *Arabidopsis* and few other plants (rice, tomato and pea), a genome-wide stimulation of meiotic recombination at natural sites has been achieved through the inactivation of genes encoding the synaptonemal complex ZYP1 (Wang et al., 2015; Capilla-Pérez et al., 2021; Liu et al., 2021) and SCEP1 and SCEP2 (Vrielynck et al., 2023) proteins, and anti-CO proteins (AAA-ATPase FIGL1 (Girard et al., 2015) and DNA Helicases RECQ4 and FANCM (Fernandes et al., 2018; Mieulet et al., 2018)) as well as the ectopic expression of the pro-CO gene HEI10 (Ziolkowski et al., 2017). However, whereas global modulation of meiotic recombination is desirable for harnessing wild and cultivated diversity in a pre-breeding approach, prediction models suggest that programming recombination-assisted breeding with induction of targeted COs at only few specific chromosomal sites is preferred to improve elite germplasm (Taagen et al., 2020).

Modeling studies in barley, maize and rice indicated that upon targeted recombination, genetic gains could be increased for polygenic traits such as yield, as much as 15-28% and improve introgression outcomes (Brandariz and Bernardo, 2019; Ru and Bernardo, 2019; Epstein et al., 2023). Along this line, a method of choice in plants would be to implement the targeting methods developed in budding yeast that are based on the fusion of Spo11 to a sequence-specific DNA-binding module (Peciña et al., 2002). Targeting specific genomic sites with various transcription factor, Zn-finger, TALE and dCas9 DNA binding modules enabled to locally increase meiotic DSBs formation, up to 50-fold and recombination (CO and NCO) up to 6.3-fold (Peciña et al., 2002; Murakami and Nicolas, 2009; Sarno et al., 2017). However, DSB formation was not stimulated at all targeted sites, indicating that some chromosomal regions remained refractory to DSB formation (Robine et al., 2007; Sarno et al., 2017). Targeted DSB stimulation was also achieved upon fusion of the Gal4 binding domain to others proteins controlling DSBs formation (Koehn et al., 2009; Acquaviva et al., 2013). However, targeting recombination at the 3a crossover hot spot in an *Arabidopsis thaliana* hybrid through expression of a MTOPVIB-dCas9 fusion and 6 gRNAs showed no increase in meiotic recombination (Yelina et al., 2022). In contrast, we now report the use of a dCas9-SPO11-1 fusion protein able to locally stimulate meiotic recombination in a transgenic *indica/japonica* rice background.

## 2 Materials and methods

### 2.1 Plasmid constructs and transformation

A nucleolytically inactive version of a rice codon-optimized *Streptococcus pyogenes Cas9* sequence (Miao et al., 2013) (*dCas9*) was designed by introducing the D10A and H840A amino acid changes at the HNH and RuvC nucleolytic sites according to (Jinek et al., 2012). A V5 tag (MGKPIPNPLLGLDST) followed by the nuclear localization signal NLS (APKKKRKV) was added at the dCas9 N-terminus through a GIHGVPAA linker (Cong et al.,2013). The synthesized sequence of the *dCas9* fragment spanned 4151 base pairs from the ATG codon and terminated by an Stu1 site to allow in frame fusion to another coding sequence (here the genomic coding sequence of OsSPO11-1 LOC_Os03g54091). Eventually a *Kpn*I restriction site was added at the 5’ end of the sequence upstream the V5 tag and a *Sac*I restriction site was added at the 3’ end of the sequence downstream the *Stu*I site. The resulting final sequence was inserted in a pCAMBIA5300 pZmUbi1-MCS (multiple cloning sites)-tNOS binary vector. Three sequences starting with an *Stu*I site and spanning the last 47 nucleotides of *Cas9* sequence followed by a stop codon (dCas9-empty) or a PEFMAMEAPGIR linker (Sarno et al., 2017) and the genomic sequence of the *OsSPO11-1* (g or cDNA) in which the *Stu*I sites were previously altered using silent mutations (dCas9-SPO11-1) were assembled. A second *Stu*I restriction site was added at the 3’ end of the *dCas9-SPO11-1* cassette. These sequences were synthesized into a pUC57 vector. We then isolated and inserted the StuI-end-Cas9-Stop-StuI or StuI-end-Cas9-gDNA-SPO11-1-StuI fragments into the pZmUbi1-dCas9-StuI-tNos binary vector. All syntheses were subcontracted to Genscript (Germany).

The gRNA expression cassettes (11 and 6 gRNAs) based on tRNA:gRNA processing system (Xie et al., 2015) were synthesized by Genscript and inserted into a pCambia2300 using *Fok*I and *Bsa*I restriction sites. All gRNA have been designed using CRISPOR (Concordet and Haeussler, 2018) (**Supplementary Table S4**) and target both Kitaake and KalingaIII alleles. To minimize off-target effects we choose gRNA sequences exhibiting at least 4 mismatches with the closest potential off -targets sequences. Moreover, we pay attention that these mismatches are located as close as possible from the PAM of the recognition site.

The resulting plasmids (**Supplementary Figure S8**) were introduced into *Agrobacterium tumefaciens* strain EHA105 by electroporation. Cell suspensions of the two strains, harboring the *dCas9-OsSPO11-1* T-DNA and the tRNA:gRNA T-DNA, were mixed at equal volume before co-culture with rice seed embryo-derived calluses. Selection of transformed cell lines based on both hygromycin (50mg/l) and geneticin (200mg/l) resistances is a derivative of the protocol from (Sallaud et al., 2003). The dCas9-empty T-DNA was transformed alone in the Kitaake genotype. To assay the tRNA:gRNA system, we used the 6 gRNAs T-DNA and the active *Cas9* plasmid from (Miao et al., 2013) optimized in (Fayos et al., 2020).

### 2.2 Plant materials and growth conditions

All the genes used as reference or present in the two targeted regions are given in the article for the Kitaake genome. MSU and RAPdb correspondences are provided in **Supplementary Table S10**.

Manually produced F1 seeds of the cross between the *indica* cultivar KalingaIII and the *japonica* cultivar Kitaake and their parental control seeds are used in this study. A F2 population of ca. 2,000 progeny seedlings was derived by selfing KalingaIII/Kitaake F1 plants for DNA isolation and KASP genotyping. For pollen typing assays, the KalingaIII/Kitaake F1 hybrid control plants used for pollen collection were grown along the transgenic KalingaIII/Kitaake F1 plants under the same greenhouse conditions. Genomic DNA of a 379 plants sub-population of this large KalingaIII/Kitaake F2 progeny was used for whole genome sequencing at an average 3-4x depth at the Genoscope, Evry, France (K. Labadie). Following genotype calling, accurate breakpoint positions were determined using the NOISYmputer improved software (Triay et al., 2025) and a linkage map established. Genomic DNAs of 20 plants among the 379 F2 plant population were also further sequenced at a 20x depth to confirm the accuracy of the breakpoint position in the low coverage assemblies (Fayos, Petit et al., in preparation).

The *spo11-1* mutant used for complementation corresponds to the *spo11-1-1* allele published by (Fayos et al., 2020) (insertion of an A nucleotide in the ATG). As the mutant is propagated via the heterozygous genotype, we amplified and sequenced the mutated region using *SPO11-1* primers (**Supplementary Table 5**) to verify that the mutation was homozygous in the complemented plants. Sequencing was performed using the *SPO11-1* F1 primer.

All rice plants were grown under greenhouse conditions as follow 28°C day/24°C night, 60% hygrometry. The natural light was completed by artificial LED light (700µmol/m^2^/s).

### 2.3 Nucleus extraction

Mature pollen was collected from dehiscent anthers and stored at -20°C. Nucleus extraction protocol was adapted from (Ahn et al., 2021). Extraction protocol and sorting example is detailed in **Supplementary Figure S5**.

### 2.4 Fluorescence-activated cell sorting

Cell sorting was performed on a FacsAriaIIu cell sorter (BD Biosciences) running on FACSDiva Version 9.0.1. PI was excited with a 488nm blue laser of 100mWatt and signal emission was captured with a 600 long pass filter and a 610/20 Band Pass filter. A Nozzle size of 70 µm, a frequency of 87 kHertz and a pressure of 70 PSI were used. The sheath fluid was 1x PBS. The threshold was set at 800 on the PI channel (No threshold on FSC). Settings of the PMT voltages are 250 for forward scatter (FSC), 288 for side scatter (SSC) and 280 for IP. All the parameters were read on a log scale. The sorting was performed using the purity mode. Regularity of sample flow was monitored by looking at the PI fluorescence signal as a function of time. Nuclei were first selected on a P1 gate using an SSC-A vs PI-A plot, and the sorting was done on a second Count *vs* PI-A plot by a Gate P2 that refines P1 nuclei gate. The nuclei were sorted directly into the dPCR mix. Prior to sorting the dPCR mix volume is set according to the amount of sorted nucleus preparation.

### 2.5 dPCR processing and analysis

dPCR experiments were conducted by I.A.G.E Company, Montpellier, France. Each experiment was carried out according to the experimental design. The final 40µL dPCR mix contains *Taq*1 enzyme to improve DNA accessibility during PCR (Taq1 V2 NEB). All 24 samples were loaded and partitioned in 26.000 PCR events in the 24 wells nanoplate. All partitions have the same volume: 0.91nl. dPCR probes design and optimizations were done in collaboration with I.A.G.E. Data were analyzed and plotted by I.A.G.E company with the QIAcuity Software Suite. All raw data tables are available in the **Supplementary Tables S2**, **S3**.

To assess the number of genotyped nuclei and recombination rates, we selected partitions with at least two fluorescences. The recombination rate *per* genotype has been calculated (Number of recombinant/Total number of nuclei) . To perform the correction, the background percentage was multiplied by the number of WT or transgenic nuclei to estimate the number of false-positive nuclei to be deducted from these populations. The recombination rate obtained in background was also used to estimate the error rate for each probe. For plants 7a, 7b, 9a and 9b we used as control a mix of pollen collected from WT F1 plants grown under the same experimental conditions in the greenhouse. For F2 progeny plants 7a1 and 7a2, we used as controls the pollen collected on a single WT F1 plant grown under the same experimental conditions of each plant.

### 2.6 DNA, RNA extraction and genotyping

Genomic DNA and/or RNA were isolated from leaf or anther tissues of the T0 primary transformants or from T1 progeny plants using standard rice protocols. For meiotic tissues, we isolated meiocytes (using a stereomicroscope) by squeezing each 6 anthers from a single flower in 4 µL of PBS buffer (~60 anthers). All primers for genotyping are detailed in the **Supplementary Tables S5**, **S6**.

### 2.7 qPCR assay

gDNAs and cDNAs were diluted to a 5 ng/μL concentration. Then, 1 μL is mixed with 0.3 μL of each of the forward and reverse primers (10 μM), 3 μL of SYBRgreen and 1.4 μL H_2_O. Reaction and real-time fluorescence reading were performed using a LightCycler 480 (Roche) with the following PCR conditions: 5 min at 95°C; 45 cycles (20s 95°C, 15s 60°C, 20s 72°C); and 5s at 95°C. A melting curve was then performed with 1 min at 65°C and a continuous increase of 0.11°C/s up to 95°C to ensure amplification of a single DNA. qPCR duplicate or triplicate were done for each sample.

For cDNAs, the expression rate was calculated as follows from the average CP for each triplicate: $\left(2^{-\left(Cp\;target\;gene-Cp\;EXP\right)}x100\right)$ . All primers and control are available in **Supplementary Table S6**. gDNAs were used to estimate the number of inserted T-DNA. Using DNA from a reference plant containing one copy of the hygromycin gene (verified by southern blot) the number of T-DNA copies was evaluated after normalization of the DNA amount.

### 2.8 Kasp assay

Single-nucleotide polymorphism (SNP) genotyping was performed using Kompetitive Allele Specific PCR (KASP) following the LGC group recommendations for the use of KASP technology on a Roche LightCycler 480 (**Supplementary Table S7**).

### 2.9 Chromatin immuno-precipitation

ChIP protocol was done as mainly described in (Bellegarde et al., 2018), and (Haring et al., 2007; Yamaguchi et al., 2014) using 500 mg of young leaves crosslinked with 1% formaldehyde and with slight modifications indicated hereafter: Nuclei were sonicated 10 cycles 30s ON 30s OFF in Bioruptor (Diagenode Pico) at 4°C. Incubation at 4°C overnight 2 µg of Anti-V5 Tag Antibody (R962-25 Invitrogen) *per* sample in 2mL of ChIP-Dilution-Buffer complemented with 10 µg/mL Bovine Serum Albumin. After purification with the iPURE kit V2 (Diagenode C030110014), DNA was analyzed with SYBR green qPCR, and each sample was normalized with his 10% adjusted to 100% INPUT (-3.32 Cp Input). V5-dCas9-SPO11-1 recovery relative to the input was calculated as follows: $\left(2^{-\left(Cp\;IP\;V5-Cp\;Input\;\right)}x100\right)$ . All primers and control gene (*OsKitaake06g078500.1*) (Caldana et al., 2007; Mounier et al., 2020) are available in **Supplementary Table S8**.

### 2.10 Western blot

Protein extraction was done in a buffer containing 10 ml Tris HCl pH8 1M, 2 ml MgCl_2_ 1M, 0.4 ml EDTA 500mM pH8, 10 ml glycerol 100%, 0.2 ml Triton X-100, 177.2 ml H_2_O. 10µL DTT was added to 10 µL of the protein extraction buffer. 200 mg of grinded leaf tissue or ~100 anthers (sized to select stages close to prophase) were vortexed in a tube with 100 to 800 uL of buffer then centrifuged 15min at 4°C to obtain the supernatant containing the proteins. Proteins quantification was carried out with Pierce Bovine Serum Albumin Standard Pre Diluted Set 23208 and Pierce Coomassie Plus Bradford Assay Reagent 23238 following the Fisher Scientific instructions. 40 µl of reaction containing Bolt LDS Sample Buffer (4X), Bolt Reducing Agent (10X) from Fisher scientific and 20 or 40 µg of proteins were heated at 75°C 10min to denature the proteins. Samples were loaded in a Bolt 4-12 Bis-Tris Plus Gels, 10-Well NW04120BOX from Fisher Scientific at 200V in 1X MES buffer. The proteins were then transferred to a nitrocellulose membrane in a Biorad mini trans blot cell overnight in CAPS buffer at pH11 at 30V at 4°C. Membrane and gel were stained with Ponceau or SimplyBlue SafeStain respectively to verify the proteins transfer. Finally, the proteins were tagged with V5 Tag Antibody, (R962-25 Invitrogen) and revealed with the WesternBreeze Chromogenic Kit, anti-mouse from Fisher Scientific.

## 3 Results

### 3.1 Generation of the dCas9-SPO11-1 + gRNAs transgenic hybrid plants

**Figure 1:**
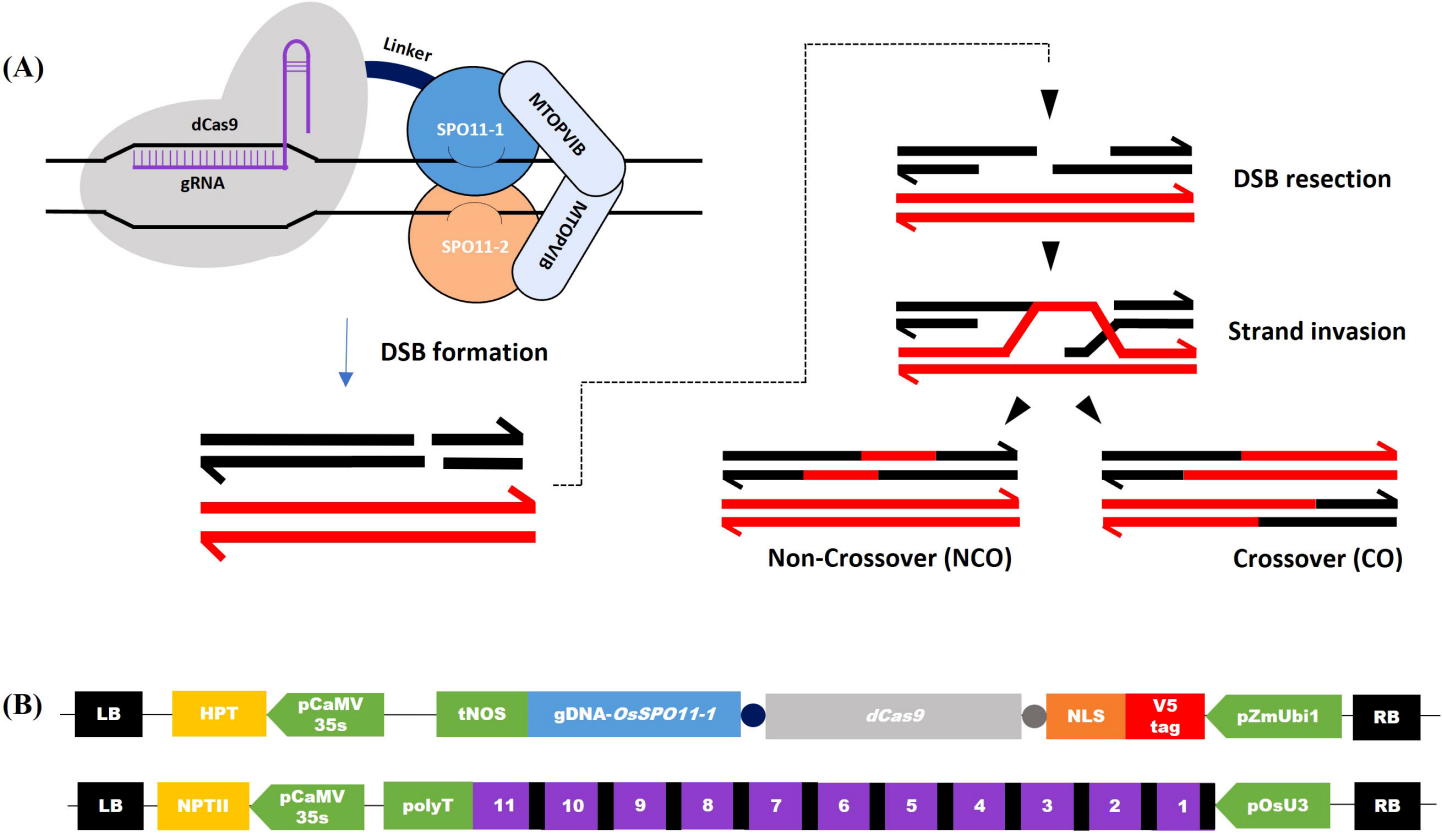
Induction of double-strand breaks by dCas9-SPO11-1 and gRNAs transgenes. **(A)** Schematic representation of hypothetical DNA repair following dCas9-SPO11-1-induced Double Strand Break (DSB) at prophase of meiosis. *Left:* dCas9 (gray), fused to the SPO11-1 subunit (light blue), binds the target site and recruits the other components of the transesterase Spo11 complex, including SPO11-2 (orange) and MTOPVIB (grey). *Right:* Recombinational repair of Spo11-dependent DSB in meiosis: Spo11-dependent DSB formation on one of the homologous chromosomes (dark) and ssDNA resection allow strand invasion of the homologous chromosome. Progression and resolution of the recombination intermediates yield recombined non-crossover (NCO) and crossover (CO) molecules adapted from Yelina et al. (2022). **(B)** Structure of the T-DNA constructs co-introduced in rice. *Up:* T-DNA for dCas9-OsSPO11-1 expression. *Down:* T-DNA for transcription of the multiplexed tRNA:gRNA scaffold, which once managed by the natural tRNA processing system is supposed to release 11 gRNAs targeting either Chr.7 or Chr.9 regions (gRNAs and tRNAs are shown as purple and black boxes, respectively).

In this study, we fused the OsSPO11-1 genomic DNA to the C-terminus of the catalytically dead *Streptococcus pyogenes* Cas9 (dCas9), carrying 2 amino acid substitutions (D10A and H840A) that abolish its endonuclease activity but preserves its ability to bind target DNA *via* gRNAs (Qi et al., 2013) (**Figures 1A, B**). To visualize dCas9-SPO11-1 expression, we added an N-terminal in-frame 15 amino acid V5-tag followed by a 8 amino acid nuclear localization signal and, to facilitate the independent folding of the dCas9 and SPO11-1 domains, we added a 8 amino acid-linker upstream of dCas9 as well as another 12 amino acid-linker sequence between the dCas9 and *OsSPO11-1* sequences. Next, we used the regulatory region of the ubiquitous maize *ZmUbi1* gene (Christensen et al., 1992) and the tNos transcriptional terminator to direct the expression of this fusion in rice (**Figure 1B**). A constitutive promoter rather than a meiosis-specific promoter was chosen because it facilitates the identification of high expressers among T0 plants through the monitoring of dCas9-SPO11-1 mRNA and protein accumulations in the more accessible somatic tissues, while allowing expression in germinal tissues (Cornejo et al., 1993). Finally, we inserted this 12.8 kbp expression cassette in the T-DNA region of a binary vector.

**Figure 2:**
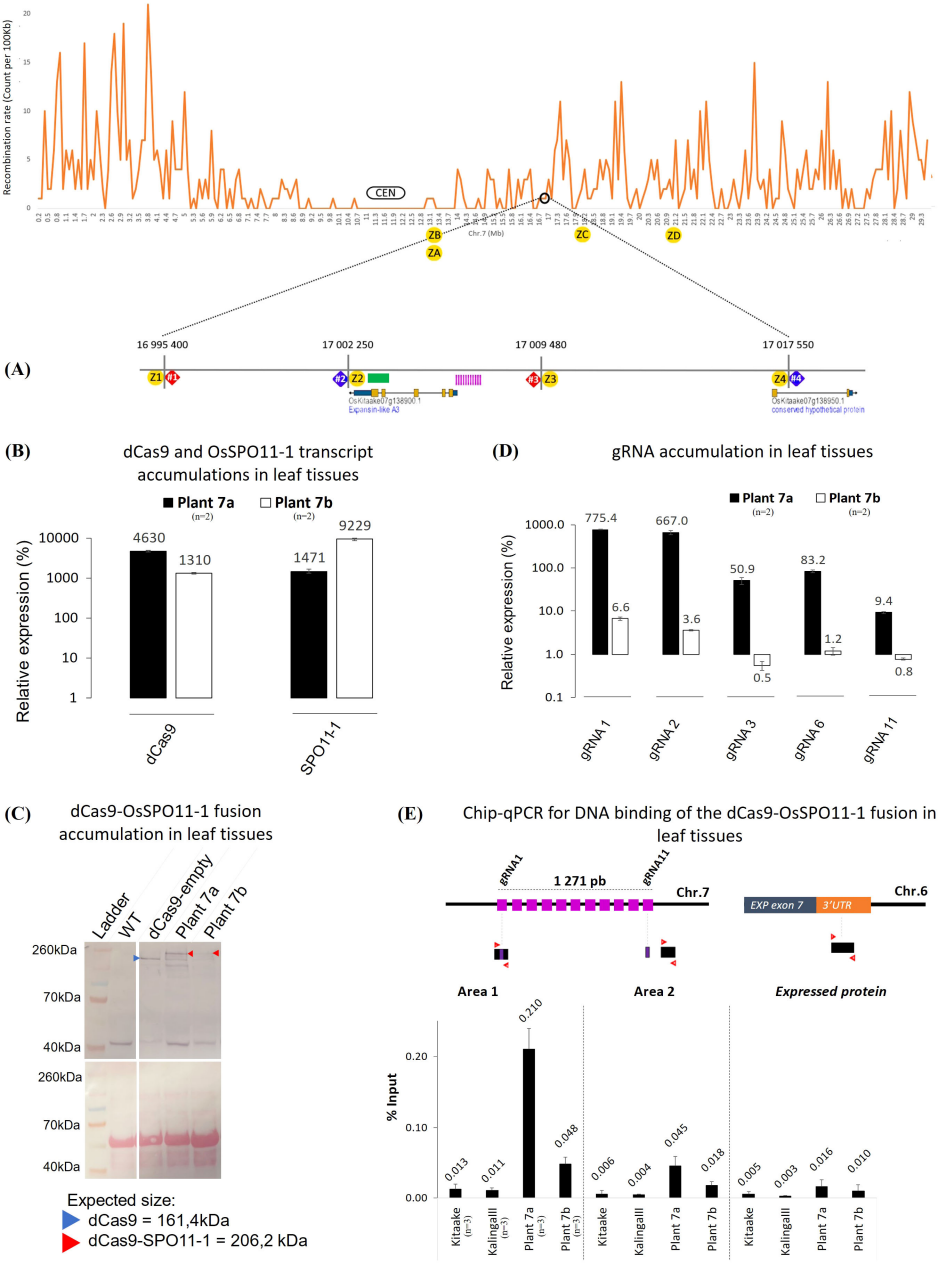
Molecular and functional characterization of the transgenic plants 7a and 7b. **(A)** Schematic representation of the recombination context of the targeted Chr.7 region established by whole genome sequencing of a 379 F2 progeny population of KalingaIII/Kitaake (Unpublished). The position of the dPCR (7#1, 7#2, 7#3 and 7#4) and Kasp (Z1 to Z4) markers with their coordinates on the Kitaake genome are detailed on the zoom. Kasp markers ZA to ZD were only used to genotype plant 7a offsprings (**Figure 5**). The green bar represents the area containing the recombinant identified in the progeny. The sequence of the polymorphic markers and 11 gRNAs (pink bars) are given in **Supplementary Tables S4**, **S9**. The 11 gRNAs target the 5’ promoter region of the *expansin-like A3* gene (*OsKitaake07g138900.1*). **(B)** RT-qPCR quantification of the dCas9 and SPO11-1 transcripts in leaf tissues of the transgenic KalingaIII/Kitaake plants 7a and 7b relative to *OsKitaake07g010600.1 Expressed protein* gene as reference (n=2). Values follow a Log10 scale. **(C)** dCas9-SPO11-1 accumulation in leaf tissues of plants 7a and 7b revealed by western blot analysis using an anti-V5 antibody. Expected gel migration positions of dCas9 (161 kDa) and dCas9-SPO11-1 (206 kDa) are pointed by blue and red arrowheads, respectively. **(D)** RT-qPCR accumulation of the gRNAs in leaf tissues of transgenic plants 7a and 7b. Values follow a Log10 scale and use the *OsKitaake07g010600.1* gene as a reference (n=2). **(E)** ChIP-qPCR of the targeted chromosome 7 region. Chromatin DNA of WT KalingaIII and Kitaake parents and independent transgenic KalingaIII/Kitaake hybrid plants 7a and 7b were immuno-precipitated using the anti-v5 antibody and quantified by qPCR. The sonicated fragments are in the range of 200-900 bp. Target sequences of the 11 gRNA scaffold are shown in purple. The qPCR amplified regions (area 1 and 2) are indicated by red arrowheads. The enrichment values are normalized by the input (10% of total chromatin). The 3’ region of the ubiquitously expressed gene *OsKitaake06g078500.1* residing on Chr.6 was used as a non-target control (n=3).

Concerning the gRNAs, we choose to target dCas9-SPO11-1 on Chr.7 and Chr.9 regions, where we had high quality information on the sequence of the KalingaIII and Kitaake genomes and local polymorphisms, as well as preliminary information on whole genome crossover profiles generated from 379 KalingaIII/Kitaake F2 progenies (unpublished). Both target regions are located in areas exhibiting moderate recombination rates, located 5Mb and 10Mb away from the centromeres of Chr.7 and Chr.9, respectively (**Figure 2A**; **Supplementary Figure S1A**). Practically, we built two T-DNA vectors each containing a multiplexed tRNA:gRNA scaffold designed to express 11 gRNAs (**Figure 1B**). Each T-DNA vector targeted a single chromosomal region, spanning 1,271bp and 1,622bp on Chr.7 and Chr.9, respectively. The rationale was to ensure that at least one well expressed gRNA would be efficient to bring the dCas9-SPO11-1 protein to the targeted region. Hopefully, if multiple gRNAs are expressed, the overall targeting efficiency could be boosted upon local diversification of chromosomal targets. To generate the transgenic lines, we co-introduced the dCas9-SPO11-1 T-DNA and one tRNA:gRNA T-DNA by the bacterial strain mix-method into F1 seed embryo derived calluses of the *indica/japonica* hybrid KalingaIII/Kitaake (**Figure 1B**). Co-transformed plantlets harboring both T-DNAs were raised on the basis of a dual resistance to hygromycin and geneticin and validated by the presence of the *hpt* and *npt* genes in several primary transformants (T0).

### 3.2 Expression of dCas9-SPO11-1 and gRNAs

To examine whether the dCas9-SPO11-1 transgene is transcribed, total RNA isolated from leaves of several primary transformants was assayed by RT-PCR. Transcript accumulation was variable across T0 plants. Four independent events that exhibited the highest RT-PCR signals were retained and hereafter referred as plants 7a, 7b and, 9a, 9b for those carrying the Chr.7 and Chr.9 gRNAs, respectively (**Figures 2B**, **Supplementary Figures S1B**, **S2**). Next, we performed western blot analyses on total protein extracts from leaf tissues, using a V5 antibody. Compared to the non-transformed (WT) and dCas9-empty plant extracts (Methods), a specific >200kDa band corresponding to the molecular weight of the dCas9-SPO11-1 fusion protein (206 kDa) clearly accumulated in the transgenic plant 7a while fainter signals were observed in the transgenic 7b (**Figure 2C**), 9a and 9b (**Supplementary Figure S1C**). In all the plants, signals of lower molecular weight were also observed. Among these, the occurrence of a band with a size similar to dCas9 raises the possibility that some free V5-NLS-dCas9 and SPO11-1 proteins are formed either upon abortive transcription or aberrant splicing. In rice, the existence of 6 *OsSPO11-1* alternate transcripts, generated by intron retention and/or altered 5’ and 3’ splice sites has been reported (Sprink and Hartung, 2014). In addition, *in vivo* and/or *in vitro* proteolysis may occur within or around the linker separating the dCas9 and SPO11-1 domains. Altogether, the above results demonstrated that the *ZmUbi1* promoter allowed the translational expression of the fusion protein *in planta* but at different levels from one transgenic plant to another.

Next, to investigate the individual expression of the gRNAs in leaves, we performed a gRNA-specific qPCR assay using the *OsKitaake07g010600* gene as a reference. The accumulation of the gRNAs expressed from the multiplexed tRNA:gRNA scaffolds was heterogenous. A strong qPCR signal was observed for 5 gRNAs (10 to 775-fold higher than the reference) in the transgenic plant 7a while a weaker signal (0.5 to 6.6-fold) was observed in the plant 7b for the same gRNAs (**Figure 2D**). In the 9a and 9b plants, transcription of the gRNA-11 was the highest (22 to 33-fold) while that of the gRNAs -4 and -5 reached only 5-fold (**Supplementary Figure S1D**). To note, the transcription of the gRNAs was not strictly correlated within their relative position in the scaffold. In conclusion, verification of the expression of both dCas9-SPO11-1 and gRNAs components designated the plant 7a as the most promising transgenic line to assay targeted meiotic recombination.

Differently, to test the functionality of the gRNAs *in planta*, we performed a live Cas9 mutagenesis assay. T-DNAs encoding a catalytically active Cas9 and a multiplex of 6 gRNAs targeting the Chr.7 region were introduced in seed-embryo derived calluses of cultivar Kitaake. Numerous putative transformant cell lines were selected on the antibiotic-supplemented medium. Then, PCR amplification of a 1,895 bp genomic region spanning the 6 gRNAs target sites was performed (**Supplementary Figure S3**). A range of Cas9-induced deletions was observed in several resistant cell lines (**Supplementary Figure S3**). Sanger sequencing confirmed the recovery of various deletions spanning the distal gRNA -1 to -6 target region and revealed additional frameshift mutations located in the vicinity of the gRNA targets. These functional results demonstrated the efficiency of some if not all gRNAs at guiding Cas9-mediated DSBs and confirmed the *in vivo* accessibility of the targeted Chr.7 region, at least in somatic cells.

### 3.3 Binding of dCas9-SPO11-1 to the target regions

To test whether the dCas9-OsSPO11-1 fusion binds the targeted regions, we performed a ChIP-qPCR assay using leaf tissues of the 7a, 7b and 9b lines, and an anti-V5 antibody targeting the N-terminal tag of the fusion protein. Relative to the input material, a significant qPCR signal >16 fold higher than those of the two parents (0.21 *vs*. 0.013 and 0.011% for the KalingaIII and Kitaake controls, respectively) was observed in the plant 7a (**Figure 2E**) and more strongly in the region targeted by the highly expressed gRNA-1, and -2 (**Figures 2D, E**). In the same region, a modest ChIP enrichment was observed in the 7b plant (**Figure 2E**) as well as in the targeted Chr.9 region in the 9b plant (**Supplementary Figure S1E**). Altogether, the above series of prerequisite assays converged to optimally retain 1 out of our 105 transformed hybrid seed embryo-derived callus, namely the plant 7a. The other transgenic lines (7b, 9a and 9b) exhibited less promising features but for the sake of comparison, targeted meiotic recombination was also analyzed.

### 3.4 Expression of the dCas-9-SPO11-1 in meiotic tissues and functional complementation of *Osspo11-1*

**Figure 3:**
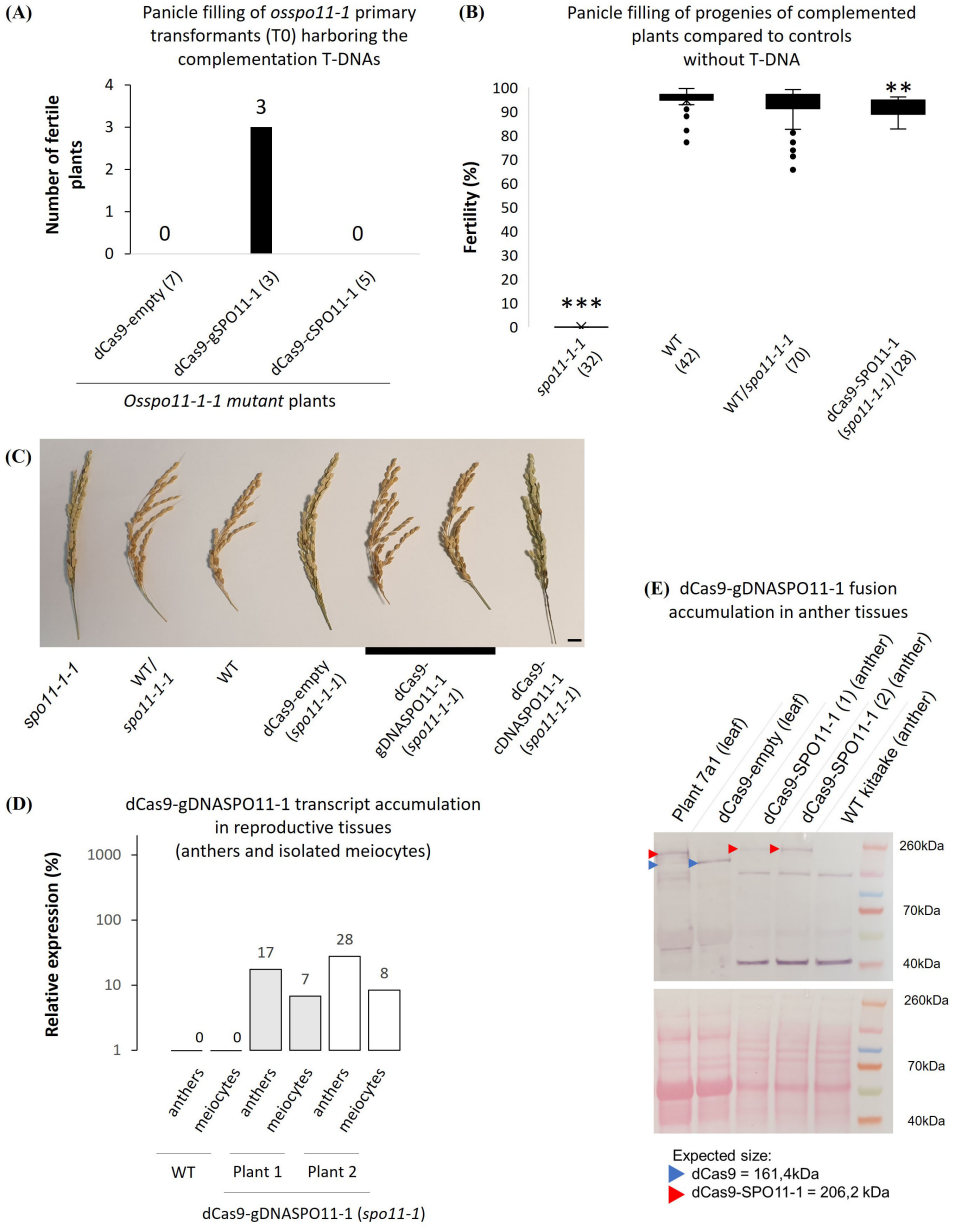
Meiotic expression and functional complementation of *Osspo11-1* by the dCas9-SPO11-1 fusion. **(A)** Recovery of fertile T0 plants regenerated from *Osspo11-/-* seed embryo-derived calluses harboring the dCas9-OsgSPO11-1 (genomic *SPO11-1* DNA) driven by the maize ubiquitin 1 (*pZmUbi1*) promoter region T-DNA (3 independent events) or a dCas9-empty, the T-DNA devoid of the g*SPO11-1* sequence (7 independent events) or the dCas9-cSPO11-1 (OsSPO11-1 cDNA) T-DNA (5 independent events). Only dCas9-OsgSPO11-1 T-DNA restores fertility in *Osspo11-1-1* -/- segregant progeny plants. **(B)** Fertility assessment of complemented *Osspo11-1-1* mutant lines. The *spo11-1-1*, WT and heterozygous WT/*spo11-1-1* genotypes contain both plants from pure lines and plants from complemented lines that have segregated the dCas9-empty, dCas9-gDNASPO11-1 or dCas9cDNASPO11-1 T-DNA. The dCas9-SPO11-1 individuals come from 2 independent fixed T3 lines and are all homozygous for the *spo11-1-1* mutation. Means are compared using a T.test between the WT and the other groups (*p* > 0.01**; *p* > 0.001***). **(C)** Photograph of WT, *spo11-1-1* mutant and complemented rice panicles used for fertility assessments in B (Scale bar=1cm). **(D)** RT-qPCR quantification of two dCas9-SPO11-1 *spo11-1* kitaake mutant plants transcripts in anthers and meiocytes tissues relative to *OsKitaake07g010600.1 Expressed protein* gene as reference. Amplifications were performed using primers located on the interval between the end of dCas9 and SPO11-1 including the linker. Values follow a Log10 scale. **(E)** dCas9-SPO11-1 accumulation in leaf tissues of plants 7a1 (used as positive control, see **Figure 6**), dCas9-empty plant and in anther tissues of two dCas9-gDNASPO11-1-complemented *spo11-1* plants revealed by western blot analysis using an anti-V5 antibody. Expected gel migration positions of dCas9 (161 kDa) and dCas9-SPO11-1 (206 kDa) are pointed by blue and red arrowheads, respectively. In contrast to the leaf tissues of plant 7a1, we only detect the dCas9-SPO11-1 protein in its integral form in anther tissues.

We next asked whether the dCas9-gOsSPO11-1 fusion construct accumulate at a sufficient level in meiotic tissues to restore the fertility of a fully sterile *Osspo11-1* mutant line. The *Osspo11-1* mutant meiotic chromosomes are deprived of meiotic DSBs, thereby lacking crossovers and undergo massive mis-segregations leading to complete sterility in rice (Yu et al., 2010; Fayos et al., 2020). Seed embryo-derived calluses of the *Osspo11-1-1* +/- heterozygous mutant line (Fayos et al., 2020) were transformed with either the pZmUbi1-dCas9-gOsSPO11-1 T-DNA construct or a negative control pZmUbi1-dCas9 T-DNA construct (lacking the in-frame gOsSPO11-1 sequence). Among the primary transformants harboring the pZmUbi1-dCas9-gOsSPO11-1 T-DNA, 3 independent events were found to derive from co-cultivated calluses having an *Osspo11-1* -/- homozygous mutant genotype (**Figure 3A**). The panicle filling of dCas9-SPO11-1 complemented plants (mean = 90.2%) was found to be slightly reduced compared to that of WT plants (t.test >0.01) (**Figures 3B,C**). They accumulate the fusion transcripts in leaves at a level comparable to those of plant 7a progenies used to monitor targeted recombination (**Supplementary Figure S4**). These plants which expressed the fusion transcripts in leaves, anthers and isolated meiocytes (**Supplementary Figures S4**, **S3D**), readily accumulated the 206 kDa dCas9-SPO11-1 fusion protein in anthers (**Figure 3E**), consistent with the restored fertility phenotype. Interestingly, contrasting with the pattern observed in leaves, a single protein product at the expected size was observed in anther extracts. It suggested that transcriptional and/or post translational behavior of the fusion is different in vegetative and reproductive tissues. In contrast, reminiscent to the untransformed *Osspo11-1-1* mutant line (Fayos et al., 2020), all 7 independent events harboring the dCas9 control T-DNA and derived from co-cultivated calluses having an *Osspo11-1* -/- homozygous mutant genotype were fully sterile. Mechanistically, alike in budding yeast (Sarno et al., 2017), this functional complementation demonstrates sufficient expression of the transgene to ultimately ensure at least one CO *per* pair of homologs. Besides the lack of a detectable degradation of the dCas9-SPO11-1 fusion protein in anthers (**Figure 3E**), it is not formally excluded that the observed restoration of fertility is mediated by a residual free form of SPO11-1 resulting from partial cleavage of the fusion between the Cas9 and SPO11-1 domains. Differently, in the wild type SPO11-1 background where the dCas9-SPO11-1 genome-wide activity would not be essential to ensure fertility, it is also possible that the fusion protein indirectly facilitate the recruitment of free SPO11-1 protein or Spo11 core complex proteins at a naturally cold targeted site, consistent with the observation that *in vitro* numerous Spo11 proteins and accessory factors accumulate on DNA (Claeys Bouuaert et al., 2021b).

### 3.5 Pollen typing strategy

**Figure 4:**
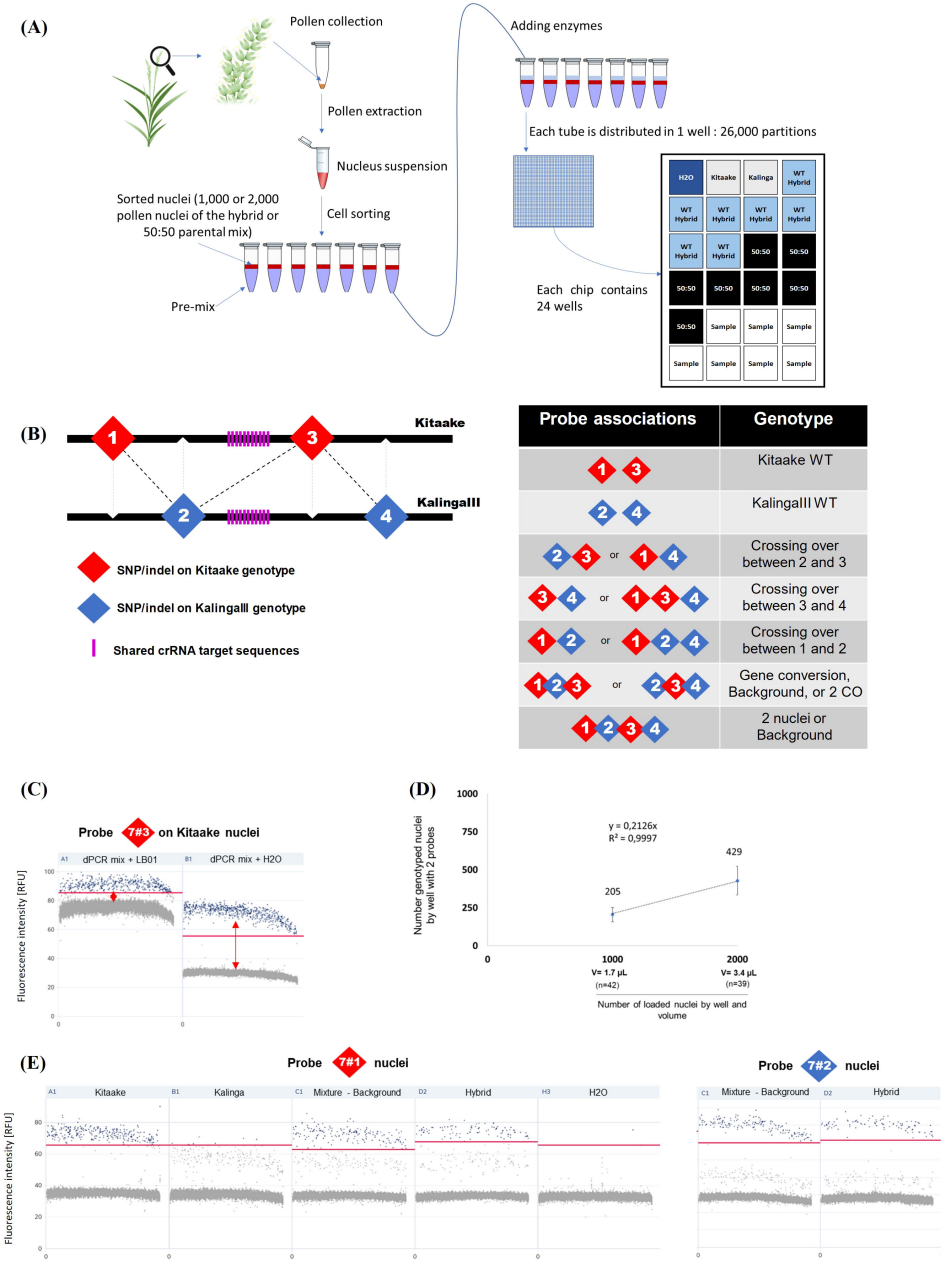
QIAcuity Digital PCR pollen typing strategy. **(A)** Experimental setup of the pollen typing experiment using dPCR. **(B)** Outline of the recombination assay. *Left:* The probes are distributed in a staggered pattern along the Kitaake and KalingaIII target chromosomal regions. *Right:* Probe associations are diagnostic of parental and recombinant alleles. **(C)** Optimization of the dPCR assay. After FACS sorting, a better cluster dissociation was observed between positive (blue) and negative (gray) partitions (threshold: red line) when nuclei are loaded in dPCR mix completed with H_2_O rather than with the LB01 buffer. **(D)** Fraction of genotyped nuclei (2 probes) *per* well when 1,000 or 2,000 nuclei are loaded. The volume of loaded nuclei through a 70 µm nozzle in the dPCR mix is 1.7µL and 3.4µL for 1,000 and 2,000 nuclei respectively. **(E)** Comparison of the specificities of chromosome 7 probes 7#1 and 7#2 specificity. Each sample of 1,000 nuclei is represented as a 1D Scatterplot for each probe and their fluorescence intensity (RFU). Each point represents a partition. A restrictive threshold (red line) separates gray (negative) and blue (positive) partitions. Each positive partition shows a fluorescence association that allows the nucleus genotype to be determined. Wells are also checked in 2D scatterplots (associations between 2 probes) to refine threshold positioning. Here no clear signal dissociation is observed for probe 7#1 between the KalingaIII and Kitaake alleles. The threshold (red line) signal dissociation is improved when 1,000 nuclei are loaded. In contrast, the signal of the probe 7#2 (as well as the probes 7#3 and 7#4, data not shown) clearly distinguishes the KalingaIII and Kitaake alleles. (additional information in Methods and **Supplementary Figure S6**).

The low frequency of meiotic recombination in most organisms, including rice (average of 4 cM/Mb) (Wu et al., 2003), limits the measure of meiotic recombination rates and requires to examine a large population of individuals in the progeny of hybrids. As an alternative, digital PCR has been developed to genotype a large number of DNA molecules from barley single pollen nuclei (Ahn et al., 2021). Here, to detect potentially rare (1/1,000) recombinant pollen at high resolution (Kbp) from KalingaIII/Kitaake hybrid, we used pollen nuclei sorting with FACSAria (**Supplementary Figure S5**) and a QIAcuity Digital PCR platform (Qiagen) that allows to use up to 5 fluorochromes. The overview of our variant pollen typing pipeline is outlined in **Figures 4A, B**, and the technical details described in the **Figure 4** legend and Methods section. Basically, for one experiment in one chip, we have 24 samples/tubes, and each tube is loaded into a well consisting of 26,000 independent partitions/reactions. In most instances, we used 3 control wells, 7 wells for the WT control population, 7 wells to evaluate the background noise (parental mix) and 7 wells for the transgenic plant. The number of experiments carried out and the total number of wells for each plant analyzed is given in **Table 1** and **Supplementary Table S1**. Our technical optimizations concerned the use of H_2_O rather than the standard LB01 buffer in dPCR (**Figure 3C, Methods**) and the calibration of the volume of sorted nuclei to distribute a maximum of 2,000 and sometime only 1,000 single pollen nuclei *per* well (**Figure 3D**, **Supplementary Figure S6**, Methods). These key adjustments led us to fully genotype about 20% of the sorted and loaded nuclei, and reach a robust genotyping sensitivity in the range of 10^-3^ recombinant nuclei.

**Table 1:** Recombination observed in the Chr.7 target region following genotyping of F2 progeny plants and of F2 or F3 pollen nuclei by KASP and/or dPCR.

	Interval	Method	Number of experiments	Total number of dPCR wells	Genotype	Total number of plants/nuclei	Number of recombinants	Corrected number of recombinants	Corrected recombination frequencies (%)	Fisher Exact Test
**Chr.7**	**2** 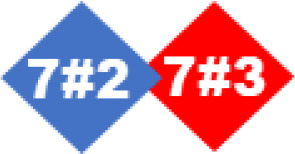	KASP			WT	1985	3		0,08	
		dPCR	6	41	Background	10256	10			
			6	42	WT	8834	18	9,4	0,11	0,027 *
			6	42	7a	6551	23	16,6	0,25	
			5	10	Background	2529	6			
			5	10	WT	2623	13	6,8	0,26	0,088
			5	85	7b	16266	59	20,4	0,13	
			8	55	Background	11523	8			
			8	56	WT	9403	19	12,5	0,13	0,048 *
			8	56	7a/1	7336	25	19,9	0,27	
			8	56	Background	9068	1			
			8	56	WT	8365	24	23,1	0,28	0,142
			8	56	7a/2	9220	16	15,0	0,16	
	**3** 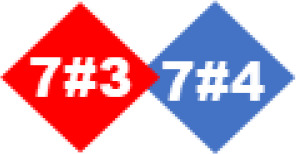	KASP			WT	1985	0		0,00	
		dPCR	6	41	Background	10256	6			
			6	42	WT	8834	6	0,8	0,01	0,006 **
			6	42	7a	6551	12	8,2	0,12	
			5	10	Background	2529	1			
			5	10	WT	2623	2	1,0	0,04	0,361
			5	85	7b	16266	8	1,6	0,01	
			8	55	Background	11523	2			
			8	56	WT	9403	6	4,4	0,05	0,394
			8	56	7a/1	7336	2	0,7	0,01	
			8	56	Background	9068	1			
			8	56	WT	8365	4	3,1	0,04	0,352
			8	56	7a/2	9220	2	1,0	0,01	

Estimation of the recombination frequency in the target regions was carried out by Kasp using a KalingaIII/Kitaake F2 progeny of ca. 2,000 individuals. Compared with pollen, the plants analyzed with Kasp are the product of two meioses. Kasp probes were designed using the same SNP/Indel as the dPCR probes or the closest functional SNP/Indel (**Figure 2A**). Genotyping of pollen nuclei was carried out according to the experimental flow shown in **Figures 4A, B**. Data from successfully genotyped pollen nuclei of biological replicates were pooled to assess the recombination frequency detected by pollen typing and corrected for technical backgrounds (Methods). The number of recombinants for interval 1 targeting Chr.7 could not be determined due to the lower specificity of probe 7#1 (**Figure 4E**, **Supplementary Figure S6** and **Supplementary Table S2**). However, by combining this probe with probe 7#3, we were able to estimate the number of nuclei genotyped for the Kitaake allele.

Colors yellow and green separate each plant analysis with the controls background and WT. Fisher exact test was performed between WT and transformed plants (p values are shown, p>0.05*; p>0.01**).

### 3.6 Meiotic recombination frequencies

**Figure 5:**
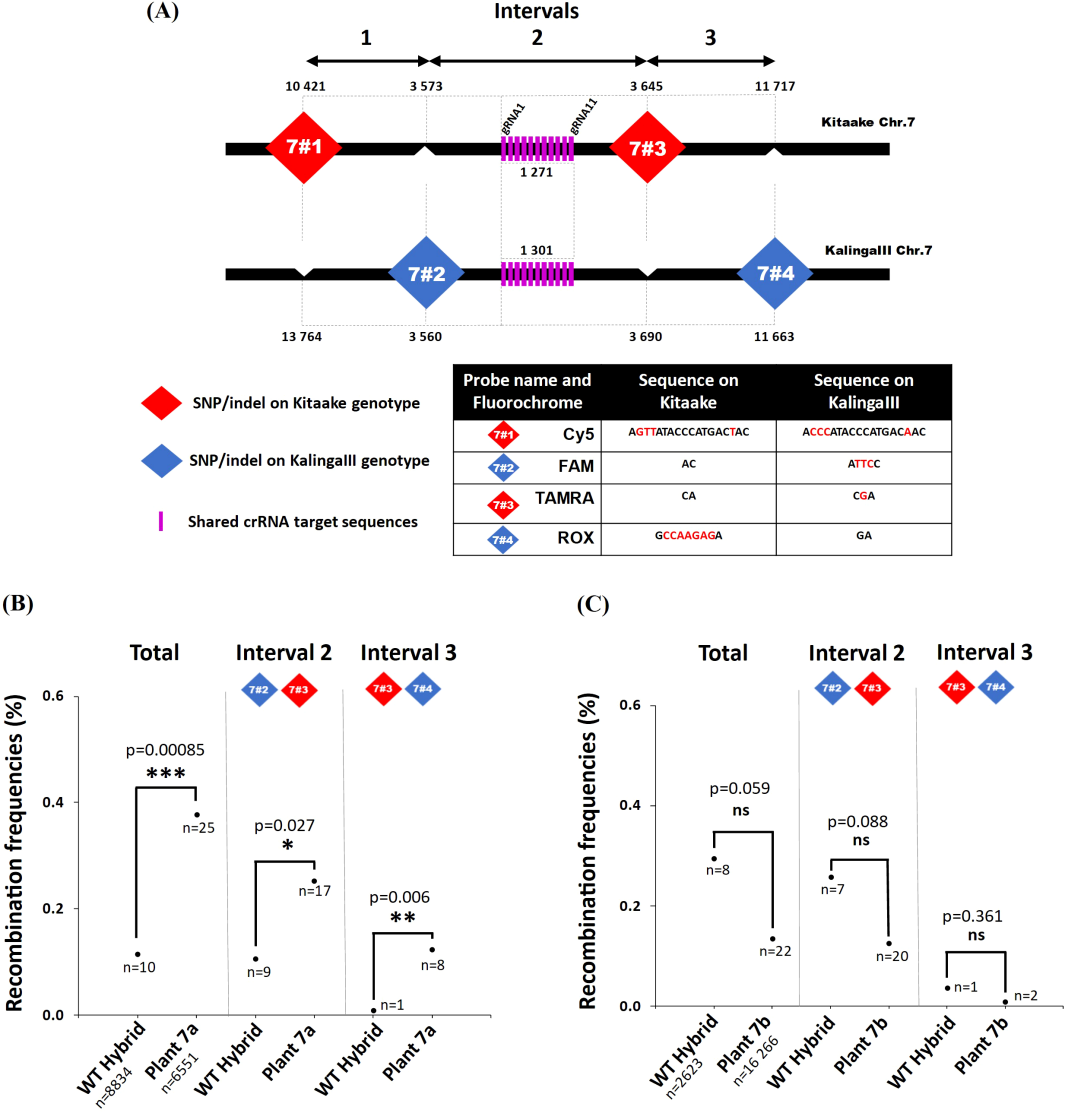
Recombination frequencies in the dCas9-SPO11-1 events and controls. **(A)** Positions of the KalingaIII and Kitaake markers flanking the targeted Chr.7 region. Probes are designed using the SNP/Indels shown in the table. Physical distances are given after the first gRNA position (17 005 820 on the Kitaake Chr.7). **(B, C)** Recombination frequencies over the target region of chromosome 7 deduced from genotyping nuclei WT and dCas9-SPO11-1 plants 7a and 7b pollen. Values are corrected by removing the background (Methods). Frequencies are compared over the whole region and then over each interval. The detailed data set is reported in **Table 1**. The corrected number of recombinant nuclei is indicated under recombination rate values. Fisher exact test was performed between WT and plants 7a and 7b (p values are shown, p>0.05*; p>0.01**; p>0.001***, ns, not significant).

To identify recombinant molecules using pollen nucleus typing, we designed 4 interspaced fluorescent probes that allow to genotype four KalingaIII/Kitaake polymorphic alleles (**Figure 4B**). On Chr.7, the distance between the outer flanking markers (markers 7#1 and 7#4) was ~22 Kbp and the inner interval (markers 7#2 and 7#3) spanned ~7,2 Kbp (**Figure 5A**). On Chr.9, the distance between the markers 9#1 and 9#4 was ~58 kbp and spans ~14,2 Kbp between the markers 9#2 and 9#3 (**Supplementary Figure S7A**). Our genotyping results demonstrated that 7 out of 8 tested probes robustly separate the KalingaIII and Kitaake allelic signals with an error rate of ~1/1,000 nuclei. A higher error rate of 2.8/100 molecules was observed for the less performant probe 7#1 (**Figure 4E**; **Supplementary Tables S2**, **S3**). As a consequence, the potential recombination events genotyped with marker 7#1 were not taken into account. Nevertheless, the genotyping accuracy of the Kitaake marker 7#1 plus 7#3 was sufficiently reliable to estimate the number of Kitaake parental nuclei in each experiment.

In our first experiments, 1,000 or 2,000 nuclei were partitioned *per* well for the analysis of 7a and 7b or 9a and 9b plants, respectively. As previously described, in most instances, for each experiment we used ~7 replicates/dPCR well for each of the 3 populations of nuclei (parental mix, wild type and transgenic plant). The set of pooled raw data is reported in the **Table 1**. To take into account the small number of fully genotyped nuclei (for at least 2 markers ~200 for 1,000 loaded nuclei) in each experiment and the scarcity of the recombinant molecules that limits the statistical power, we first analyzed the sum of the recombination events between the Chr.7 markers 7#2 to 7#4 (interval 2 and 3) and 9#1 to 9#4 (interval 1, 2 and 3) and hereafter each interval independently.

To determine the number of nuclei genotyped *per* experiment, we examined the parental combination of the markers 7#1 (Cy5 fluorochrome) and 7#3 (TAMRA fluorochrome) genotyping the parental Kitaake chromosome and the markers 7#2 (FAM fluorochrome) and 7#4 (ROX fluorochrome) genotyping the parental KalingaIII chromosome (**Figure 5A**). In all cases, the vast majority of the pollen nuclei (positive for 7#1 and 7#3 or 7#2 and 7#4) were of one or the other haplotype (mean=98.4%) and recovered in an almost similar 1:1 ratio (**Supplementary Tables S2**, **S3**). In the technical “background” pollen mix sample that was analyzed along with wild type and 7a plant samples, no recombinant pollen genotype is expected. However, out of 10,256 pollen nuclei, 16 (1.6 10^-3^) recombinant genotypes were detected between the markers 7#2 and 7#4. In the wild type plant, the frequency of recombinant genotypes was slightly higher but not significantly different from the background. Precisely, out of 8,834 pollen nuclei, 24 (2.7 10^-3^) recombinants were observed (**Table 1**). After subtraction of the background noise (Methods), the corrected recombination frequency between the markers 7#2 and 7#4 was 1.1 10^-3^ for the WT (**Table 1**). Importantly, upon Kasp genotyping of more than 2,000 KalingaIII/Kitaake offspring F2 plants in a segregating population, we found similar recombination rates (**Table 1**, **Supplementary Table S1**) demonstrating the accuracy of our pollen typing method. In rare instances (mean=2.1 10^-4^), we found a positive signal for all four markers in the same partition, likely resulting from the technical sorting of 2 pollens, one from each parental genotype. Alternatively, one cannot exclude that residual fragmented DNA are encapsulated within a single pollen well or that rare chromosomal non-disjunction events occurred before or during meiosis. Then, in the dCas9-SPO11-1 plant 7a, out of 6,551 single pollen nuclei, we detected 35 recombinant genotypes (5.3 10^-3^) down to 25 (3.8 10^-3^) after “background” subtraction (**Table 1**). Thus, before background correction, the meiotic recombination frequency in the plant 7a was 1.97-fold higher than in WT (2.7 10^-3^) (Fisher exact test p=0.012) and 3.27-fold higher than the WT (1.1 10^-3^) when background-corrected (p<0.001) (**Table 1**). When the interval 2 and 3 were independently analyzed, we also observed a significant background-corrected increases in plant 7a *vs.* WT control: from 1 10^-3^ to 2.5 10^-3^ (p=0.027) between the markers 7#2 and 7#3 and from 0.1 10^-3^ to 1.2 10^-3^ (p=0.006) between the markers 7#3 and 7#4 (**Table 1**, **Figure 5B**).

In contrast, in the typing experiment including plant 7b pollen, the background-corrected recombination frequencies between the same markers 7#2 and 7#4 (1.35 10^-3^ based on 16,266 nuclei) showed no increase in recombination with regards to those in the WT (2.95 10^-3^ based on 2,623 nuclei) (**Table 1**, **Figure 5C**). Finally, regarding the Chr.9 target region, we used four fluorochromes to genotype four interspaced markers 9#1 to 9#4 (**Supplementary Figure S7**) in pollen nuclei of two distinct transgenic hybrid plants (9a and 9b). In this region, the background frequencies estimated from the parental pollen mix preparation were of 4.1 and 3.9 10^-3^ nuclei, respectively (**Supplementary Table S1**). In the WT nuclei genotyped along plant 9a and 9b nuclei, the background-corrected recombination frequencies were 5.2 10^-3^ (70/7,495 nuclei) and 4.1 10^-3^ (75/9,403 nuclei) compared to 4.7 10^-3^ (76/8,549 nuclei) and 4 10^-3^ (69/8,805 nuclei) in the transgenic lines, respectively. Thus, after background correction the recombination frequencies in pollen of transgenic 9a and 9b were not statistically different from those observed in pollen of WT plants (Fisher exact test p=0,737 for plant 9a and p=0,907 for plant 9b). Genotyping a higher number of nuclei (from 6,461 to 9,403) led to very similar recombination rate between plant 9a, 9b and WT (5.2 *vs.* 4.7 10^-3^ and 4.1 *vs.* 4 10^-3^ after background correction), thereby confirming the robustness of our pollen typing assays, in the range of 10^-3^ recombinant nuclei. As for plant 7b, the less favorable pre-requisite molecular and functional features observed in the transgenic plants 9a and 9b are likely responsible for the absence of recombination stimulation. Next, we questioned whether an accurate and consistent Chr.9 region typing would have been obtained by using only 3 probes instead of 4, as implemented in the Chr7 assay, in which the probe 1 was estimated to be insufficiently robust. We observed that the recombination rates were similar and consistent in the surveyed Chr. 9 intervals in including or not the probe 1 (**Supplementary Table S1**, **Supplementary Figure S7B, C**). Altogether, we conclude that only the plant 7a exhibited a significant stimulation of meiotic recombination in the targeted region of the Chr.7. This stimulation parallels the high level of expression of the dCas9-SPO11-1 protein and several gRNAs and correlates with the strong binding to the target region observed in this particular transgenic event (**Figures 2B–E**).

### 3.7 Stimulation of targeted recombination in the progeny of the plant 7a

**Figure 6:**
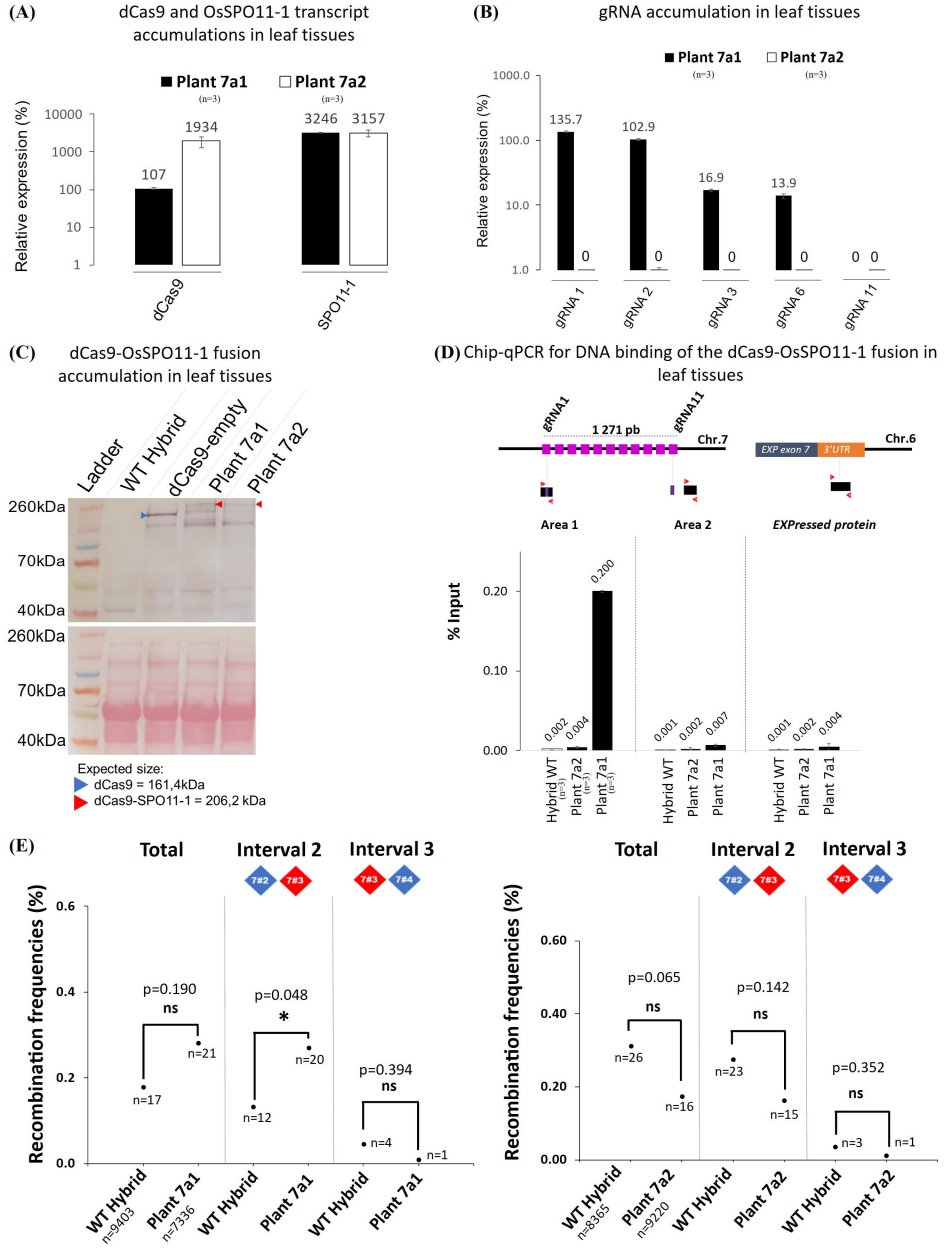
Molecular and functional characterization of the transgenic offspring plants 7a1 and 7a2. **(A)** RT-qPCR quantification of the dCas9 and SPO11-1 transcripts in leaf tissues of the transgenic plants 7a1 and 7a2 relative to *OsKitaake07g010600.1 Expressed protein* gene as reference (n=3). Values follow a Log10 scale. **(B)** RT-qPCR accumulation of the gRNAs in leaf tissues of transgenic plants 7a1 and 7a2. Values follow a Log10 scale and use the Kitaake *Os07g010600.1* gene as a reference. **(C)** dCas9-SPO11-1 accumulation in leaf tissues of plants 7a1 and 7a2 revealed by western blot analysis using an anti-V5 antibody. Expected gel migration positions of dCas9 (161 kDa) and dCas9-SPO11-1 (206 kDa) are pointed by blue and red arrowheads, respectively. **(D)** ChIP-qPCR of the targeted chromosome 7 region. Chromatin DNA of WT hybrid and offspring of the hybrid plants 7a1 and 7a2 were immuno-precipitated using the anti-v5 antibody and quantified by qPCR. The sonicated fragments are in the range of 200-900 bp. Target sequences of the 11 gRNA scaffold are shown in purple. The qPCR amplified regions (area 1 and 2) are indicated by red arrowheads. The enrichment values are normalized by the input (10% of total chromatin). The 3’ region of the ubiquitously expressed gene *OsKitaake06g078500.1* residing on Chr.6 was used as a non-target control (n=3). **(E)** Recombination frequencies over the target region of chromosome 7 deduced from genotyping nuclei WT and dCas9-SPO11-1 plant 7a1 or 7a2 pollen. Values are corrected by removing the background (Methods). Frequencies are compared over the whole region and then over each interval. The detailed data set is reported in **Table 1**. The corrected number of recombinant nuclei is indicated under recombination rate values. Fisher exact test was performed between WT and plant 7a1 or 7a2 (p values are shown, p>0.05*, ns, not significant).

Given the difficulty to obtain another molecularly optimal transgenic plant such as 7a, we investigated whether the Chr. 7 stimulation could be recapitulated in a segregating plant descending from the plant 7a unrecombined at the target locus but still expressing the targeting machinery (dCa9-SPO11-1 fusion and gRNAs). For this purpose, we genotyped 353 F2 progeny plants of plant 7a. To select progeny plants preserving heterozygosity at the Chr.7 markers flanking the target region, we genotyped 8 Kasp markers (4 corresponding to the dPCR markers of the target regions and 4 other markers scattered along the chromosome arm: **Figure 2A**). These markers, the dCas9-SPO11-1 T-DNA integration site and tRNA:gRNA T-DNA integration site segregated independently and in a Mendelian manner, indicating their location at genetically unlinked loci. We selected F2 individuals that were heterozygous for these 8 markers (from ZA to ZD), which means that the entire 7.8 Mb region around the target region is heterozygous similar to plant 7a and has not undergone recombination event preventing potential biases in the analysis of recombination in these offspring. Then, following the same pipeline previously used to screen optimal molecular features, we examined 70 plant progenies step by step to finally select the best plant 7a1 that, alike the parent F1 plant 7a, carries and expresses both transgenes, exhibits a high expression level for the gRNA-1 and -2 and a robust ChIP-PCR binding of the dCas9-SPO11-1 protein in the vicinity of these gRNA targets (**Figures 6A–D**). As a negative control, we retained another progeny plant (7a2) carrying the dCas9-SPO11-1 T-DNA, but negative in PCR amplification and expression of the tRNA:gRNA T-DNA as well as binding of dCas9-SPO11-1 to chromatin (**Figures 6A–D**). Of note, among the 353 F2 progenies, 1 plant exhibited recombination between markers 7#2 and 7#3 indicating that recombination event detected by pollen typing in chromosome 7 yields a viable plant. Sanger sequencing confirmed the occurrence of a crossing over event that mapped within a 1,624 bp interval located upstream of the edging gRNA-1 target site.

Then, upon dPCR genotyping of pollen nuclei and background correction, a higher but not statistically significant (p=0.19) number of recombinants was observed in progeny plant 7a1 compared to WT control over the whole targeted region (2.8 10^-3^ in transgenic 7a1 *vs* 1.8 10^-3^ in WT) (**Figure 6E**). However, a moderate but significant 2.05-fold stimulation (p=0.048) was observed in the interval 2 (marker #2-3), containing all the gRNA target sites. No stimulation was detected on the adjacent interval 3 (p=0.388) (**Figure 6E**, **Table 1**). Then, as expected, no significant stimulation of recombinant nuclei was observed in the progeny of the 7a2 plant which carried the dCas9-SPO11-1 T-DNA but not the gRNAs transgene. (**Figure 6E**, **Table 1**).

In conclusion, compared to wild type and several negative controls, we conclude that the significant increase of recombinant nuclei observed in the F1 transgenic plants 7a is replicated in the segregant plant 7a1 and associated with optimal expression and binding of the dCas9-SPO11-1 fusion protein at the targeted locus.

## 4 Discussion

Despite the high interest in engineering targeted recombination in crops, few studies have been reported yet. Inducing chromosomal DSB with active CRISPR/Cas9 systems in tomato and maize somatic cells can lead to homology-directed repair resulting in recombination events that can be transmitted to offspring through the germline (Filler Hayut et al., 2017; Kouranov et al., 2022). However, the low frequency of transmission suggests that achieving targeted recombination during meiosis could be a more robust and efficient strategy. Our solution was to implement the dCas9-SPO11-1 strategy developed in yeast (Sarno et al., 2017) in order to locally increase the recombination frequency *via* the nearby stimulation of Spo11-dependent meiotic DSBs. In that aim, we built a series of dCas9-SPO11-1 transgenic hybrid lines, conducted several prerequisite molecular and functional assays to screen the potential recombination-prone lines, improved the sensitivity of the pollen typing method and assayed the stimulation of meiotic recombination in two interstitial chromosomal regions. Based on pollen nuclei genotyping, the optimal transgenic line 7a exhibited a statistically significant 3.27-fold stimulation of recombination in the targeted chromosomal region of ~ 22 Kbp. Importantly, the progeny plant 7a1 also displayed a significant recombination increase, with a 2.05-fold stimulation in the interval 2 containing the targeted sites. This stimulation falls within the range of CO increase observed in yeast using a similar technology, which also identified genomic regions amenable or refractory to stimulation (Sarno et al., 2017). To our knowledge, this is the first report of a biotechnological method to locally stimulate meiotic recombination in plants.

The experimental strategy described here covered several technical choices. The first critical question was how to achieve the functional expression of the dCas9-SPO11-1 fusion protein. First, we choose to use a genomic *OsSPO11-1* DNA sequence instead of a shorter putative *OsSPO11-1* cDNA that would be beneficial to reduce the size of this large T-DNA and allow the construction of a single dCas9-SPO11-1 + gRNAs T-DNA, but at risk to eliminate potentially relevant alternative SPO11-1 forms. Second, we added two linker amino acid sequences (V5-NLS, dCas9 and SPO11-1) in order to limit the potential structural interference between the functional domains. The drawback may have been to limit the amount of fusion protein *per* cell by triggering transcriptional abnormalities and *in vivo* proteolysis of the fusion protein. Along these lines, although we observed full complementation of the *Osspo11-1* mutant sterility, it also remains possible that despite the robustness of the linkers, a small amount of full length SPO11-1 protein contributes to the recovery of viable progenies (**Figure 2C**, **Supplementary Figure S1C**, **Figure 6C**). This uncertainty is minimized here by the use of a SPO11-1 wild type background in all the other experiments. One might consider that the expression of the fusion protein under the constitutive *UBI* promoter leads to an over-expression of the Spo11 complex sufficient to increase unassisted binding to the target site and thereof stimulation of recombination (**Figure 2E**, **Supplementary Figures S1E**, **Figure 6D**). This is unlikely, since no stimulation of recombination was observed in our transgenic plant that expressed the dCas9-SPO11-1 fusion in the absence of the gRNAs transgene (**Figure 6E**). Mechanistically, we expect that targeted DSB formation depends on the activity of a multimeric dCas9-SPO11-1 complex but since the Spo11 complex likely associates in large amount at natural DSB sites (Claeys Bouuaert et al., 2021b, Claeys Bouuaert et al., 2021a), it is possible that the dCas9-SPO11-1 protein complex associates with endogenous SPO11 proteins, sufficient to trigger targeted DSBs.

Third, we constitutively expressed the fusion protein instead of using a meiosis-specific promoter potentially able to fine tune the level and the time window of expression. However, the probability to induce a Spo11-dependent nuclease activity in somatic tissues remains unlikely since DSB formation is dependent on several other meiosis-specific proteins. Consistently, no adverse effect on the phenotype has been noted in transgenic rice plants constitutively accumulating the dCas9-SPO11-1 fusion, not different from the observations in yeast and *Arabidopsis*, accumulating dCas9-Spo11 (Sarno et al., 2017; present results) or MTOPVIB-dCas9 (Yelina et al., 2022), respectively.

The second critical set of experimental choices was how to express the gRNAs. Here, we used the tRNA:gRNA vector system (Xie et al., 2015) that allows to express multiple gRNAs carried on a single transgene. The rationale was to ensure the expression of at least one gRNA (preferentially several), able to tether dCas9-SPO11-1 protein at the target site. Hence, the targeting efficiency in a given region will be boosted and the chromosomal targets diversified. Along this line, we first validated the expression of the gRNAs using the live Cas9 mutagenic assay in callus cells. However, as previously observed in *Arabidopsis* (Yelina et al., 2022) and rice (Xie et al., 2015), we observed that only a subset of gRNA transcripts (3-5/11 tRNA:gRNA scaffold present in the T-DNAs) accumulated in an extremely variable Log10 amount but nevertheless validated the benefit of the multiplex tRNA:gRNA expression system. Most importantly, it was consistent that the most expressed gRNA-1 and -2 in plants 7a and 7a1 mapped in the region where we found the highest dCas9-SPO11-1 residency in the ChIP assay (**Figures 2D, E** and **Figures 6B, D**). This result suggests that the abundance of gRNAs has an impact on the efficiency of dCas9-SPO11-1 protein binding. It would therefore be more effective to develop systems that produce few but highly expressed gRNAs, rather than systems that produce many gRNAs with low and variable levels of expression. In this study, we chose to position the gRNAs in the 5’ or 3’ UTR regions, partly because of gRNA design constraints, but also because chromatin is generally more open/accessible in these regions and prone to meiotic DSB (Tock and Henderson, 2018). On the other hand, it would be interesting to attempt to position gRNAs in more condensed regions, particularly if we are aiming to induce recombination in pericentromeric areas with high levels of heterochromatin. However, at this stage we have no data to predict the optimal position for gRNA targets.

The third critical issue of our experimental approach is the capacity to measure rare recombination events since the handling of several thousand control and treatment progeny plants is a strong limiting factor. Hence, to rapidly estimate meiotic recombination frequencies, the most appealing, generic, and cost-efficient alternative is to genotype individual pollen nuclei. Recently in barley, Ahn et al. (2021) developed a high-throughput dPCR method (Crystal Digital PCR ™) to genotype a large number of flow-sorted pollen nuclei and robustly detect recombinant molecules within four large genomic regions of 12.2Mbp up to 344.7 Mbp that spanned 6.7 up to 11.7 cM. Differently here, we sorted out rice pollen nuclei with FACSAria and used the QIAcuity Digital PCR platform (Qiagen) with 4 fluorescent interspaced probes (2 for each parental genome) located in two small regions of the Chr.7 and 9 (~22 Kbp and ~58kbp, respectively). This led to reach a genotyping sensitivity in the range of 10^-3^ nuclei. Similar to the barley pollen genotyping (Ahn et al., 2021), we found a variable background noise from one experiment to another, here measured in the range of 1-2 10^-3^ nuclei. Thus, being in the range of the observed recombination frequency in short intervals, it becomes essential to process the background, wild-type control, and dCas9-SPO11-1 transgenic plant pollen nuclei assays in parallel and stringently filter the fully genotyped nuclei in order to define the cutoff of signals *vs.* noise. We observed a 1.97-fold stimulation of the recombination frequency in the dCas9-SPO11-1 plant 7a over wild-type (5.3 10^-3^ *vs.* 2.7 10^-3^, p=0.012), raised to a 3.27-fold stimulation after background correction (3.8 10^-3^ *vs.* 1.2 10^-3^, p<0.001, respectively) (**Figure 5B**, **Table 1**). Stimulation was confirmed in the offspring plant 7a1 but only in the interval 2 (1.3 10^-3^ *vs.* 2.7 10^-3^, p=0.048) containing the gRNAs binding sites (**Figure 6E**, **Table 1**). Thus, our targeting method provided a detection threshold precise enough to properly differentiate recombination frequencies in small intervals down to few Kbp.

In contrast, to the present dCas9-SPO11-1 fusion that stimulated recombination in 1 out of 2 tested regions, the dCas9-MTOPVIB fusion did not stimulate targeted crossovers at the 3a crossover hotspot locus in *Arabidopsis* (Yelina et al., 2022). As thoroughly discussed by these authors, there are multiple technical and biological explanations. One hypothesis is the observation in *S. cerevisiae* that the efficacy of meiotic DSB formation by the dCas9-SPO11-1 fusion protein - and Spo11 fusion to other DNA binding modules - differs from one site to another (Sarno et al., 2017). The other key limiting factor in plants and most organisms is that only ~5-10% of DSBs ultimately mature into a CO (Mercier et al., 2015; Lambing et al., 2017). The advantage of our tRNA-gRNA multiplexing method is to facilitate the assay at once of different chromosomal regions.

Although plant to plant variability exists, the observed inheritance of the stimulation effect in the plant progeny not only offered a confirmation opportunity but also opened the production of more plant materials for subsequent studies. Differently, one can also envisage the fusion of dCas9 to SPO11-2 or to other protein components of the Spo11 core complex and more indirectly their interactors (Koehn et al., 2009; Acquaviva et al., 2013; Vrielynck et al., 2021). The warmable Chr.7 region is suited to sensitively assay other target sites and protein fusions.

In conclusion, this study is the first report of a targeted meiotic recombination stimulation in plants and demonstration of its heritability in the progeny. Considering the bottleneck that constitutes the low frequency of meiotic recombination in plants (alike in most eukaryotes), the recovery of few more targeted recombinants in small intervals would be highly valuable in plant breeding, for example in order to exquisitely deconvolute a cluster of nearby polymorphisms, recombine flanking chromosomal regions, resolve linkage drags and achieve introgression of agronomically useful alleles in elite lines.

## Data Availability

The original contributions presented in the study are included in the article/**Supplementary Material**. Further inquiries can be directed to the corresponding authors.
