# Supplementary material for: dCas9-SPO11-1 locally stimulates meiotic recombination in rice
Source: Front Plant Sci. 2025 May 1;16:1580225. doi: 10.3389/fpls.2025.1580225 (PMC12078263; doi:10.3389/fpls.2025.1580225)
Supplement: Supplementary file 1 [file DataSheet1.pdf]

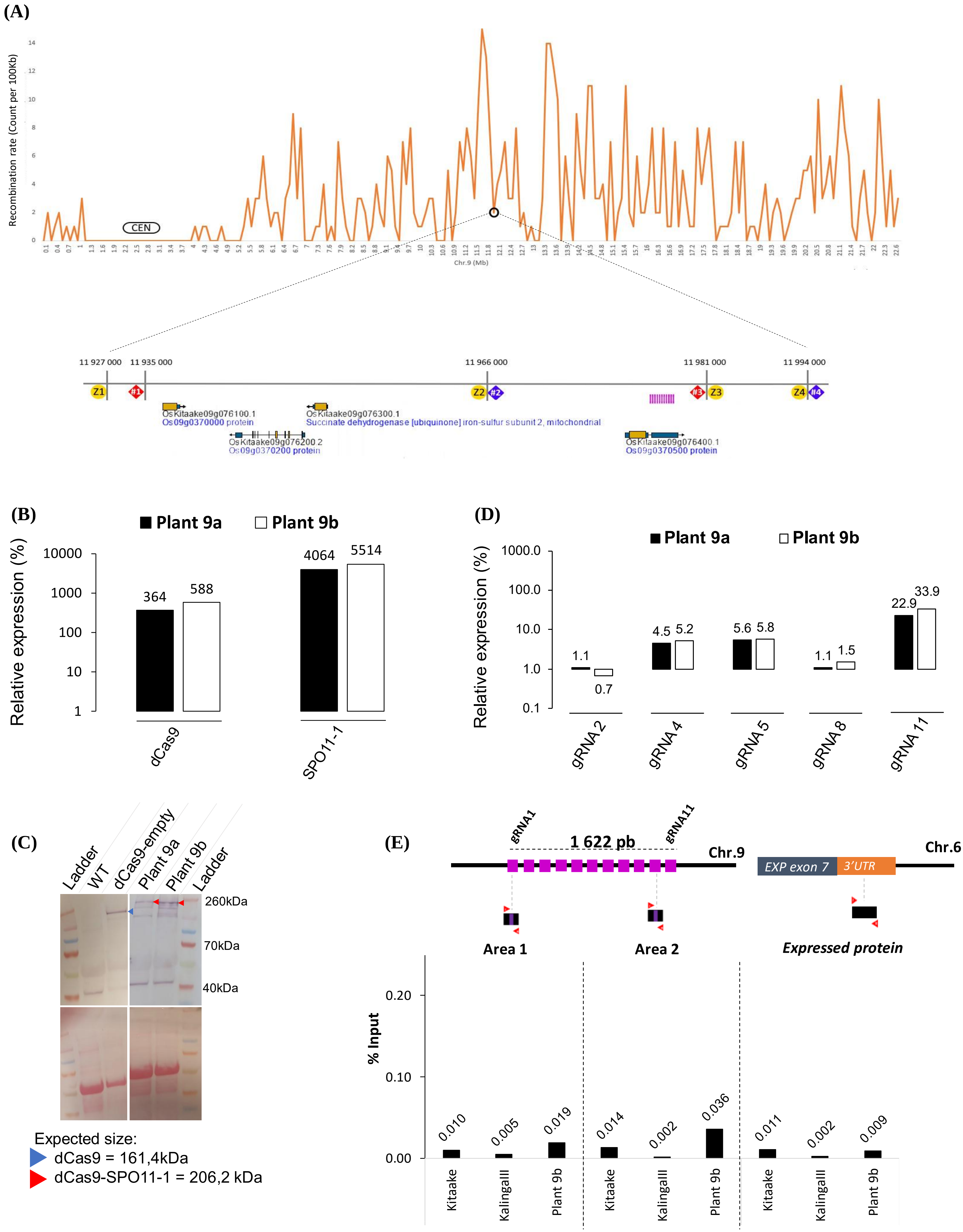

Supplementary Figure 1.

Supplementary Figure 1: Molecular and functional characterization of the transgenic plants 9a and 9b.

(A) Schematic representation of the recombination context of the targeted Chr.9 region established by whole genome sequencing of a 379 F2 progeny population of KalingaIII/Kitaake (Unpublished). The position of the dPCR (9#1, 9#2, 9#3 and 9#4) and Kasp (Z1, Z2, Z3 and Z4) markers with their coordinates on the Kitaake genome are detailed on the zoom. The sequence of the polymorphic markers and 11 gRNAs (pink bars) are given in **Tables S4** and **S9**. The 11 gRNAs are in the 3' sequence of the *Os09g076400.1* gene. **(B)** RT-qPCR quantification of the dCas9 and SPO11-1 cDNAs from the transgenic KalingaIII/Kitaake plants 9a and 9b relative to Kitaake *Os07g010600.1 Expressed protein* gene as reference. Values follow a Log10 scale. **(C)** dCas9-SPO11-1 accumulation in leaf tissues of plants 9a and 9b revealed by western blot analysis using an anti-V5 antibody. Expected gel migration positions of dCas9 (161 kDa) and dCas9-SPO11-1 (206 kDa) are pointed by blue and red arrowheads, respectively. **(D)** RT-qPCR accumulation of the gRNAs in leaf tissues of transgenic plants 9a and 9b. Values follow a Log10 scale and use the Kitaake *Os07g010600.1* gene as a reference. **(E)** ChIP-qPCR of the targeted chromosome 9 region. Chromatin DNA of WT KalingaIII and Kitaake parents and independent transgenic KalingaIII/Kitaake hybrid plant 9b were immuno-precipitated using the anti-v5 antibody and quantified by qPCR. The sonicated fragments are in the range of 200-900 bp. Target sequences of the 11 gRNA scaffold in purple. The qPCR amplified regions (area 1 and 2) are indicated by red arrowheads. The enrichment values are normalized by the input (10% of total chromatin). The 3' region of the ubiquitously expressed gene *Os06g078500.1* residing on Chr.6 was used as a non-target control.
