# Supplementary material for: dCas9-SPO11-1 locally stimulates meiotic recombination in rice
Source: Front Plant Sci. 2025 May 1;16:1580225. doi: 10.3389/fpls.2025.1580225 (PMC12078263; doi:10.3389/fpls.2025.1580225)
Supplement: Supplementary file 2 [file DataSheet2.pdf]

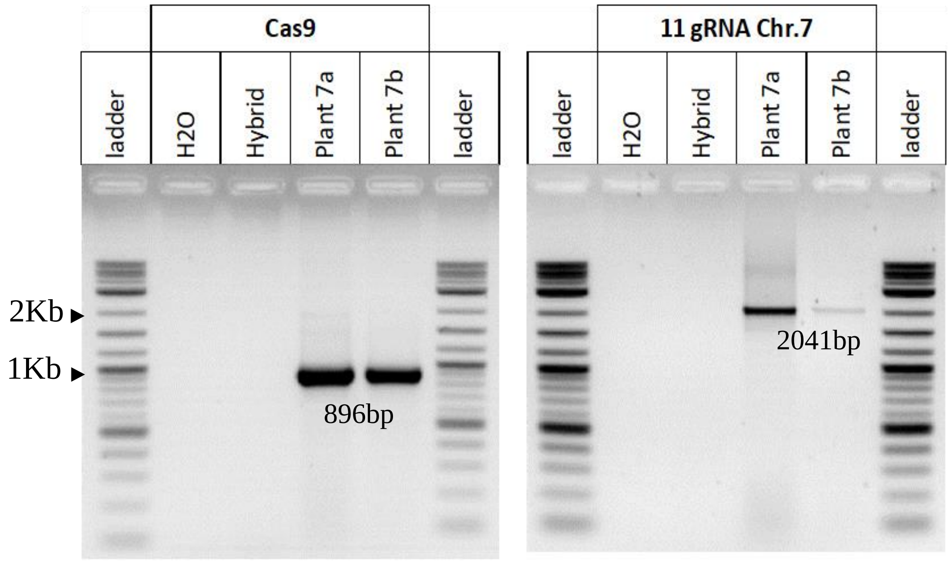


**Supplementary Figure 2: Detection of the T-DNA constructs by PCR.**
PCR amplification of the dCas9 (896 bp) (right) and 11 gRNA (2,041 bp) (left) regions of the T-DNA in transgenic plants 7a and 7b. Parental lines of the KalingaIII/Kitaake hybrid are used as non transgenic controls.
