# Supplementary material for: dCas9-SPO11-1 locally stimulates meiotic recombination in rice
Source: Front Plant Sci. 2025 May 1;16:1580225. doi: 10.3389/fpls.2025.1580225 (PMC12078263; doi:10.3389/fpls.2025.1580225)
Supplement: Supplementary file 3 [file DataSheet3.pdf]

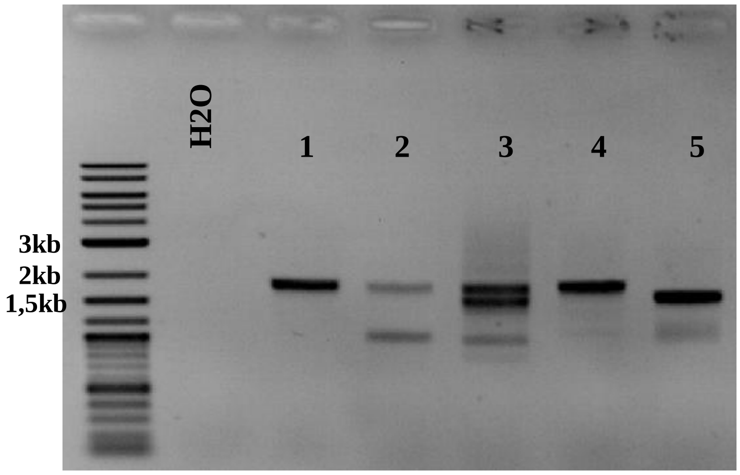

Supplementary Figure 3: Ability of the 6 first gRNAs at inducing DSB when accumulated with an active Cas9.

Amplification of the targeted Chr.7 region was performed in putative transgenic (1-5) calluses of cv. Kitaake (using an antibiotic selection medium) accumulating an active Cas9 and the first 6 gRNAs specific to the target region. Expected size of the amplicon is 1.895 bp. Several deletion profiles are observed. Sanger sequencing confirmed deletion between targets of gRNA-1 and -6. However, due to the high efficiency of the Cas9 system and vicinity of the targets, other deletions and frameshift mutations are observed with no clear identification of the responsible gRNA.
