# Supplementary material for: dCas9-SPO11-1 locally stimulates meiotic recombination in rice
Source: Front Plant Sci. 2025 May 1;16:1580225. doi: 10.3389/fpls.2025.1580225 (PMC12078263; doi:10.3389/fpls.2025.1580225)
Supplement: Supplementary file 4 [file DataSheet4.pdf]

(A)

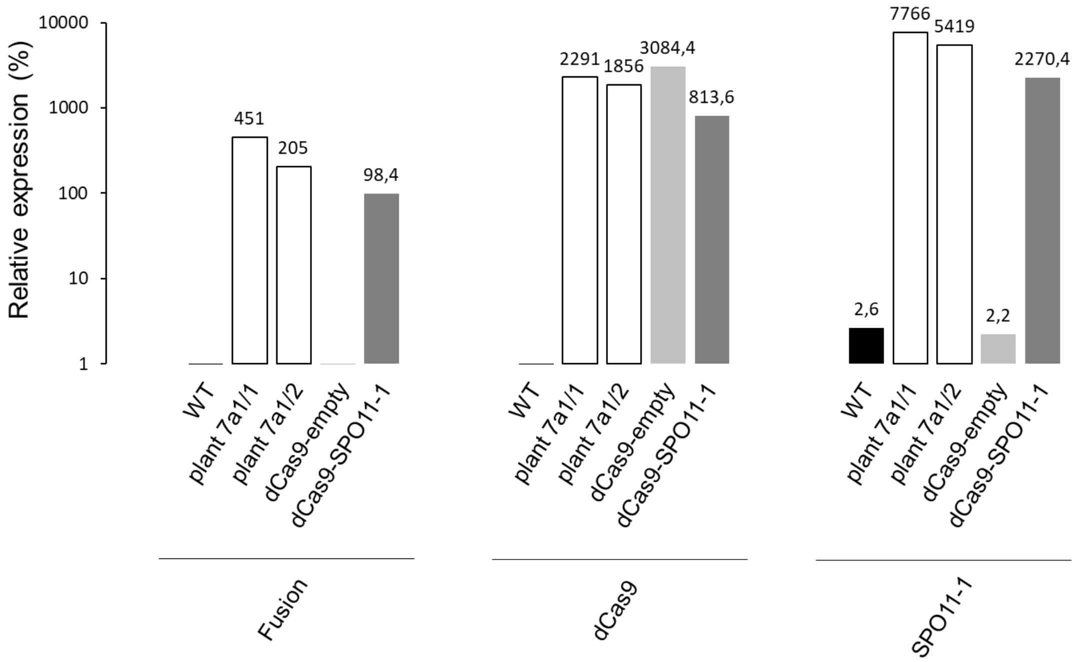

(B)

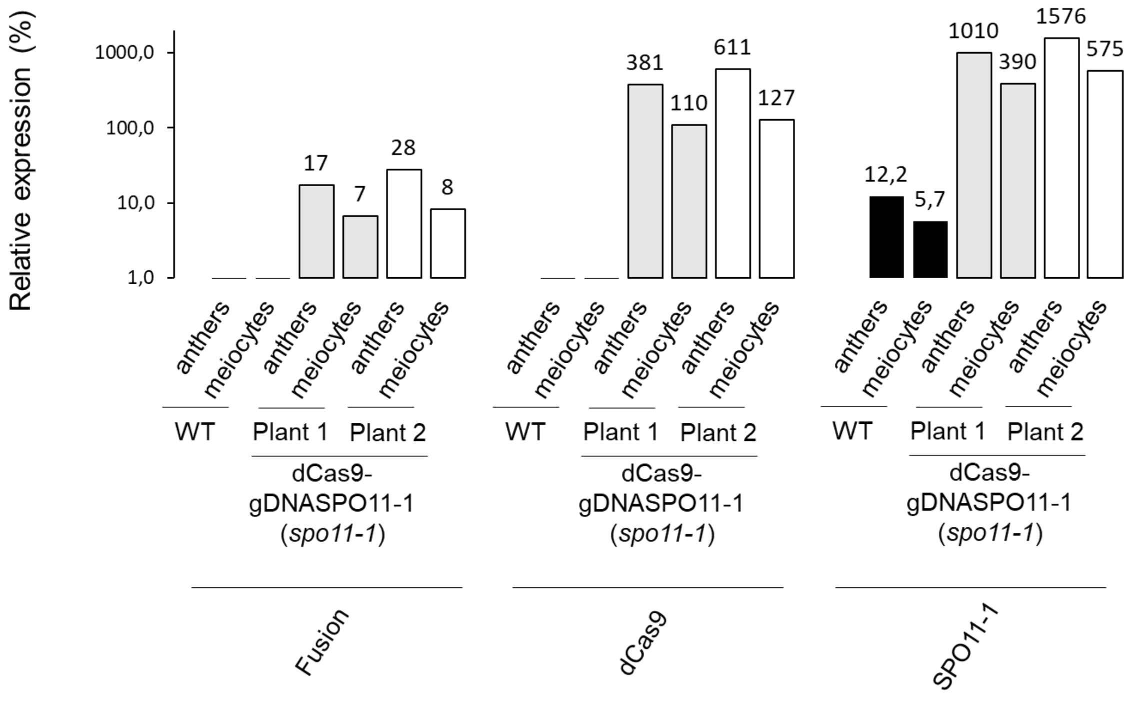

Supplementary Figure 4 : Expression of the dCas9-SPO11-1

(A) RT-qPCR quantification of two descendants of hybrid plant 7a1 (7a1/1, 7a1/2) compared to WT, dCas9-empty and dCas9-SPO11-1 *spo11-1* plants transcripts in leaf tissues relative to Kitaake *Os07g010600.1 Expressed protein* gene as reference. Amplifications were performed using primers located on dCas9, on SPO11-1 and on the interval between the end of dCas9 and SPO11-1 including the linker (Fusion). Values follow a Log10 scale. **(B)** RT-qPCR quantification of two dCas9-SPO11-1 *spo11-1* kitaake mutant plants transcripts in anthers and meiocytes tissues relative to Kitaake *Os07g010600.1 Expressed protein* gene as reference. Amplifications were performed using primers located on dCas9, on SPO11-1 and on the interval between the end of dCas9 and SPO11-1 including the linker (Fusion). Values follow a Log10 scale.
