# Supplementary material for: dCas9-SPO11-1 locally stimulates meiotic recombination in rice
Source: Front Plant Sci. 2025 May 1;16:1580225. doi: 10.3389/fpls.2025.1580225 (PMC12078263; doi:10.3389/fpls.2025.1580225)
Supplement: Supplementary file 5 [file DataSheet5.pdf]

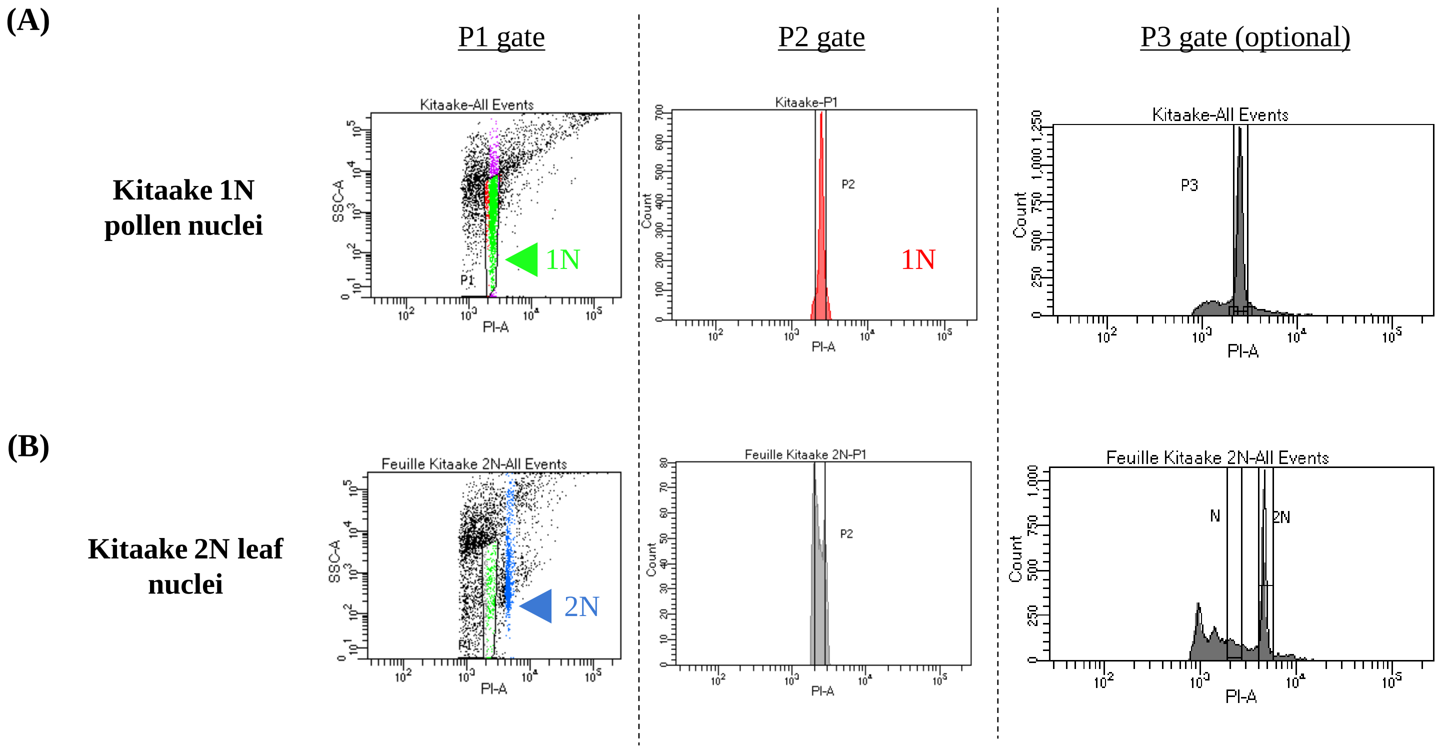

Supplementary Figure 5: Extraction and sorting of nuclei from pollen and leaf tissue with Facs Aria.

Nuclei were extracted at 4°C in filter-sterilized LB01 buffer (15mM TRIS (Sigma Trizma 7-9, 99%), 2mM Na2EDTA, 80mM KCl, 20mM NaCl, 0.5mM Spermine, 40mM Na2SO3, 0.10% Triton X100, pH 7.5). Anthers were vortexed 2 min with 1 mL LB01 then centrifuged at 2,790 g at 4°C for 5 min. The tube was vortexed briefly and the solution was filtered through a 100µm filter (CellTrics 100µm ; 04-0042-2318 ; Partec) in a new 2mL tube. Eluate was centrifugated 3 min at 150 g at 4°C. Supernatant was replaced with 1 mL of fresh LB01. After briefly vortexing, the solution was further filtered (CellTrics 20µm ; 04-0042-2315 ; Partec) in a new 2mL tube. Pollen grains were grinded on the filter with a glass pestle. Using the same tube and filter, the eluate was filtered again. These two steps were repeated once. Eluate was filtered on a new clean filter 20 µm on 15 mL tube. The final volume was adjusted to 1 mL with LB01, and kept on ice in the dark. Prior to sorting, 42 µL of PI (1mg/mL ; Ref 3566 Life Technologies) was added to stain the nucleus preparation.
For leaf nuclei, one cm2 of rice leaf was finely chopped with a razor blade in 2 mL of LB01 buffer at 4°C. The solution was resuspended by pipetting and filtered (CellTrics 30µm ; 04-0042-2316 ; Partec). Prior to sorting, 42 µL of PI (1mg/mL ; Ref 3566 Life Technologies) was added to the nucleus suspension.

Kitaake pollen **(A)** or leaf nuclei **(B)** were sorted according to their Propidium Iodide (PI-A) marking intensity using a P1 gate. They are followed by a P2 refinement gate between $10^{3.1}$ and $10^{3.2}$ for 1N pollen nuclei. Gate P3 shows the distribution of all the detected events.
