# Supplementary material for: dCas9-SPO11-1 locally stimulates meiotic recombination in rice
Source: Front Plant Sci. 2025 May 1;16:1580225. doi: 10.3389/fpls.2025.1580225 (PMC12078263; doi:10.3389/fpls.2025.1580225)
Supplement: Supplementary file 6 [file DataSheet6.pdf]

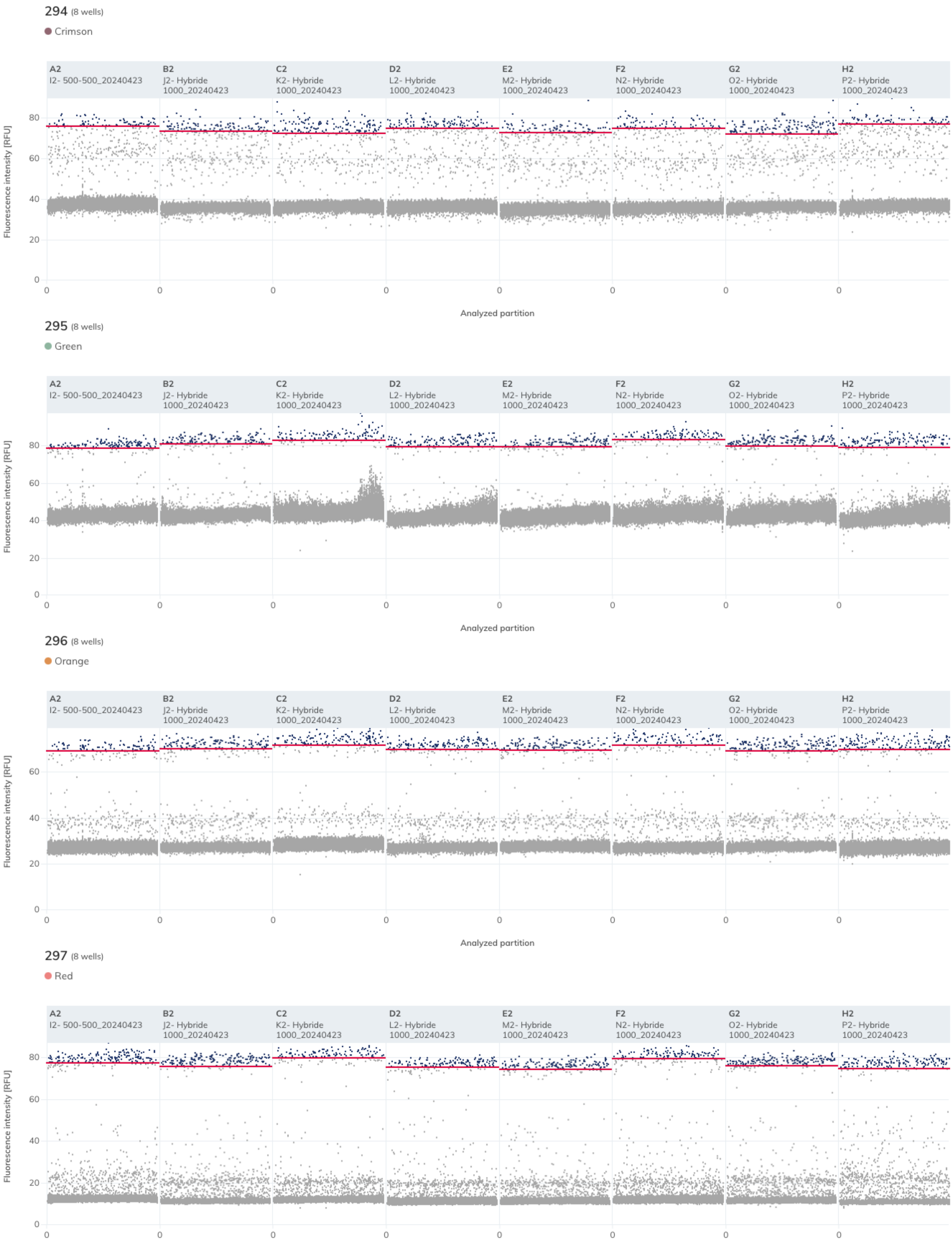

Supplementary Figure 6 (A)

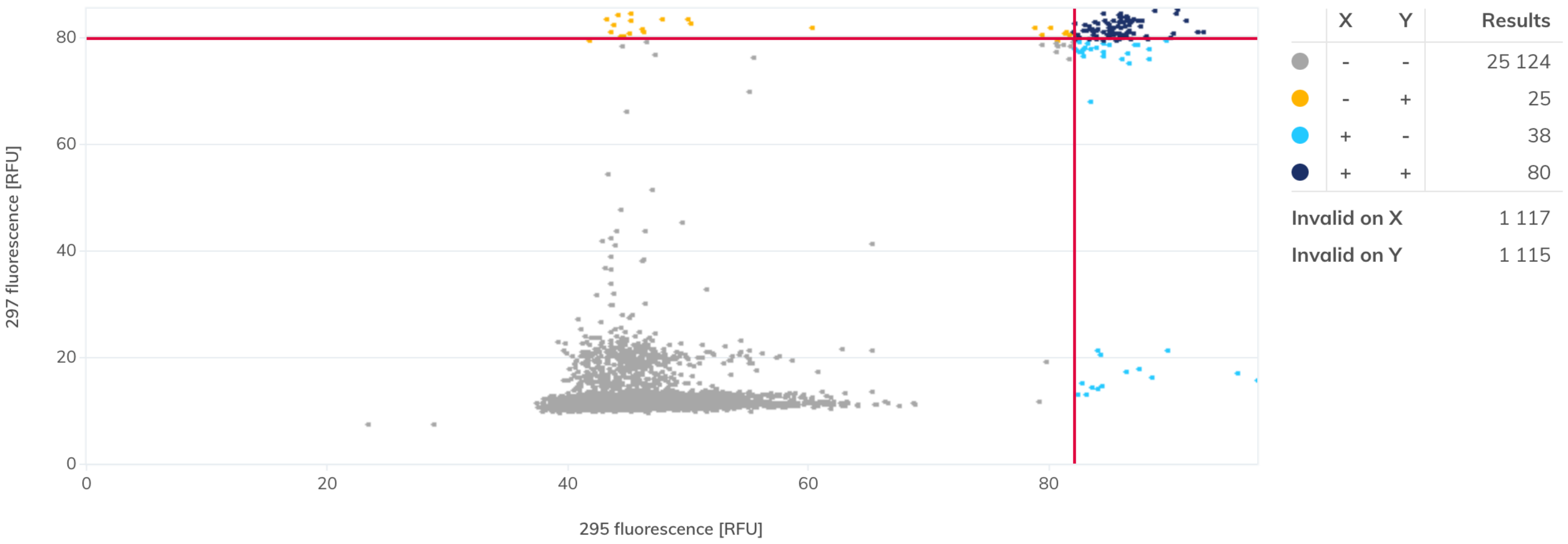

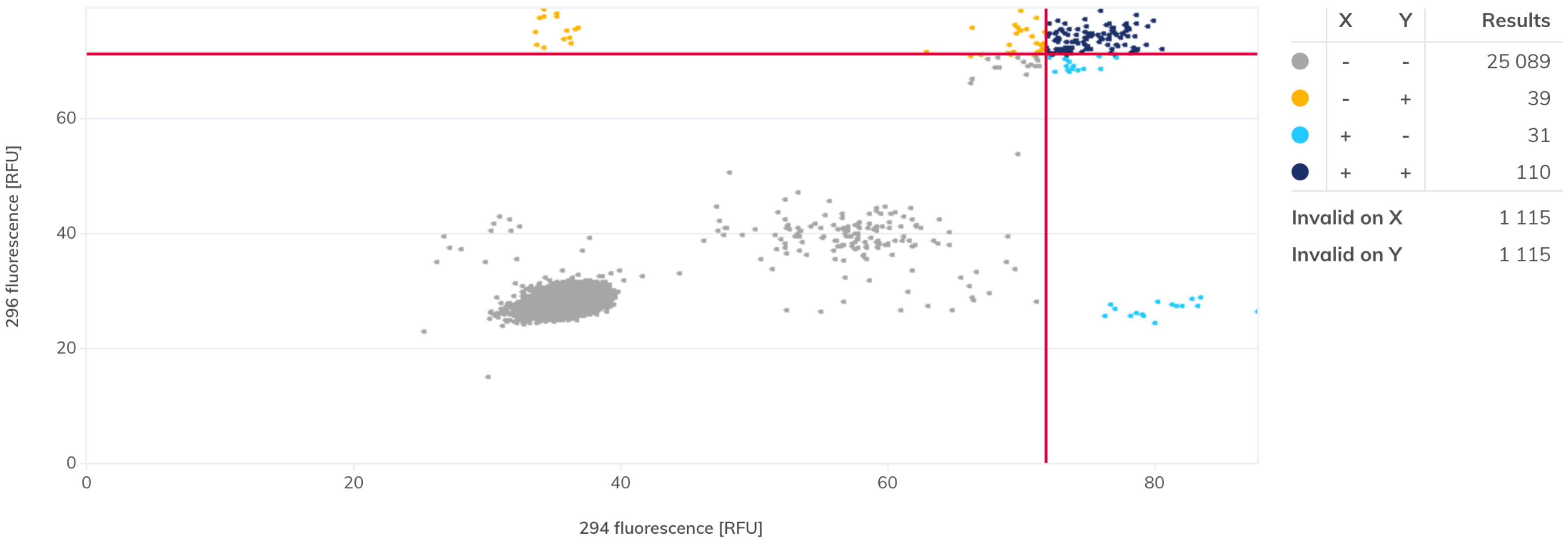

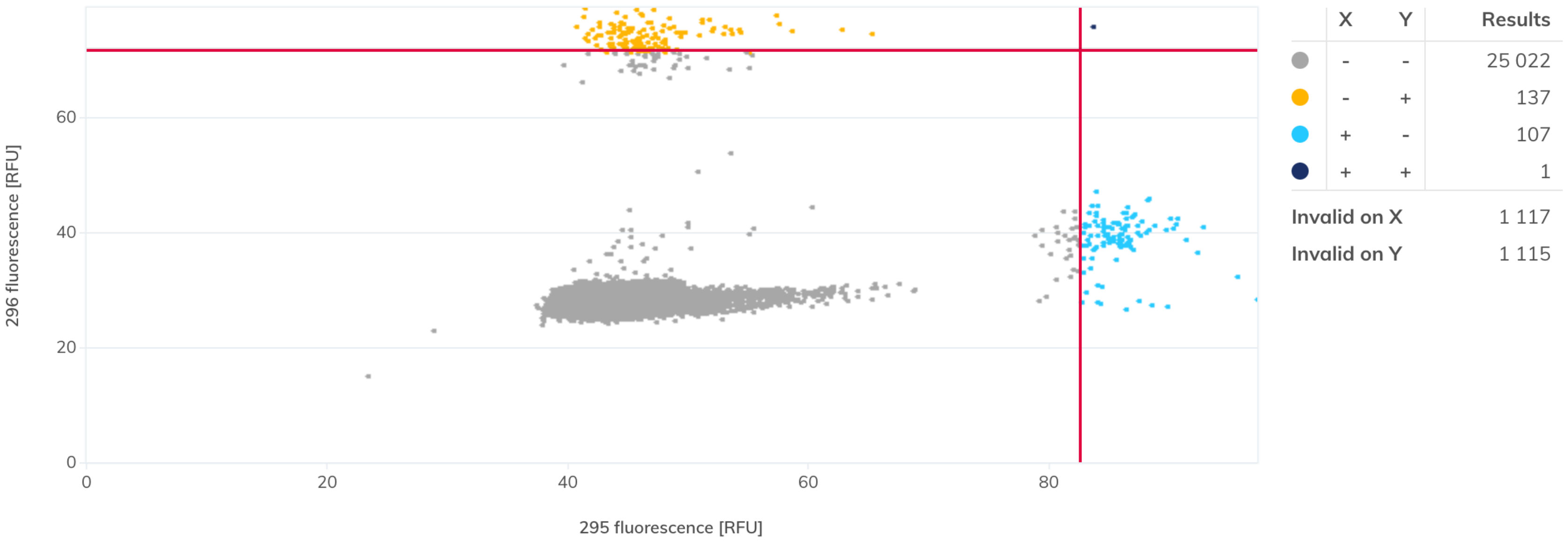

Supplementary Figure 6 (B) (part1/2).

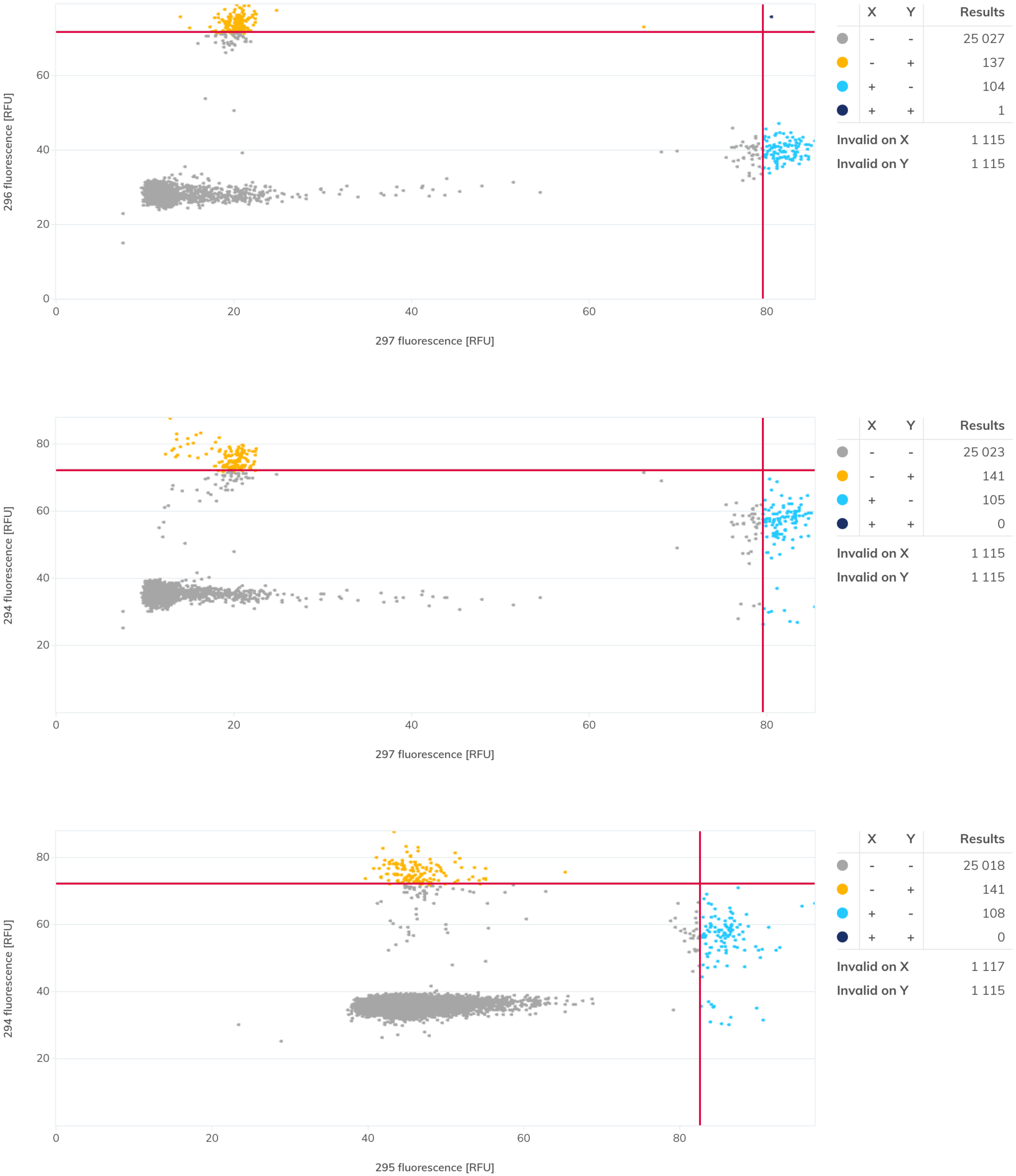

Supplementary Figure 6 (B) (part2/2).

Supplementary Figure 6: Example of dPCR analysis of pollen nuclei.

As described in the **Methods**, the probes were designed and tested in collaboration with IAGE one by one, then in associations first using Kitaake and KalingaIII DNA and then on pollen nuclei. The dPCR output analyses described here (setting thresholds and counting individuals) were carried out only by IAGE company. Sorted rice pollen nuclei (**Figure S5**) are distributed in dPCR wells as described in **Figure 3**. Probes 7#1, 7#2, 7#3, 7#4 correspond to the fluorescence intensity 294, 295, 296 and 297 respectively.

(A) Example of analysis of 7 dPCR wells (500-500 or WT hybrids, A2 to H2). First, thresholds have been set independently for each well just below the highest florescence intensity cluster for the 4 probes.

(B) Example of analysis of the C2 well. Then, 2D thresholds have been adjusted stringently (very close to the center of the fluorescence cluster of each allele) for each probe associations. The analyses show that the 2D clusters separate perfectly for probe associations 7#2, 7#3 and 7#4, but much less precisely with probe 7#1. The weakest association is observed with probes 7#1 and 7#2, which explains why we did not use it to analyze recombination. In contrast, the association of probes 7#1 and 7#3, although weak, clearly distinguishes the two alleles enabling us to assess the number of Kitaake individuals. In this C2 well, two recombinant pollen nuclei are observed: one between 7#2 and 7#3 and one between 7#3 and 7#4.

Note that adjusting thresholds in 2D automatically readjusts the thresholds observed in **(A)**.
