# Supplementary material for: dCas9-SPO11-1 locally stimulates meiotic recombination in rice
Source: Front Plant Sci. 2025 May 1;16:1580225. doi: 10.3389/fpls.2025.1580225 (PMC12078263; doi:10.3389/fpls.2025.1580225)
Supplement: Supplementary file 7 [file DataSheet7.pdf]

(A)

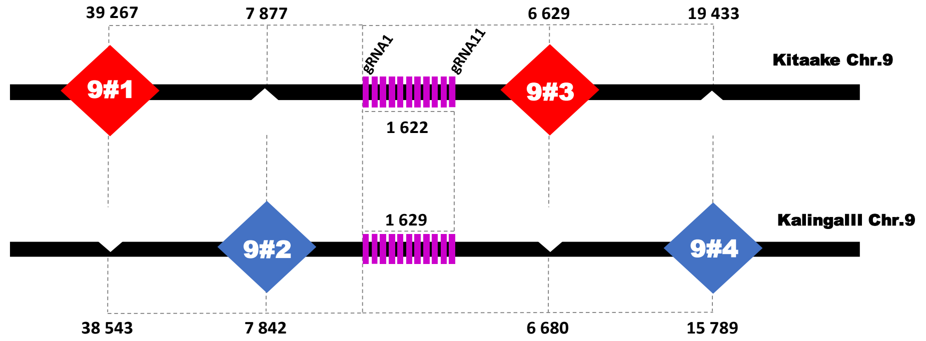

Probe name and Fluorochrome	Sequence on Kitaake	Sequence on KalingaIII
9#1 Cy5	G[818pb]CAGGGGCATATAGGGAA TTTTCTCTTAGTT[443pb]C	GATAGAC
9#2 FAM	ATACTAAAACAATGGGAAGTACAT TTGCAA	ATGCTAAAACAATGGGAATGG GAAGTACAT
9#3 TAMRA	GATACTTAAAGG	GATACAAAGG
9#4 ROX	AAAATGTTTGAAACT	TACAATGTTTGACACT

(B)

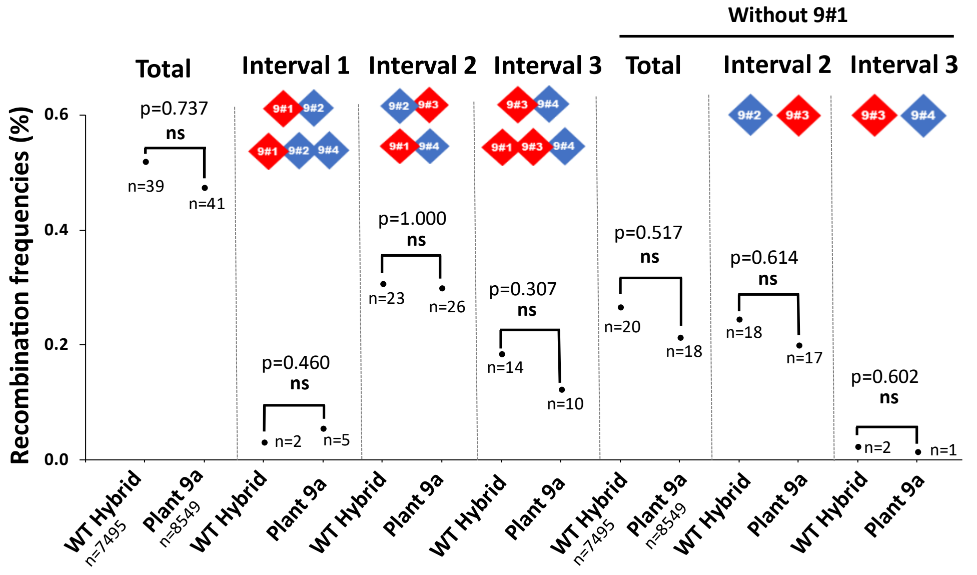

(C)

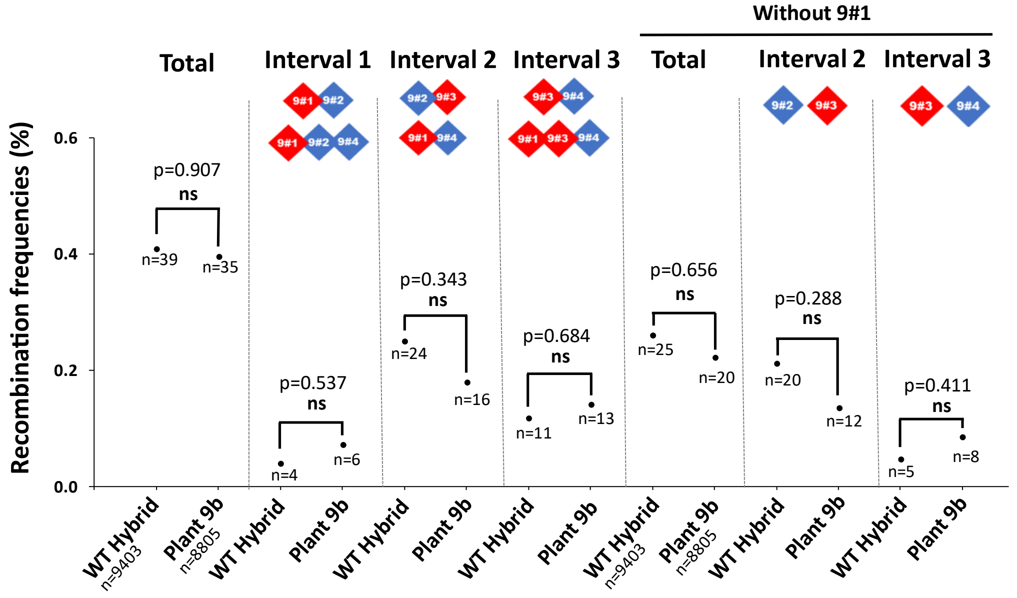

Supplementary Figure 7

Supplementary Figure 7: Recombination frequencies in the dCas9-SPO11-1 events and controls.
(A) Positions of the KalingaIII and Kitaake markers flanking the targeted Chr.9 region. Probes are designed using the SNP/Indels shown in the table. Physical distances are given after the first gRNA position (11,974,932 on the Kitaake Chr.9).
(B-C). Recombination frequencies over the target region of chromosome 9 deduced from genotyping nuclei from WT and dCas9-SPO11-1 plants 9a and 9b pollen. Values are corrected by removing the background (**Methods**). Frequencies are compared over the whole region and then over each interval, using or not information of probe 9#1. , Frequencies are compared over the whole region and then over each interval. The detailed data set is reported in **Table S1**. The corrected number of recombinant nuclei is indicated under recombination rate values. Fisher exact test was performed between WT and plants 9a and 9b (p values are shown, ns: not significant).
