# Supplementary material for: dCas9-SPO11-1 locally stimulates meiotic recombination in rice
Source: Front Plant Sci. 2025 May 1;16:1580225. doi: 10.3389/fpls.2025.1580225 (PMC12078263; doi:10.3389/fpls.2025.1580225)
Supplement: Supplementary file 8 [file DataSheet8.pdf]

Supplementary Figure 8: Sequences used in T-DNAs.

pZmUbi1-dCas9-gSPO11-1-tNos

Legend : pZmUbi1, V5, NLS, Linkers, dCas9, gDNA-SPO11-1, tNos

aagcttgcatgcctgcagtgcagcgtgacccggtcgtgcccctctctagagataatgagcattgcatgtctaagttataa
aaaattaccacatatttttttgtcacacttgtttgaagtgcagtttatctatctttatacatatatttaaactttactc
tacgaataatataatctatagtactacaataatatcagtgttttagagaatcatataaatgaacagttagacatggtcta
aaggacaattgagtattttgacaacaggactctacagttttatctttttagtgtgcatgtgttctcctttttttttgcaa
atagcttcacctatataatacttcatccattttattagtacatccatttagggtttagggttaatggtttttatagacta
atttttttagtacatctattttattctattttagcctctaaattaagaaaactaaaactctattttagtttttttattta
ataatttagatataaaatagaataaaataaagtgactaaaaattaaacaaatacccttttaagaaattaaaaaaactaagg
aaacatttttcttgtttcgagtagataatgccagcctgttaaacgccgtcgacgagtctaacggacaccaaccagcgaac
cagcagcgtcgcgtcgggccaagcgaagcagacggcacggcatctctgtcgctgcctctggacccctctcgagagttccg
ctccaccgttggacttgctccgctgtcggcatccagaaattgcgtggcggagcggcagacgtgagccggcacggcaggcg
gcctcctcctcctctcacggcacggcagctacgggggattcctttcccaccgctccttcgctttcccttcctcgcccgcc
gtaataaatagacaccccctccacaccctctttccccaacctcgtgttgttcggagcgcacacacacacaaccagatctc
ccccaaatccacccgtcggcacctccgcttcaaggtacgccgctcgtcctcccccccccccctctctaccttctctaga
tcggcgttccggtccatggttagggcccggtagttctacttctgttcatgtttgtgttagatccgtgtttgtgttagatc
cgtgctgctagcgttcgtacacggatgcgacctgtacgtcagacacgttctgattgctaacttgccagtgtttctctttg
gggaatcctgggatggctctagccgttccgcagacgggatcgatttcatgattttttttgtttcgttgcatagggtttgg
tttgcccttttcctttatttcaatatatgccgtgcacttgtttgtcgggtcatcttttcatgcttttttttgtcttggtt
gtgatgatgtggtctggttgggcggtcgttctagatcggagtagaattctgtttcaaactacctggtggatttattaatt
ttggatctgtatgtgtgtgccatacatattcatagttacgaattgaagatgatggatggaaatatcgatctaggataggt
atacatgttgatgcgggttttactgatgcatatacagagatgctttttgttcgcttggttgtgatgatgtggtgtggttg
ggcggtcgttcattcgttctagatcggagtagaatactgtttcaaactacctggtgtatttattaattttggaactgtat
gtgtgtgtcatacatcttcatagttacgagtttaagatggatggaaatatcgatctaggataggtatacatgttgatgtg
ggttttactgatgcatatacatgatggcatatgcagcatctattcatatgctctaaccttgagtacctatctattataat
aaacaagtatgttttataattattttgatcttgatatacttggatgatggcatatgcagcagctatatgtggattttttt
agccctgccttcatacgctatttatttgcttggtactgtttcttttgtcgatgctcaccctgttgtttggtgttacttct
gcaggtcgactctagaggatccccgggtacCATGGGAAAACCTATTCCTAATCCTCTGCTGGGCCTGGATTCTACCgccc
caaagaagaagcgcaaggtcGGTATTCATGGAGTTCCTGCTGCGgacaagaagtactccatcggcctcgccatcggcacc
aattctgttggctgggccgtgatcaccgacgagtacaaggtgccgtccaagaagttcaaggtcctcggcaacaccgaccg
ccactccatcaagaagaatctcatcggcgccctgctgttcgactctggcgagacagccgaggctacaaggctcaagagga
ccgctagacgcaggtacaccaggcgcaagaaccgcatctgctacctccaagagatcttctccaacgagatggccaaggtg
gacgacagcttcttccacaggctcgaggagagcttcctcgtcgaggaggacaagaagcacgagcgccatccgatcttcgg
caacatcgtggatgaggtggcctaccacgagaagtacccgaccatctaccacctccgcaagaagctcgtcgactccaccg
ataaggccgacctcaggctcatctacctcgccctcgcccacatgatcaagttcaggggccacttcctcatcgagggcgac
ctcaacccggacaactccgatgtggacaagctgttcatccagctcgtgcagacctacaaccagctgttcgaggagaaccc
gatcaacgcctctggcgttgacgccaaggctattctctctgccaggctctctaagtcccgcaggctcgagaatctgatcg
cccaacttccgggcgagaagaagaatggcctcttcggcaacctgatcgccctctctcttggcctcaccccgaacttcaag
tccaacttcgacctcgccgaggacgccaagctccagctttccaaggacacctacgacgacgacctcgacaatctcctcgc
ccagattggcgatcagtacgccgatctgttcctcgccgccaagaatctctccgacgccatcctcctcagcgacatcctca
gggtgaacaccgagatcaccaaggccccactctccgcctccatgatcaagaggtacgacgagcaccaccaggacctcaca
ctcctcaaggccctcgtgagacagcagctcccagagaagtacaaggagatcttcttcgaccagtccaagaacggctacgc
cggctacatcgatggcggcgcttctcaagaggagttctacaagttcatcaagccgatcctcgagaagatggacggcaccg
aggagctgctcgtgaagctcaatagagaggacctcctccgcaagcagcgcaccttcgataatggctccatcccgcaccag
atccacctcggcgagcttcatgctatcctccgcaggcaagaggacttctacccgttcctcaaggacaaccgcgagaagat
tgagaagatcctcaccttccgcatcccgtactacgtgggcccgctcgccaggggcaactccaggttcgcctggatgacca
gaaagtccgaggagacaatcacccccctggaacttcgaggaggtggtggataagggcgcctctgcccagtctttcatcgag
cgcatgaccaacttcgacaagaacctcccgaacgagaaggtgctcccgaagcactcactcctctacgagtacttcaccgt
gtacaacgagctgaccaaggtgaagtacgtgaccgaggggatgaggaagccagctttccttagcggcgagcaaaagaagg
ccatcgtcgacctgctgttcaagaccaaccgcaaggtgaccgtgaagcagctcaaggaggactacttcaagaaaatcgag
tgcttcgactccgtcgagatctccggcgtcgaggataggttcaatgcctccctcgggacctaccacgacctcctcaagat
tatcaaggacaaggacttcctcgacaacgaggagaacgaggacatcctcgaggacatcgtgctcaccctcaccctcttcg
aggaccgcgagatgatcgaggagcgcctcaagacatacgcccacctcttcgacgacaaggtgatgaagcagctgaagcgc
aggcgctataccggctggggcaggctctctaggaagctcatcaacggcatccgcgacaagcagtccggcaagacgatcct
cgacttcctcaagtccgacggcttcgccaaccgcaacttcatgcagctcatccacgacgactccctcaccttcaaggagg
acatccaaaaggcccaggtgtccggccaaggcgattccctccatgagcatatcgccaatctcgccggctccccggctatc
aagaagggcattctccagaccgtgaaggtggtggacgagctggtgaaggtgatgggcaggcacaagccagagaacatcgt
gatcgagatggcccgcgagaaccagaccacacagaagggccaaaagaactcccgcgagcgcatgaagaggatcgaggagg
gcattaaggagctgggctcccagatcctcaaggagcacccagtcgagaacacccagctccagaacgagaagctctacctc
tactacctccagaacggccgcgacatgtacgtggaccaagagctggacatcaaccgcctctccgactacgacgtggacgc
tattgtgccgcagtccttcctgaaggacgactccatcgacaacaaggtgctcacccgctccgacaagaacaggggcaagt
ccgataacgtgccgtccgaagaggtcgtcaagaagatgaagaactactggcgccagctcctcaacgccaagctcatcacc
cagaggaagttcgacaacctcaccaaggccgagagaggcggcctttccgagcttgataaggccggcttcatcaagcgcca
gctcgtcgagacacgccagatcacaaagcacgtggcccagatcctcgactcccgcatgaacaccaagtacgacgagaacg

acaagctcatccgcgaggtgaaggtcatcaccctcaagtccaagctcgtgtccgacttccgcaaggacttccagttctac
aaggtgcgcgagatcaacaactaccaccacgcccacgacgcctacctcaatgccgtggtgggcacagccctcatcaagaa
gtacccaaagctcgagtccgagttcgtgtacggcgactacaaggtgtacgacgtgcgcaagatgatcgccaagtccgagc
aagagatcggcaaggcgaccgccaagtacttcttctactccaacatcatgaatttcttcaagaccgagatcacgctcgcc
aacggcgagattaggaagaggccgctcatcgagacaaacggcgagacaggcgagatcgtgtgggacaagggcagggattt
cgccacagtgcgcaaggtgctctccatgccgcaagtgaacatcgtgaagaagaccgaggttcagaccggcggcttctcca
aggagtccatcctcccaaagcgcaactccgacaagctgatcgcccgcaagaaggactgggacccgaagaagtatggcggc
ttcgattctccgaccgtggcctactctgtgctcgtggttgccaaggtcgagaagggcaagagcaagaagctcaagtccgt
caaggagctgctgggcatcacgatcatggagcgcagcagcttcgagaagaacccaatcgacttcctcgaggccaagggct
acaaggaggtgaagaaggacctcatcatcaagctcccgaagtacagcctcttcgagcttgagaacggccgcaagagaatg
ctcgcctctgctggcgagcttcagaagggcaacgagcttgctctcccgtccaagtacgtgaacttcctctacctcgcctc
ccactacgagaagctcaagggctccccagaggacaacgagcaaaagcagctgttcgtcgagcagcacaagcactacctcg
acgagatcatcgagcagatctccgagttctccaagcgcgtgatcctcgccgatgccaacctcgataaggtgctcagcgcc
tacaacaagcaccgcgataagccaattcgcgagcaggccgagaacatcatccacctcttcaccctcaccaacctcggcgc
tccagccgccttcaagtacttcgacaccaccatcgaccgcaagcgctacacctctaccaaggaggttctcgacgccaccc
tcatccaccagtctatcacaggcctctacgagacacgcatcgacctctcacaactcggcggcgatCCGGAATTTATGGCC
ATGGAGGCCCCGGGGATCCGTatggcggggagggagaagaggcggcgggtggcggcgctcgacggcgaagagcggcggcg
gcggcaggaggaagcggcgaccctcctccacaggatcagaggtgcactccccctccctctcgtgatcaatcgcttaaaaa
acttctgaatccttcatcaaccgaagtaaacacgcgtccctcgtttttttgcgcagggttggtgcgctgggtcgtcgccga
ggtcgccgccggccgctccccgaccgtcgcgctccaccgctaccagaactactgctcctccgcctccgccgccgccgcgt
ccccatggtcggtttccgcgtctcgccctaccctaccatctcatcggccgtttcaaattctcaccccgctgtatgcgctt
gcagcgcctgtagctacgacgtccccgtgggcacagatgtactctcgctcctccaccggggctcccacgcctcccggctc
agtacgcgtcgttgatttttgtctcaacctgcgtatctacgatcatgttaatgttaatcagtttgggatgttttaatcga
tgtgcagatgtgctcctcagggttcttctcgtcgtgcagcaactcctccaacagaacaagcactgctccaagagggacat
ctactacatgtaccctccatattccaaggtctgcataggttccatgcctccgcttctgcttttgtttcctgctgtgtgt
gacaattggaattctgacacctgtttttgtctaaatactccagaacaagcggttgttgaccgtgcaatcaatgatatatg
cgtactcttcaagtgcagccggcacaatctcaatgtggtatgtaccgcgtaatctgtgttattttccttcagaatcttca
gttttgcttctagaagttgcgtgtgcatcacataataagattccactttccagctgctacatcctttgtgcccacatttc
tgtcattaatcacctggagcctttttttttctcaatgctgttttacatcttgaattcatgtgtatgtactgtcataactga
ctggaatgtgtgctaccttggctgcttgcaggttcctgtggcaaaagggtaggaactctgtcaactatcatagagttgga
gtgtctgaagatatgtttaactcattctactgtcctattgattgtccatgccaatacttaataagataacttctaacccc
ttatcttccaagcacttggtctgcttaaaacaaaatctggatgtaaatagtgagatgcttctcttttagcaagggcttat
agagcactgtgaagctaccattacagtgctatgctcatatattcttcacctattcatttcccctgttaaaaactcatcaa
tgtctttattattccagcttggtgatgggctggattagatttttagagggagaaaaggaagtctattgtgtaacgaatgt
caatgctgtaagttctatgagtttcctttaatcactgtaatatgtttctttctttttattgatatggtttgatttttgcc
ttcttcaaatgcaggcattctctatcccagttagcatcgaagcaataaaaggttgttcagaaatttaaagatctaaaatt
cagtatttatgttactgttaatctgcactgaaaataattcctttacaatttgctgacatggattgcgtgaggggttggac
cataatctcaattattcagaattagccaccatcctttgttttgagagaatccactttgatatattctgaatcgaatagct
aggattgatctcttagcaaggtgctgttcattagactaaagaataaagtcagtgcttagtatctttacccgtcttgattt
aagatatgccagaaaaagactggacaacaagtgaacccacagtttattcttcatttccttacaaatatgcggttgatta
tacttatatctttactcactttatgttacagatttcctctcttgttatctgtgaacttcagtgttatccctgatgcctg
aattatatctcaaggaaattaagctatcttaggttttctcttgtagatgttgttagtgttgcggactacatacttattgt
cgagaaggagacaggtatgaggcttaaagattcctcatgtgcatgtgatacttgtactcttccagttccagccttcctac
agttcgcttctgtactctccttgcagtgtttcaacgtttggctaatgacaagttctgtgaaaggaatcgctgcattgtga
ttacagtacttactaactgctgaatctggtgttatacacaaaatatcttcttgcatttattcttatctagtttgatgttg
ttctgtctgctcagggaagaggctacccagatattccaacaagaaggcatatactctttgttatactttctcttttatt
tttctgaattgatatttcaaatgtaaatactttatttttccagattcttgcgctaccttgttgaacagctgcatttgcca
gtttactgtttggtggatgcagacccttatggtttcgacattctggctacctacaaatttggttcactggtgggtcatga
tttatctaattagctgtttgctttgtatttgctattatgcactatctgagcatacctgttaattaaaaatcacatataac
cactttatgcaaatgactactacactttccttatcagtagctcccaatagctggattgacctttatgcattacattttg
tccagtatcaaggcacatttgaatcaaacagtatgccttcagtttgtttggcagctctaagcccttttagcatgtcttgt
tttcacgatattctgtcccattttgctggtagatttggaccttgcaagtttatttaatttccttgaaagcttggtgtat
ttatttgtgttaactaactaccttctgcaatgcgcttgtgtaattctcattctatttaagcaattggcatacgatgcaaa
tttcctgcgtgtgcctgatattcggtggcttggggtcttcacatctgattttgaggattatcgccttccagactgctgcc
tacttcacttgtcgtctgaaggtgactaaactgagggatataaatgactgacatttctacaacttttttcttggaacttat
aagtaccttgtttgttcttattcaattgtcaacctcatgcttcatgtattctgttgcagacagaaggaaagctgaaggaa
ttctctcaaggtgttacttgcacagggaagccccacaatggaggtaaggttgtacttttcgtacccgtattacaagcaat
tcatgattatgatgggagtttacaagttagagctcggacttagctagtggaacatttctttgcttttgttttaacagtag
ttaaagtcatttttttgaagactttcttatgaaaaacaggccagcttactaataaaagctgtactcctaacttagtgcct
tcccttcagtctcatttccttagtttgaaaaactccactacgcccagtaggtgcaagttttggggatgaattttagtgta
ccacttgcctaggaagacttggcccattttatttccagtaactttagccctgaattttttacactgatatactttatttg
gcaggttggagttagaagccatgttgcaaaagggtgtcaaatttgagattgaggcgttatctgcatgttccatttccttt
ttatcagaagagtacattcccaagaagatcaaacaaggaagacatatatagaggcctGagctcgaatttccccgatcgtt
caaacatttggcaataaagtttcttaagattgaatcctgttgccggtcttgcgatgattatcatataatttctgttgaat
tacgttaagcatgtaataattaacatgtaatgcatgacgttatttatgagatgggtttttatgattagagtcccgcaatt
atacatttaatacgcgatagaaaacaaaatatagcgcgcaaactaggataaattatcgcgcgcggtgtcatctatgttac
tagatcgg

cDNA-sPO11-1 (used to replace the gDNA in pZmUbi1-dCas9-gSPO11-1-tNos plasmid)

atggcggggagggagaagaggcggcgggtggcggcgctcgacggcgaagagcggcggcggcggcaggaggaagcggcgac
cctcctccacaggatcagagggttggtgcgctgggtcgtcgccgaggtcgccgccggccgctccccgaccgtcgcgctcc
accgctaccagaactactgctcctccgcctccgccgccgccgcgtccccatgcgcctgtagctacgacgtccccgtgggc
acagatgtactctcgctcctccaccggggctcccacgcctcccggctcaatgtgctcctcagggttcttctcgtcgtgca
gcaactcctccaacagaacaagcactgctccaagagggacatctactacatgtacccctccatattccaagaacaagcgg
ttgttgaccgtgcaatcaatgatatatgcgtactcttcaagtgcagccggcacaatctcaatgtggttcctgtggcaaaa
ggcttggtgatgggctggattagattttagagggagaaaaggaagtctattgtgtaacgaatgtcaatgctgcattctc
tatcccagttagcatcgaagcaataaaagatgttgttagtgttgcggactacatacttattgtcgagaaggagacagtgt
ttcaacgtttggctaatgacaagttctgtgaaaggaatcgctgcattgtgattacaggaagaggctacccagatattcca
acaagaagattcttgcgctaccttgttgaacagctgcatttgccagtttactgtttggtggatgcagacccttatggttt
cgacattctggctacctacaaatttggttcactgcaattggcatacgatgcaaatttcctgcgtgtgcctgatattcggt
ggcttggggtcttcacatctgattttgaggattatcgccttccagactgctgcctacttcacttgtcgtctgaagacaga
aggaaagctgaaggaattctctcaaggtgttacttgcacagggaagccccacaatggaggttggagttagaagccatgtt
gcaaaagggtgtcaaatttgagattgaggcgttatctgcatgttccatttcctttttatcagaagagtacattcccaaga
agatcaaacaaggaagacatatatag

11 gRNA:tRNA examplified for the Chr.7 region

Legend : OsU3, tRNA, crRNA, gRNA scafflod

aaggaatctttaaacatacgaacagatcacttaaagttcttctgaagcaacttaaagttatcaggcatgcatggatcttg
gaggaatcagatgtgcagtcagggaccatagcacaagacaggcgtcttctactggtgctaccagcaaatgctggaagccg
ggaacactgggtacgttggaaaccacgtgatgtgaagaagtaagataaactgtaggagaaaagcatttcgtagtgggcca
tgaagcctttcaggacatgtattgcagtatgggccggcccattacgcaattggacgacaacaaagactagtattagtacc
acctcggctatccacatagatcaaagctgatttaaaagagttgtgcagatgatccgtggcaACAAAGCACCAGTGGtcta
gtggtagaatagtaccctgccacggtacagacccgggttcgattcccggctggtgcaTAAGCAGaagcCATGTGCTAgtt
ttagagctagaaatagcaagttaaaataaggctagtccgttatcaacttgaaaaagtggcaccgagtcggtgcaacaaag
caccagtggtctagtggtagaatagtaccctgccacggtacagacccgggttcgattcccggctggtgcaGTGCGTGTTt
ataGCAAATGgttttagagctagaaatagcaagttaaaataaggctagtccgttatcaacttgaaaaagtggcaccgagt
cggtgcaacaaagcaccagtggtctagtggtagaatagtaccctgccacggtacagacccgggttcgattcccggctggt
gcaCGTAAGTagtcGTTGATATGgttttagagctagaaatagcaagttaaaataaggctagtccgttatcaacttgaaaa
agtggcaccgagtcggtgcaacaaagcaccagtggtctagtggtagaatagtaccctgccacggtacagacccgggttcg
attcccggctggtgcaAGAGTCTatctTCTTGGGTGgttttagagctagaaatagcaagttaaaataaggctagtccgtt
atcaacttgaaaaagtggcaccgagtcggtgcaacaaagcaccagtggtctagtggtagaatagtaccctgccacggtac
agacccgggttcgattcccggctggtgcaTGACGACAgtggAAAAGCGTgttttagagctagaaatagcaagttaaaata
aggctagtccgttatcaacttgaaaaagtggcaccgagtcggtgcaacaaagcaccagtggtctagtggtagaatagtac
cctgccacggtacagacccgggttcgattcccggctggtgcaGGGTTAAaccgACATCGACCgttttagagctagaaata
gcaagttaaaataaggctagtccgttatcaacttgaaaaagtggcaccgagtcggtgcaacaaagcaccagtggtctagt
ggtagaatagtaccctgccacggtacagacccgggttcgattcccggctggtgcaCATTTGTtctcTTGGTCTATgtttt
agagctagaaatagcaagttaaaataaggctagtccgttatcaacttgaaaaagtggcaccgagtcggtgcaacaaagca
ccagtggtctagtggtagaatagtaccctgccacggtacagacccgggttcgattcccggctggtgcaAGCGGGGaggaC
ATTACACAgttttagagctagaaatagcaagttaaaataaggctagtccgttatcaacttgaaaaagtggcaccgagtcg
gtgcaacaaagcaccagtggtctagtggtagaatagtaccctgccacggtacagacccgggttcgattcccggctggtgc
aTGATGATGgactCCTAGCAAgttttagagctagaaatagcaagttaaaataaggctagtccgttatcaacttgaaaaag
tggcaccgagtcggtgcaacaaagcaccagtggtctagtggtagaatagtaccctgccacggtacagacccgggttcgat
tcccggctggtgcaGGATTCAAatacCCTTCACAgttttagagctagaaatagcaagttaaaataaggctagtccgttat
caacttgaaaaagtggcaccgagtcggtgcaacaaagcaccagtggtctagtggtagaatagtaccctgccacggtacag
acccgggttcgattcccggctggtgcaTTTGGGTttaaTCCTCCGATgttttagagctagaaatagcaagttaaaataag
gctagtccgttatcaacttgaaaaagtggcacCGAGTCGGTGCTTTTTTTTTTgttttagagctagaaatagcaagttaa
aataaggctagtccgttatcaacttgaaaaagtggcaccgagtcggtgcttttttttttaagctt
