# Supplementary material for: dCas9-SPO11-1 locally stimulates meiotic recombination in rice
Source: Front Plant Sci. 2025 May 1;16:1580225. doi: 10.3389/fpls.2025.1580225 (PMC12078263; doi:10.3389/fpls.2025.1580225)
Supplement: Supplementary file 9 [file DataSheet9.pdf]

	Interval	Method	Number of experiments	Total number of dPCR wells	Genotype	Total number of plants/nuclei	Number of recombinants	Corrected number of recombinants	Corrected recombination frenquencies (%)	Fisher Exact Test
Chr.9	**1** 9#1 #2; 9#1 #2 #4	KASP			WT	1973	6		0,15	
		dPCR	3	21	Background	8928	14			
			3	19	WT	7495	14	2,2	0,03	0,460
			3	21	9a	8549	18	4,6	0,05	
			2	14	Background	6461	7			
			3	20	WT	9403	14	3,8	0,04	0,537
			3	21	9b	8805	16	6,5	0,07	
	**2** 9#2 9#3; 9#1 9#4	KASP			WT	1973	12		0,30	
		dPCR	3	21	Background	8928	12			
			3	19	WT	7495	33	22,9	0,31	1,000
			3	21	9a	8549	37	25,5	0,30	
			2	14	Background	6461	14			
			3	20	WT	9403	44	23,6	0,25	0,343
			3	21	9b	8805	35	15,9	0,18	
	**3** 9#3 #4; 9#1 #3 #4	KASP			WT	1973	4		0,10	
		dPCR	3	21	Background	8928	11			
			3	19	WT	7495	23	13,8	0,18	0,307
			3	21	9a	8549	21	10,5	0,12	
			2	14	Background	6461	4			
			3	20	WT	9403	17	11,2	0,12	0,684
			3	21	9b	8805	18	12,5	0,14	
Chr.9	**2** 9#2 9#3	KASP			WT	1973	12		0,30	
		dPCR	3	21	Background	8928	2			
			3	19	WT	7495	20	18,3	0,24	0,614
			3	21	9a	8549	19	17,1	0,20	
			2	14	Background	6461	0			
			3	20	WT	9403	20	20,0	0,21	0,288
			3	21	9b	8805	12	12,0	0,14	
	**3** 9#3 9#4	KASP			WT	1973	4		0,10	
		dPCR	3	21	Background	8928	4			
			3	19	WT	7495	5	1,6	0,02	0,602
			3	21	9a	8549	5	1,2	0,01	
			2	14	Background	6461	1			
			3	20	WT	9403	6	4,5	0,05	0,411
			3	21	9b	8805	9	7,6	0,09	

Supplementary Table 1

Supplementary Table 1: Summary of recombinants observed in the Chr.9 target region following genotyping of F2 progeny plants and pollen nuclei by KASP and dPCR respectively.

Estimation of the recombination frequency in the target regions was carried out by Kasp using a KalingaIII/Kitaake F2 progeny of ca. 2,000 individuals. Compared with pollen, the plants analyzed with Kasp are the product of two meioses. Kasp probes were designed using the same SNP/Indel as the dPCR probes or the closest functional SNP/Indel (**Figure S1A**). Only Kasp probe 9#1 is 7.877bp and 7.896 bp further from the probe used for pollen typing in the Kitaake and KalingaIII genomes respectively. Genotyping of pollen nuclei was carried out according to the experimental flow shown in **Figure 3(A-B)**. Data from successfully genotyped pollen nuclei of biological replicates were pooled to assess the recombination frequency detected by pollen typing and corrected (**Methods**).
