# Supplementary material for: dCas9-SPO11-1 locally stimulates meiotic recombination in rice
Source: Front Plant Sci. 2025 May 1;16:1580225. doi: 10.3389/fpls.2025.1580225 (PMC12078263; doi:10.3389/fpls.2025.1580225)
Supplement: Supplementary file 10 [file DataSheet10.pdf]

(A)

Raw table name	Sample	Well	Nombre partitions valides	Nombre partitions positives	Nombre partitions négatives	KiZ1	KaZ4	KiZ3	KaZ2	KaZ4/KiZ1	KiZ3/KiZ1	KiZ3/KaZ4	KaZ2/KiZ1	KaZ2/KaZ4	KaZ2/KiZ3	KiZ3/KaZ4/KiZ1	KaZ2/KaZ4/KiZ1	KaZ2/KiZ3/KiZ1	KaZ2/KiZ3/KaZ4	KaZ2/KiZ3/KaZ4/KiZ1
Correspondences with Figures	Sample	Well	Number of valid partitions	Number of positive partitions	Number of negative partitions	Probe #1	Probe #4	Probe #3	Probe #2	Association #4#1	Association #3#1	Association #3#4	Association #2#1	Association #2#4	Association #2#3	Association #3#4#1	Association #2#4#1	Association #2#3#1	Association #2#3#4	Association #1#2#3#4

(B)

Sample	Well	Nombre partitions valides	Nombre partitions positives	Nombre partitions négatives	KiZ1	KaZ4	KiZ3	KaZ2	KaZ4/KiZ1	KiZ3/KiZ1	KiZ3/KaZ4	KaZ2/KiZ1	KaZ2/KaZ4	KaZ2/KiZ3	KiZ3/KaZ4/KiZ1	KaZ2/KaZ4/KiZ1	KaZ2/KiZ3/KiZ1	KaZ2/KiZ3/KaZ4	KaZ2/KiZ3/KaZ4/KiZ1
A- Kitaake 1000	A1	25393	271	25122	13	0	24	0	0	234	0	0	0	0	0	0	0	0	0
B- Kalinga 1000	B1	25266	362	24904	4	81	0	11	2	0	0	7	256	0	0	1	0	0	0
C- 500:500	C1	25378	351	25027	6	29	25	7	0	124	0	1	157	0	0	2	0	0	0
D- 500:500	D1	25397	351	25046	7	36	16	5	2	116	0	0	167	0	0	2	0	0	0
E- 500:500	E1	25401	386	25015	11	28	23	7	0	143	0	2	172	0	0	0	0	0	0
F- 500:500	F1	25340	389	24951	11	28	22	6	2	126	0	2	188	0	0	3	1	0	0
G- 500:500	G1	25412	378	25034	7	21	19	8	3	164	0	3	149	0	0	3	1	0	0
H- 500:500	H1	25128	382	24746	12	23	23	5	10	142	0	2	150	0	0	15	0	0	0
I- 500:500	A2	25403	374	25029	9	23	19	7	1	140	0	4	164	0	0	7	0	0	0
J- Hybride 1000	B2	25399	289	25110	10	23	13	6	0	127	0	3	105	0	0	1	1	0	0
K- Hybride 1000	C2	25367	315	25052	4	24	18	8	0	127	0	1	132	1	0	0	0	0	0
L- Hybride 1000	D2	25368	295	25073	11	14	13	6	0	114	0	1	131	1	0	1	2	1	0
M- Hybride 1000	E2	25429	305	25124	7	23	12	6	0	116	0	4	136	0	0	1	0	0	0
N- Hybride 1000	F2	25352	287	25065	2	20	13	9	1	122	0	1	117	2	0	0	0	0	0
O- Hybride 1000	G2	25228	322	24906	14	17	31	7	0	103	0	6	141	2	0	1	0	0	0
P- Hybride 1000	H2	25391	381	25010	11	20	22	6	1	168	0	4	139	1	0	9	0	0	0
Q- T478 1.2 1000	A3	25343	154	25189	3	13	10	4	2	66	0	0	56	0	0	0	0	0	0
R- T478 1.2 1000	B3	25483	185	25298	11	6	13	9	2	66	0	1	76	0	0	1	0	0	0
S- T478 1.2 1000	C3	25389	188	25201	5	8	11	5	0	80	0	1	77	0	0	1	0	0	0
T- T478 1.2 1000	D3	25438	214	25224	8	12	20	6	2	74	0	1	90	0	0	1	0	0	0
U- T478 1.2 1000	E3	25461	201	25260	11	13	18	5	0	69	0	1	82	2	0	0	0	0	0
V- T478 1.2 1000	F3	25410	192	25218	9	10	11	6	2	73	0	0	79	0	0	2	0	0	0
W- T478 1.2 1000	G3	25447	219	25228	10	14	21	5	0	78	0	1	89	0	0	1	0	0	0
H2O	H3	25239	0	25239	0	0	0	0	0	0	0	0	0	0	0	0	0	0	0

Sample	Well	Nombre partitions valides	Nombre partitions positives	Nombre partitions négatives	KiZ1	KaZ4	KiZ3	KaZ2	KaZ4/KiZ1	KiZ3/KiZ1	KiZ3/KaZ4	KaZ2/KiZ1	KaZ2/KaZ4	KaZ2/KiZ3	KiZ3/KaZ4/KiZ1	KaZ2/KaZ4/KiZ1	KaZ2/KiZ3/KiZ1	KaZ2/KiZ3/KaZ4	KaZ2/KiZ3/KaZ4/KiZ1
A- Kitaake 1000	A1	25268	230	25038	26	0	15	0	0	189	0	0	0	0	0	0	0	0	0
B- Kalinga 1000	B1	25190	446	24744	2	48	1	11	2	0	0	6	369	0	0	6	0	1	0
C- 500:500	C1	25126	397	24729	8	26	39	6	0	111	0	0	206	0	0	1	0	0	0
D- 500:500	D1	25279	346	24933	6	26	30	5	0	85	0	2	186	0	0	4	0	2	0
E- 500:500	E1	25322	391	24931	10	26	20	4	0	129	0	1	197	0	0	3	0	1	0
F- 500:500	F1	25304	395	24909	16	22	38	25	0	122	0	3	167	0	0	1	0	1	0
G- 500:500	G1	25436	405	25031	20	26	16	7	7	122	2	10	176	2	0	9	6	1	1
H- 500:500	H1	25203	406	24797	10	23	31	9	11	120	1	11	148	1	0	35	5	0	1
I- 500:500	A2	25297	405	24892	10	44	89	11	0	49	0	0	201	1	0	0	0	0	0
J- Hybride 1000	B2	25277	276	25001	4	20	14	4	3	121	0	0	109	0	1	0	0	0	0
K- Hybride 1000	C2	25201	256	24945	3	5	12	4	1	114	0	1	113	0	0	1	2	0	0
L- Hybride 1000	D2	25364	292	25072	9	13	20	3	1	114	0	1	128	0	0	0	0	3	0
M- Hybride 1000	E2	25376	213	25163	7	11	5	6	1	99	1	2	79	0	0	1	0	1	0
N- Hybride 1000	F2	25439	289	25150	8	22	16	4	1	121	0	0	115	0	0	1	0	0	1
O- Hybride 1000	G2	25148	349	24799	9	22	10	4	0	143	1	1	153	0	0	3	1	2	0
P- Hybride 1000	H2	25368	396	24972	5	16	10	6	3	174	0	8	159	1	0	7	4	2	1
Q- T478 1.2 1000	A3	25317	203	25114	8	13	17	6	1	77	0	2	75	0	0	1	0	3	0
R- T478 1.2 1000	B3	25450	234	25216	8	17	15	3	3	91	0	2	93	0	0	0	0	1	1
S- T478 1.2 1000	C3	25339	248	25091	10	12	12	8	0	102	1	5	91	2	0	3	1	1	0
T- T478 1.2 1000	D3	25438	256	25182	13	26	20	8	0	86	1	2	98	0	0	1	1	0	0
U- T478 1.2 1000	E3	25376	287	25089	8	9	29	15	1	99	0	1	119	3	0	2	0	1	0
V- T478 1.2 1000	F3	25450	282	25168	13	13	21	10	1	110	2	4	103	0	0	0	2	3	0
W- T478 1.2 1000	G3	25428	301	25127	13	15	25	9	5	112	0	5	107	2	1	3	2	2	0
H20	H3	12018	11850	168	0	0	11848	0	0	1	1	0	0	0	0	0	0	0	0

Sample	Well	Nombre partitions valides	Nombre partitions positives	Nombre partitions négatives	KiZ1	KaZ4	KiZ3	KaZ2	KaZ4/KiZ1	KiZ3/KiZ1	KiZ3/KaZ4	KaZ2/KiZ1	KaZ2/KaZ4	KaZ2/KiZ3	KiZ3/KaZ4/KiZ1	KaZ2/KaZ4/KiZ1	KaZ2/KiZ3/KiZ1	KaZ2/KiZ3/KaZ4	KaZ2/KiZ3/KaZ4/KiZ1
A- Kitaake 1000	A1	25138	305	24833	25	0	16	0	0	264	0	0	0	0	0	0	0	0	0
B- Kalinga 1000	B1	25237	210	25027	3	52	0	10	1	0	0	2	141	0	0	1	0	0	0
C- 500500	C1	25207	303	24904	5	23	15	4	1	153	0	0	102	0	0	0	0	0	0
D- 500500	D1	25428	312	25116	15	36	19	9	1	133	0	0	98	0	0	1	0	0	0
E- 500500	E1	25200	337	24863	8	23	12	7	0	156	0	0	128	0	0	2	1	0	0
F- 500500	F1	25399	344	25055	8	24	8	5	3	144	0	2	147	1	0	1	1	0	0
G- 500500	G1	25405	344	25061	10	23	14	7	2	151	0	3	131	0	0	2	1	0	0
H- 500500	H1	25354	326	25028	10	22	9	5	5	134	0	12	112	0	0	16	1	0	0
H20	H3	25267	2	25265	1	1	0	0	0	0	0	0	0	0	0	0	0	0	0
I- 500500	A2	25244	338	24906	13	112	25	6	1	142	0	0	38	0	0	1	0	0	0
J- Hybride 1000	B2	25403	220	25183	6	15	9	3	0	103	0	1	80	0	0	1	1	1	0
K- Hybride 1000	C2	25223	240	24983	7	21	13	4	0	95	1	1	93	0	1	4	0	0	0
L- Hybride 1000	D2	25437	202	25235	4	11	6	6	1	92	0	0	82	0	0	0	0	0	0
M- Hybride 1000	E2	25440	222	25218	7	15	4	6	1	88	0	0	98	1	0	2	0	0	0
N- Hybride 1000	F2	25415	249	25166	9	14	7	3	1	107	0	0	103	0	0	3	1	1	0
O- Hybride 1000	G2	24875	297	24578	6	25	7	10	1	125	0	3	117	1	1	1	0	0	0
P- Hybride 1000	H2	25323	325	24998	12	18	7	5	5	136	0	2	126	0	0	12	1	1	0
Q- T478 1.2 1000	A3	25310	140	25170	8	12	7	4	0	58	0	0	50	0	0	1	0	0	0
R- T478 1.2 1000	B3	25423	182	25241	8	15	6	7	0	66	0	1	79	0	0	0	0	0	0
S- T478 1.2 1000	C3	25370	201	25169	13	14	11	10	0	68	0	2	76	0	0	5	1	0	1
T- T478 1.2 1000	D3	25426	177	25249	7	7	7	10	2	72	0	0	70	1	0	1	0	0	0
U- T478 1.2 1000	E3	25462	214	25248	11	16	15	12	1	80	0	0	75	0	1	3	0	0	0
V- T478 1.2 1000	F3	25359	204	25155	12	14	36	7	0	50	0	2	81	2	0	0	0	0	0
W- T478 1.2 1000	G3	25413	246	25167	11	16	21	7	1	101	0	4	83	0	1	1	0	0	0

Sample	Well	Nombre partitions valides	Nombre partitions positives	Nombre partitions négatives	KiZ1	KaZ4	KiZ3	KaZ2	KaZ4/KiZ1	KiZ3/KiZ1	KiZ3/KaZ4	KaZ2/KiZ1	KaZ2/KaZ4	KaZ2/KiZ3	KiZ3/KaZ4/KiZ1	KaZ2/KaZ4/KiZ1	KaZ2/KiZ3/KiZ1	KaZ2/KiZ3/KaZ4	KaZ2/KiZ3/KaZ4/KiZ1
	A2	8259	0	8259	0	0	0	0	0	0	0	0	0	0	0	0	0	0	0
A- Kitaake 1000	A1	25292	215	25077	9	1	13	0	0	192	0	0	0	0	0	0	0	0	0
B- Kalinga 1000	B1	25236	263	24973	0	49	1	16	0	0	0	0	196	0	0	1	0	0	0
C- 500500	C1	25354	243	25111	5	29	19	5	0	83	0	0	102	0	0	0	0	0	0
D- 500500	D1	25365	289	25076	9	25	20	4	0	124	1	3	102	0	0	0	0	0	1
E- 500500	E1	25367	296	25071	10	22	32	9	1	101	1	1	118	0	0	0	0	1	0
F- 500500	F1	25334	286	25048	8	27	21	7	0	103	0	0	119	0	0	0	1	0	0
G- 500500	G1	25409	300	25109	16	29	21	7	2	100	0	0	123	0	0	1	0	1	0
H- 500500	H1	24404	296	24108	13	27	7	4	5	114	1	3	115	0	0	7	0	0	0
H2O	H3	25230	1	25229	0	0	0	1	0	0	0	0	0	0	0	0	0	0	0
J- Hybride 1000	B2	25384	302	25082	7	13	13	4	1	119	0	0	142	1	0	0	0	1	1
K- Hybride 1000	C2	25287	283	25004	9	11	19	5	0	108	0	0	129	0	0	1	1	0	0
L- Hybride 1000	D2	25453	234	25219	5	13	13	6	0	108	0	1	88	0	0	0	0	0	0
M- Hybride 1000	E2	25406	257	25149	10	23	16	8	1	96	0	1	102	0	0	0	0	0	0
N- Hybride 1000	F2	25450	290	25160	10	21	9	9	0	120	0	3	116	0	0	0	0	2	0
O- Hybride 1000	G2	25076	314	24762	14	25	19	17	1	107	0	3	126	1	0	0	0	0	1
P- Hybride 1000	H2	25380	347	25033	10	30	13	12	1	144	1	8	120	0	1	4	3	0	0
Q- T478 1.2 1000	A3	25350	252	25098	12	17	15	10	0	88	0	1	102	2	0	4	0	1	0
R- T478 1.2 1000	B3	25468	245	25223	14	18	14	7	1	105	0	3	81	0	0	1	0	1	0
S- T478 1.2 1000	C3	25400	243	25157	21	14	21	13	1	87	1	2	80	0	0	1	2	0	0
T- T478 1.2 1000	D3	25469	274	25195	15	14	24	18	1	94	3	1	101	2	0	0	1	0	0
U- T478 1.2 1000	E3	25432	268	25164	18	14	37	16	1	74	1	1	105	0	0	0	1	0	0
V- T478 1.2 1000	F3	25435	299	25136	22	16	20	14	2	113	1	2	107	1	0	0	0	1	0
W- T478 1.2 1000	G3	25308	308	25000	22	31	17	18	0	105	0	5	105	0	0	3	2	0	0

Sample	Well	Nombre partitions valides	Nombre partitions positives	Nombre partitions négatives	KiZ1	KaZ4	KiZ3	KaZ2	KaZ4/KiZ1	KiZ3/KiZ1	KiZ3/KaZ4	KaZ2/KiZ1	KaZ2/KaZ4	KaZ2/KiZ3	KiZ3/KaZ4/KiZ1	KaZ2/KaZ4/KiZ1	KaZ2/KiZ3/KiZ1	KaZ2/KiZ3/KaZ4	KaZ2/KiZ3/KaZ4/KiZ1
A- Kitaake 1000	A1	25266	226	25040	14	0	15	0	0	197	0	0	0	0	0	0	0	0	0
B- Kalinga 1000	B1	25091	333	24758	1	61	0	9	3	0	0	8	247	0	0	4	0	0	0
C- 500500	C1	25087	298	24789	10	20	22	5	0	120	0	0	121	0	0	0	0	0	0
D- 500500	D1	25363	318	25045	21	26	29	8	0	81	0	0	153	0	0	0	0	0	0
E- 500500	E1	25393	312	25081	13	26	27	8	1	107	0	0	130	0	0	0	0	0	0
F- 500500	F1	25150	282	24868	10	33	16	7	0	93	0	0	123	0	0	0	0	0	0
G- 500500	G1	25328	269	25059	13	26	16	6	0	75	0	0	132	0	0	0	0	1	0
H- 500500	H1	25125	309	24816	19	30	13	8	1	91	0	0	141	1	0	5	0	0	0
H20	H3	25290	1	25289	1	0	0	0	0	0	0	0	0	0	0	0	0	0	0
I- 500500	A2	25355	280	25075	9	31	79	5	1	32	0	0	122	0	0	1	0	0	0
J- Hybride 1000	B2	25338	186	25152	7	19	17	8	2	63	0	0	70	0	0	0	0	0	0
K- Hybride 1000	C2	25100	215	24885	13	26	20	16	0	66	0	1	72	1	0	0	0	0	0
L- Hybride 1000	D2	25417	212	25205	36	22	9	9	0	60	0	2	74	0	0	0	0	0	0
M- Hybride 1000	E2	25425	191	25234	13	20	24	8	0	65	0	0	61	0	0	0	0	0	0
N- Hybride 1000	F2	25310	209	25101	6	18	26	4	0	67	0	0	87	1	0	0	0	0	0
O- Hybride 1000	G2	25180	251	24929	14	15	59	18	0	66	1	1	75	0	0	0	1	1	0
P- Hybride 1000	H2	25321	268	25053	20	29	71	19	0	43	0	3	82	1	0	0	0	0	0
Q- T478 1.2 1000	A3	25366	115	25251	6	10	27	5	0	28	1	0	37	0	0	1	0	0	0
R- T478 1.2 1000	B3	25435	121	25314	7	18	12	6	0	46	0	0	32	0	0	0	0	0	0
S- T478 1.2 1000	C3	25359	98	25261	16	9	6	5	0	21	0	0	41	0	0	0	0	0	0
T- T478 1.2 1000	D3	25372	128	25244	5	32	35	3	0	20	0	0	33	0	0	0	0	0	0
U- T478 1.2 1000	E3	25299	151	25148	9	34	9	4	2	56	0	0	36	0	0	1	0	0	0
V- T478 1.2 1000	F3	25398	168	25230	7	14	52	2	0	29	0	0	63	1	0	0	0	0	0
W- T478 1.2 1000	G3	25373	166	25207	8	13	21	6	0	46	0	0	72	0	0	0	0	0	0

Sample	Well	Nombre partitions valides	Nombre partitions positives	Nombre partitions négatives	KiZ1	KaZ4	KiZ3	KaZ2	KaZ4/KiZ1	KiZ3/KiZ1	KiZ3/KaZ4	KaZ2/KiZ1	KaZ2/KaZ4	KaZ2/KiZ3	KiZ3/KaZ4/KiZ1	KaZ2/KaZ4/KiZ1	KaZ2/KiZ3/KiZ1	KaZ2/KiZ3/KaZ4	KaZ2/KiZ3/KaZ4/KiZ1
A- Kitaake 1000	A1	25257	330	24927	80	4	18	0	0	216	0	0	0	0	0	0	12	0	0
B- Kalinga 1000	B1	25181	330	24851	3	83	0	8	13	0	0	7	184	0	0	32	0	0	0
C- 500500	C1	25241	210	25031	22	23	12	7	0	50	0	0	95	1	0	0	0	0	0
D- 500500	D1	25370	257	25113	11	35	11	7	0	106	0	1	86	0	0	0	0	0	0
E- 500500	E1	25323	271	25052	10	34	59	13	0	64	0	0	91	0	0	0	0	0	0
F- 500500	F1	25253	276	24977	10	50	41	9	0	81	0	0	85	0	0	0	0	0	0
G- 500500	G1	25415	275	25140	16	37	17	8	0	77	0	2	115	0	1	0	2	0	0
H- 500500	H1	25094	295	24799	7	70	39	9	0	90	0	0	78	1	0	0	1	0	0
H20	H3	24881	0	24881	0	0	0	0	0	0	0	0	0	0	0	0	0	0	0
I- 500500	A2	25295	357	24938	7	40	62	19	0	97	0	0	123	2	0	3	4	0	0
J- Hybride 1000	B2	25365	266	25099	9	56	30	8	0	95	0	2	65	0	0	0	1	0	0
K- Hybride 1000	C2	25148	215	24933	5	23	65	14	0	55	0	0	53	0	0	0	0	0	0
L- Hybride 1000	D2	25436	250	25186	6	28	42	8	0	92	0	1	71	1	0	0	0	1	0
M- Hybride 1000	E2	25410	275	25135	5	78	47	7	1	82	0	0	55	0	0	0	0	0	0
N- Hybride 1000	F2	25428	274	25154	13	34	51	13	0	68	0	0	92	1	0	1	1	0	0
O- Hybride 1000	G2	25164	282	24882	16	35	28	11	0	105	0	0	86	1	0	0	0	0	0
P- Hybride 1000	H2	25260	296	24964	7	78	43	7	2	83	1	2	73	0	0	0	0	0	0
Q- T478 1.2 1000	A3	25356	213	25143	16	19	17	15	1	59	0	0	83	0	0	2	1	0	0
R- T478 1.2 1000	B3	25429	290	25139	10	36	92	6	0	37	0	0	106	1	0	0	2	0	0
S- T478 1.2 1000	C3	25283	243	25040	24	54	19	6	0	82	0	0	57	0	0	1	0	0	0
T- T478 1.2 1000	D3	25399	233	25166	10	52	23	7	1	79	0	0	59	2	0	0	0	0	0
U- T478 1.2 1000	E3	25353	288	25065	8	19	103	12	0	17	0	0	127	1	0	0	1	0	0
V- T478 1.2 1000	F3	24484	200	24284	10	34	19	5	0	88	0	0	42	1	0	1	0	0	0
W- T478 1.2 1000	G3	25365	274	25091	14	18	36	18	0	96	1	0	89	0	0	0	2	0	0

Supplementary Table 2: dPCR results and correspondences for Chr.7 plants (1/4).
Meaning of the columns for all the following raw tables and correspondences with the figures (**A**).
Table of dPCR results for Plant 7a : T478 1.2 (**B**).

Sample	Well	Partitions valides	Positives	Négatives	KiZ1	KaZ4	KiZ3	KaZ2	KaZ4/KiZ1	KiZ3/KiZ1	KiZ3/KaZ4	KaZ2/KiZ1	KaZ2/KaZ4	KaZ2/KiZ3	KiZ3/KaZ4/KiZ1	KaZ2/KaZ4/KiZ1	KaZ2/KiZ3/KiZ1	KaZ2/KiZ3/KaZ4	KaZ2/KiZ3/KaZ4/KiZ1
A- Kitaake 1000	A1	25384	451	24933	34	0	57	0	0	360	0	0	0	0	0	0	0	0	0
B- Kalinga 1000	B1	25243	305	24938	0	64	0	11	0	0	0	0	230	0	0	0	0	0	0
C- 500500	C1	25327	446	24881	8	50	208	10	0	2	1	0	165	2	0	0	0	0	0
D- 500500	D1	24729	337	24392	7	36	54	6	0	107	0	0	127	0	0	0	0	0	0
E- Hybride 1000	E1	25355	328	25027	24	35	49	23	0	72	1	2	121	1	0	0	0	0	0
F- Hybride 1000	F1	25226	305	24921	24	35	49	13	0	73	0	2	107	2	0	0	0	0	0
G- T478 11.1 1000	G1	25202	330	24872	16	20	40	17	1	121	0	4	109	0	0	2	0	0	0
H- T478 11.1 1000	H1	25264	256	25008	6	17	39	15	0	86	0	0	92	1	0	0	0	0	0
H2O	H3	25365	0	25365	0	0	0	0	0	0	0	0	0	0	0	0	0	0	0
I- T478 11.1 1000	A2	25270	272	24998	28	18	22	6	4	89	0	3	100	0	0	2	0	0	0
J- T478 11.1 1000	B2	25416	259	25157	31	19	22	8	1	76	0	1	99	0	0	2	0	0	0
K- T478 11.1 1000	C2	25375	258	25117	10	17	20	29	0	76	0	0	103	2	0	1	0	0	0
L- T478 11.1 1000	D2	24102	232	23870	8	19	11	6	2	85	0	0	101	0	0	0	0	0	0
M- T478 11.1 1000	E2	25385	269	25116	3	37	123	17	0	0	0	0	89	0	0	0	0	0	0
N- T478 11.1 1000	F2	25308	266	25042	5	27	51	13	0	64	0	0	106	0	0	0	0	0	0
O- T478 11.1 1000	G2	25312	255	25057	10	30	27	8	0	63	1	0	114	1	0	0	0	1	0
P- T478 11.1 1000	H2	25403	332	25071	10	40	59	11	1	107	0	1	99	3	0	0	1	0	0
Q- T478 11.1 1000	A3	25328	264	25064	27	31	36	11	0	71	0	0	87	0	0	1	0	0	0
R- T478 11.1 1000	B3	25492	222	25270	13	17	26	17	0	68	0	1	80	0	0	0	0	0	0
S- T478 11.1 1000	C3	25448	239	25209	18	23	35	6	1	66	0	0	89	0	0	1	0	0	0
T- T478 11.1 1000	D3	25322	237	25085	14	14	56	15	0	52	0	0	86	0	0	0	0	0	0
U- T478 11.1 1000	E3	25466	245	25221	14	19	26	7	0	91	1	2	84	1	0	0	0	0	0
V- T478 11.1 1000	F3	25488	239	25249	12	14	41	16	0	70	0	1	85	0	0	0	0	0	0
W- T478 11.1 1000	G3	25356	249	25107	8	18	32	16	0	85	0	0	90	0	0	0	0	0	0

Sample	Well	Partitions valides	Positives	Négatives	KiZ1	KaZ4	KiZ3	KaZ2	KaZ4/KiZ1	KiZ3/KiZ1	KiZ3/KaZ4	KaZ2/KiZ1	KaZ2/KaZ4	KaZ2/KiZ3	KiZ3/KaZ4/KiZ1	KaZ2/KaZ4/KiZ1	KaZ2/KiZ3/KiZ1	KaZ2/KiZ3/KaZ4	KaZ2/KiZ3/KaZ4/KiZ1
A- Kitaake 1000	A1	25371	378	24993	31	0	33	0	0	314	0	0	0	0	0	0	0	0	0
B- Kalinga 1000	B1	25270	370	24900	1	50	1	20	0	0	0	0	298	0	0	0	0	0	0
C- 500500	C1	25346	361	24985	20	28	17	9	2	139	0	0	144	0	0	2	0	0	0
D- 500500	D1	25194	370	24824	12	20	98	14	0	77	0	0	148	1	0	0	0	0	0
E- Hybride 1000	E1	25379	396	24983	24	23	23	16	0	146	0	1	161	1	0	1	0	0	0
F- Hybride 1000	F1	25260	413	24847	18	47	23	12	0	149	0	4	153	2	1	3	1	0	0
G- T478 11.1 1000	G1	25405	181	25224	8	13	20	6	0	64	0	1	69	0	0	0	0	0	0
H- T478 11.1 1000	H1	25254	216	25038	9	28	15	6	0	73	0	0	81	3	0	0	1	0	0
H2O	H3	25336	1	25335	1	0	0	0	0	0	0	0	0	0	0	0	0	0	0
I- T478 11.1 1000	A2	25220	207	25013	14	14	13	8	1	71	1	1	81	2	0	1	0	0	0
J- T478 11.1 1000	B2	25437	208	25229	10	22	19	3	0	79	0	1	71	0	0	3	0	0	0
K- T478 11.1 1000	C2	25120	177	24943	4	15	13	5	0	69	0	0	71	0	0	0	0	0	0
L- T478 11.1 1000	D2	25435	165	25270	6	17	12	7	0	62	1	1	58	1	0	0	0	0	0
M- T478 11.1 1000	E2	25471	180	25291	11	11	15	5	0	61	0	0	76	1	0	0	0	0	0
N- T478 11.1 1000	F2	25425	211	25214	8	12	23	12	0	80	0	0	76	0	0	0	0	0	0
O- T478 11.1 1000	G2	25254	223	25031	14	22	29	5	0	74	0	3	76	0	0	0	0	0	0
P- T478 11.1 1000	H2	25404	261	25143	9	24	14	9	1	109	0	0	92	2	0	1	0	0	0
Q- T478 11.1 1000	A3	25373	220	25153	7	10	22	9	0	93	0	0	78	1	0	0	0	0	0
R- T478 11.1 1000	B3	25471	212	25259	11	17	20	6	1	77	0	0	76	1	0	3	0	0	0
S- T478 11.1 1000	C3	25306	212	25094	12	17	15	8	1	82	0	1	74	0	0	1	0	1	0
T- T478 11.1 1000	D3	25439	226	25213	11	23	23	1	0	78	0	0	88	2	0	0	0	0	0
U- T478 11.1 1000	E3	25464	247	25217	9	19	26	5	0	84	0	0	102	2	0	0	0	0	0
V- T478 11.1 1000	F3	25439	220	25219	9	24	8	9	1	82	0	0	85	2	0	0	0	0	0
W- T478 11.1 1000	G3	25413	256	25157	12	21	14	8	0	98	0	0	102	0	0	1	0	0	0

Sample	Well	Partitions valides	Positives	Négatives	KiZ1	KaZ4	KiZ3	KaZ2	KaZ4/KiZ1	KiZ3/KiZ1	KiZ3/KaZ4	KaZ2/KiZ1	KaZ2/KaZ4	KaZ2/KiZ3	KiZ3/KaZ4/KiZ1	KaZ2/KaZ4/KiZ1	KaZ2/KiZ3/KiZ1	KaZ2/KiZ3/KaZ4	KaZ2/KiZ3/KaZ4/KiZ1
A- Kitaake	A1	25371	428	24943	26	0	67	0	0	335	0	0	0	0	0	0	0	0	0
B- Kitaake	B1	25173	347	24826	0	52	0	12	0	0	0	3	278	0	0	2	0	0	0
C- 500-500	C1	25146	347	24799	14	22	17	14	0	148	0	0	131	1	0	0	0	0	0
D- 500-500	D1	25437	336	25101	14	36	35	8	0	135	0	0	107	1	0	0	0	0	0
E- Hybride	E1	25369	383	24986	14	32	11	12	0	154	1	3	153	2	0	1	0	0	0
F- Hybride	F1	25298	373	24925	6	37	42	29	0	135	0	0	124	0	0	0	0	0	0
G- T478 11.1 1000	G1	25350	223	25127	5	22	33	8	0	80	0	0	75	0	0	0	0	0	0
H- T478 11.1 1000	H1	25053	260	24793	14	34	58	9	1	57	0	1	86	0	0	0	0	0	0
H2O	H3	25318	0	25318	0	0	0	0	0	0	0	0	0	0	0	0	0	0	0
I- T478 11.1 1000	A2	25317	227	25090	9	30	27	4	0	72	0	0	83	1	0	1	0	0	0
J- T478 11.1 1000	B2	25338	205	25133	4	24	21	8	0	83	1	0	63	1	0	0	0	0	0
K- T478 11.1 1000	C2	25157	231	24926	5	18	13	8	0	95	0	0	90	0	1	0	1	0	0
L- T478 11.1 1000	D2	25449	224	25225	11	22	17	6	0	71	0	1	95	0	0	1	0	0	0
M- T478 11.1 1000	E2	25435	204	25231	10	18	14	9	0	86	0	0	65	1	0	1	0	0	0
N- T478 11.1 1000	F2	25436	226	25210	8	21	22	7	2	74	0	0	92	0	0	0	0	0	0
O- T478 11.1 1000	G2	25163	235	24928	11	16	19	9	2	104	0	0	72	1	0	1	0	0	0
P- T478 11.1 1000	H2	25303	134	25169	2	22	12	7	1	51	0	1	38	0	0	0	0	0	0
Q- T478 11.1 1000	A3	25200	258	24942	7	31	24	9	0	95	0	1	91	0	0	0	0	0	0
R- T478 11.1 1000	B3	25461	250	25211	10	16	25	16	0	81	0	0	100	1	0	1	0	0	0
S- T478 11.1 1000	C3	25411	271	25140	9	18	18	15	1	112	0	1	96	0	0	1	0	0	0
T- T478 11.1 1000	D3	25451	239	25212	9	19	14	11	0	100	0	0	86	0	0	0	0	0	0
U- T478 11.1 1000	E3	25410	244	25166	0	24	24	5	0	94	0	0	97	0	0	0	0	0	0
V- T478 11.1 1000	F3	25451	243	25208	10	26	31	9	0	70	0	0	95	2	0	0	0	0	0
W- T478 11.1 1000	G3	25357	262	25095	16	23	19	7	0	91	0	2	103	0	0	1	0	0	0

Sample	Well	Nombre partitions valides	Nombre partitions positives	Nombre partitions négatives	KiZ1	KaZ4	KiZ3	KaZ2	KaZ4/KiZ1	KiZ3/KiZ1	KiZ3/KaZ4	KaZ2/KiZ1	KaZ2/KaZ4	KaZ2/KiZ3	KiZ3/KaZ4/KiZ1	KaZ2/KaZ4/KiZ1	KaZ2/KiZ3/KiZ1	KaZ2/KiZ3/KaZ4	KaZ2/KiZ3/KaZ4/KiZ1
A- Kitaake 1000	A1	25362	273	25089	21	2	38	0	0	212	0	0	0	0	0	0	0	0	0
B- Kalinga 1000	B1	25282	420	24862	4	98	0	25	1	0	0	1	286	0	0	4	0	1	0
C- 500500	C1	25190	318	24872	19	55	17	15	0	67	0	2	143	0	0	0	0	0	0
D- 500500	D1	25259	416	24843	33	69	26	22	1	108	0	0	156	0	0	1	0	0	0
E- Hybride 1000	E1	25220	308	24912	9	35	22	7	1	130	0	1	103	0	0	0	0	0	0
F- Hybride 1000	F1	25218	386	24832	13	34	28	14	1	166	0	1	125	2	0	1	1	0	0
G- T478 11.1 1000	G1	25388	309	25079	8	28	36	3	0	116	0	0	115	2	0	0	0	1	0
H- T478 11.1 1000	H1	25301	342	24959	14	21	22	3	1	140	0	0	134	1	0	6	0	0	0
I- T478 11.1 1000	A2	25298	264	25034	12	34	27	4	0	106	0	3	74	0	0	3	0	0	1
J- T478 11.1 1000	B2	25341	279	25062	14	14	20	16	2	107	0	3	102	0	0	1	0	0	0
K- T478 11.1 1000	C2	25411	268	25143	9	20	9	10	0	92	0	4	119	1	0	2	2	0	0
L- T478 11.1 1000	D2	25429	312	25117	7	20	27	14	0	116	0	1	126	0	0	0	1	0	0
M- T478 11.1 1000	E2	25445	297	25148	6	12	41	5	2	99	0	1	129	1	0	1	0	0	0
N- T478 11.1 1000	F2	25400	292	25108	15	18	13	8	0	124	1	1	111	0	0	1	0	0	0
O- T478 11.1 1000	G2	25204	327	24877	10	19	17	7	0	129	0	0	143	1	0	1	0	0	0
P- T478 11.1 1000	H2	25318	309	25009	9	23	14	6	1	136	0	1	115	0	1	3	0	0	0
Q- T478 11.1 1000	A3	25365	285	25080	15	34	23	3	0	91	0	1	117	0	0	0	0	1	0
R- T478 11.1 1000	B3	25283	294	24989	12	12	35	14	0	105	0	1	111	2	0	1	0	1	0
S- T478 11.1 1000	C3	25379	284	25095	12	18	20	10	1	103	0	1	118	1	0	0	0	0	0
T- T478 11.1 1000	D3	25453	288	25165	7	13	14	8	1	124	0	0	120	0	0	0	1	0	0
U- T478 11.1 1000	E3	25473	316	25157	9	15	10	11	2	143	0	2	120	1	0	1	1	0	1
V- T478 11.1 1000	F3	25428	304	25124	9	25	16	7	1	131	0	0	112	1	0	1	1	0	0
W- T478 11.1 1000	G3	25320	310	25010	16	24	28	6	1	108	0	1	125	1	0	0	0	0	0
H2O	H3	25383	1	25382	1	0	0	0	0	0	0	0	0	0	0	0	0	0	0

Sample	Well	Nombre partitions valides	Nombre partitions positives	Nombre partitions négatives	KiZ1	KaZ4	KiZ3	KaZ2	KaZ4/KiZ1	KiZ3/KiZ1	KiZ3/KaZ4	KaZ2/KiZ1	KaZ2/KaZ4	KaZ2/KiZ3	KiZ3/KaZ4/KiZ1	KaZ2/KaZ4/KiZ1	KaZ2/KiZ3/KiZ1	KaZ2/KiZ3/KaZ4	KaZ2/KiZ3/KaZ4/KiZ1
A- Kitaake 1000	A1	25429	376	25053	17	0	35	0	0	324	0	0	0	0	0	0	0	0	0
B- Kalinga 1000	B1	25308	425	24883	0	59	0	17	0	0	0	0	349	0	0	0	0	0	0
C- 500500	C1	25326	373	24953	15	26	26	5	0	139	0	0	161	1	0	0	0	0	0
D- 500500	D1	25300	367	24933	9	28	8	12	1	167	0	3	137	0	0	1	1	0	0
E- Hybride 1000	E1	25230	318	24912	5	28	11	10	0	135	0	1	126	2	0	0	0	0	0
F- Hybride 1000	F1	25290	320	24970	5	33	22	10	4	123	0	1	121	1	0	0	0	0	0
G- T478 11.1 1000	G1	25462	288	25174	10	18	15	5	1	116	0	1	119	1	0	2	0	0	0
H- T478 11.1 1000	H1	25273	331	24942	9	32	28	10	1	120	0	0	126	2	0	3	0	0	0
I- T478 11.1 1000	A2	25209	292	24917	11	12	29	9	3	111	0	3	112	0	0	2	0	0	0
J- T478 11.1 1000	B2	25482	282	25200	10	19	36	9	0	94	0	0	113	0	0	0	0	1	0
K- T478 11.1 1000	C2	25357	272	25085	3	18	15	4	1	96	0	0	127	5	0	2	0	1	0
L- T478 11.1 1000	D2	25443	276	25167	8	19	34	8	0	83	1	1	121	1	0	0	0	0	0
M- T478 11.1 1000	E2	25452	290	25162	14	32	28	7	0	103	0	1	105	0	0	0	0	0	0
N- T478 11.1 1000	F2	25401	269	25132	12	23	16	6	0	113	0	0	96	2	0	1	0	0	0
O- T478 11.1 1000	G2	25225	309	24916	4	19	22	5	2	125	0	0	132	0	0	0	0	0	0
P- T478 11.1 1000	H2	25391	329	25062	6	26	38	7	2	121	0	0	122	1	0	6	0	0	0
Q- T478 11.1 1000	A3	25181	273	24908	5	20	37	6	0	105	1	2	95	1	0	1	0	0	0
R- T478 11.1 1000	B3	25322	264	25058	14	34	29	6	0	89	0	1	89	0	0	0	1	1	0
S- T478 11.1 1000	C3	25412	275	25137	7	45	14	3	0	126	0	1	76	1	1	0	0	1	0
T- T478 11.1 1000	D3	25397	271	25126	11	32	18	7	0	102	0	1	99	0	0	1	0	0	0
U- T478 11.1 1000	E3	25304	263	25041	10	15	38	5	0	93	0	0	102	0	0	0	0	0	0
V- T478 11.1 1000	F3	25366	248	25118	10	15	27	4	1	74	0	1	115	1	0	0	0	0	0
W- T478 11.1 1000	G3	25393	300	25093	22	27	36	7	3	87	0	3	113	0	1	1	0	0	0
H2O	H3	25213	3	25210	1	1	1	0	0	0	0	0	0	0	0	0	0	0	0

Supplementary Table 2: dPCR results for Chr.7 plants (2/4).

Plant 7b : T478 11.1.

Sample	Well	Nombre partitions valides	Nombre partitions positives	Nombre partitions négatives	KiZ1	KaZ4	KiZ3	KaZ2	KaZ4/KiZ1	KiZ3/KiZ1	KiZ3/KaZ4	KaZ2/KiZ1	KaZ2/KaZ4	KaZ2/KiZ3	KiZ3/KaZ4/KiZ1	KaZ2/KaZ4/KiZ1	KaZ2/KiZ3/KiZ1	KaZ2/KiZ3/KaZ4	KaZ2/KiZ3/KaZ4/KiZ1
A- Kitaake 1000_20230921	A1	25412	260	25152	14	0	28	0	0	218	0	0	0	0	0	0	0	0	0
B- Kalinga 1000_20230921	B1	25202	216	24986	0	41	0	6	0	0	0	0	169	0	0	0	0	0	0
C- 500500_20230921	C1	25351	255	25096	15	32	42	7	0	83	0	0	76	0	0	0	0	0	0
D- 500500_20230921	D1	20982	193	20789	9	16	14	5	0	78	0	0	69	2	0	0	0	0	0
E- 500500_20230921	E1	25449	294	25155	10	13	24	11	0	139	0	0	96	0	0	1	0	0	0
F- 500500_20230921	F1	25359	266	25093	14	21	27	5	0	122	0	1	76	0	0	0	0	0	0
G- 500500_20230921	G1	25444	292	25152	19	20	25	6	0	136	0	2	83	0	0	1	0	0	0
H- 500500_20230921	H1	25349	304	25045	11	16	35	5	1	142	0	2	89	1	0	2	0	0	0
I- 500500_20230921	A2	25255	141	25114	7	21	17	4	0	54	0	0	37	0	0	1	0	0	0
J- Hybride 1000_20230921	B2	25351	185	25166	12	18	19	1	1	72	0	0	60	0	0	2	0	0	0
K- Hybride 1000_20230921	C2	25399	204	25195	8	15	13	8	0	86	0	1	73	0	0	0	0	0	0
L- Hybride 1000_20230921	D2	25353	149	25204	4	18	12	5	0	54	0	0	56	0	0	0	0	0	0
M- Hybride 1000_20230921	E2	25316	180	25136	5	17	10	7	0	71	0	1	68	1	0	0	0	0	0
N- Hybride 1000_20230921	F2	25456	194	25262	7	24	20	4	0	73	0	1	64	0	0	0	1	0	0
O- Hybride 1000_20230921	G2	25200	200	25000	5	26	16	3	1	74	0	1	74	0	0	0	0	0	0
P- Hybride 1000_20230921	H2	25359	259	25100	11	22	21	9	2	103	0	1	88	1	0	1	0	0	0
Q- O2 1000_20230921	A3	25371	158	25213	4	15	9	7	1	67	0	2	53	0	0	0	0	0	0
R- O2 1000_20230921	B3	25446	109	25337	5	11	10	3	1	42	0	1	36	0	0	0	0	0	0
S- O2 1000_20230921	C3	25298	142	25156	7	13	10	3	0	55	0	0	54	0	0	0	0	0	0
T- O2 1000_20230921	D3	25367	145	25222	0	16	10	5	0	57	0	0	56	1	0	0	0	0	0
U- O2 1000_20230921	E3	16625	101	16524	3	10	3	3	0	39	0	0	42	0	0	1	0	0	0
V- O2 1000_20230921	F3	25470	168	25302	12	20	15	2	0	50	0	0	68	0	1	0	0	0	0
W- O2 1000_20230921	G3	25471	194	25277	7	16	11	7	1	83	0	2	64	0	0	3	0	0	0
H20	H3	25381	0	25381	0	0	0	0	0	0	0	0	0	0	0	0	0	0	0

Sample	Well	Nombre partitions valides	Nombre partitions positives	Nombre partitions négatives	KiZ1	KaZ4	KiZ3	KaZ2	KaZ4/KiZ1	KiZ3/KiZ1	KiZ3/KaZ4	KaZ2/KiZ1	KaZ2/KaZ4	KaZ2/KiZ3	KiZ3/KaZ4/KiZ1	KaZ2/KaZ4/KiZ1	KaZ2/KiZ3/KiZ1	KaZ2/KiZ3/KaZ4	KaZ2/KiZ3/KaZ4/KiZ1
00008097A- Kitaake 1000_20230926	A1	25384	326	25058	27	0	27	0	0	272	0	0	0	0	0	0	0	0	0
00008098B- Kalinga 1000_20230926	B1	25272	249	25023	0	91	0	19	0	0	0	0	139	0	0	0	0	0	0
00008099C- 500500_20230926	C1	25370	315	25055	28	37	30	11	2	114	0	0	92	0	0	1	0	0	0
00008100D- 500500_20230926	D1	25413	297	25116	75	58	13	12	0	76	0	0	63	0	0	0	0	0	0
00008101E- 500500_20230926	E1	25383	341	25042	19	46	35	11	0	111	0	1	117	0	0	1	0	0	0
00008102F- 500500_20230926	F1	25290	326	24964	14	32	36	11	2	111	0	1	118	1	0	0	0	0	0
00008103G- 500500_20230926	G1	25446	320	25126	13	46	25	21	0	117	0	0	96	0	0	2	0	0	0
00008104H- 500500_20230926	H1	25363	313	25050	11	52	40	13	2	107	0	0	88	0	0	0	0	0	0
00008105I- 500500_20230926	A2	25220	332	24888	13	29	46	4	0	106	0	0	133	1	0	0	0	0	0
00008106J- Hybride 1000_20230926	B2	25299	237	25062	23	29	9	14	2	96	0	1	62	0	0	0	0	0	1
00008107K- Hybride 1000_20230926	C2	25349	225	25124	14	51	21	7	0	65	0	0	67	0	0	0	0	0	0
00008108L- Hybride 1000_20230926	D2	25413	253	25160	12	23	19	7	0	95	1	0	96	0	0	0	0	0	0
00008109M- Hybride 1000_20230926	E2	25324	201	25123	12	39	25	4	1	64	1	0	54	0	0	1	0	0	0
00008110N- Hybride 1000_20230926	F2	25030	224	24806	23	33	23	3	0	79	0	0	63	0	0	0	0	0	0
00008111O- Hybride 1000_20230926	G2	25328	239	25089	5	26	29	12	0	75	0	1	90	0	0	0	0	1	0
00008112P- Hybride 1000_20230926	H2	25435	262	25173	10	26	39	4	0	74	0	1	107	1	0	0	0	0	0
00008113Q- O2 1000_20230926	A3	25360	151	25209	7	34	24	3	0	43	0	0	40	0	0	0	0	0	0
00008114R- O2 1000_20230926	B3	25455	178	25277	11	42	29	1	0	55	0	0	40	0	0	0	0	0	0
00008115S- O2 1000_20230926	C3	25442	148	25294	9	9	29	2	0	42	0	0	57	0	0	0	0	0	0
00008116T- O2 1000_20230926	D3	25373	205	25168	15	43	20	6	3	50	0	0	68	0	0	0	0	0	0
00008117U- O2 1000_20230926	E3	25419	151	25268	5	21	29	4	0	38	0	1	51	2	0	0	0	0	0
00008118V- O2 1000_20230926	F3	25278	156	25122	5	24	32	11	0	23	0	0	61	0	0	0	0	0	0
00008119W- O2 1000_20230926	G3	25444	180	25264	5	18	16	9	1	67	0	0	63	1	0	0	0	0	0
H20	H3	25354	0	25354	0	0	0	0	0	0	0	0	0	0	0	0	0	0	0

Sample	Well	Nombre partitions valides	Nombre partitions positives	Nombre partitions négatives	KiZ1	KaZ4	KiZ3	KaZ2	KaZ4/KiZ1	KiZ3/KiZ1	KiZ3/KaZ4	KaZ2/KiZ1	KaZ2/KaZ4	KaZ2/KiZ3	KiZ3/KaZ4/KiZ1	KaZ2/KaZ4/KiZ1	KaZ2/KiZ3/KiZ1	KaZ2/KiZ3/KaZ4	KaZ2/KiZ3/KaZ4/KiZ1
A- Kitaake 1000_20231003	A1	25420	112	25308	32	2	30	1	0	47	0	0	0	0	0	0	0	0	0
B- Kalinga 1000_20231003	B1	25363	113	25250	0	53	0	20	0	0	0	0	40	0	0	0	0	0	0
D- 500500_20231003	D1	25407	157	25250	30	19	28	10	0	39	0	0	31	0	0	0	0	0	0
E- 500500_20231003	E1	25387	125	25262	11	36	32	5	0	7	0	0	34	0	0	0	0	0	0
F- 500500_20231003	F1	25253	150	25103	23	35	21	11	1	24	0	0	35	0	0	0	0	0	0
G- 500500_20231003	G1	25409	158	25251	18	31	39	12	0	10	0	0	48	0	0	0	0	0	0
H- 500500_20231003	H1	24441	187	24254	25	34	21	16	0	30	0	0	60	0	0	1	0	0	0
I- 500500_20231003	A2	25229	117	25112	22	26	19	5	0	23	0	0	22	0	0	0	0	0	0
J- Hybride 1000_20231003	B2	25391	110	25281	8	22	11	14	0	27	0	1	27	0	0	0	0	0	0
K- Hybride 1000_20231003	C2	19028	78	18950	9	15	9	3	0	25	0	0	17	0	0	0	0	0	0
L- Hybride 1000_20231003	D2	23582	134	23448	14	25	21	6	0	36	0	0	32	0	0	0	0	0	0
M- Hybride 1000_20231003	E2	25411	159	25252	8	27	24	5	0	49	0	0	46	0	0	0	0	0	0
N- Hybride 1000_20231003	F2	25239	157	25082	10	31	19	4	0	36	0	0	56	1	0	0	0	0	0
O- Hybride 1000_20231003	G2	25270	167	25103	13	30	37	6	0	28	0	0	53	0	0	0	0	0	0
P- Hybride 1000_20231003	H2	25100	155	24945	9	22	51	14	0	16	0	0	43	0	0	0	0	0	0
Q- O2 1000_20231003	A3	25371	211	25160	16	21	14	13	2	69	0	0	72	1	0	2	0	1	0
R- O2 1000_20231003	B3	25418	284	25134	17	40	13	11	0	84	0	2	115	1	0	1	0	0	0
S- O2 1000_20231003	C3	25461	302	25159	12	28	29	20	0	88	0	0	124	1	0	0	0	0	0
T- O2 1000_20231003	D3	25375	308	25067	9	36	26	18	0	87	0	0	132	0	0	0	0	0	0
U- O2 1000_20231003	E3	25377	311	25066	9	22	42	14	1	61	0	0	162	0	0	0	0	0	0
V- O2 1000_20231003	F3	25240	304	24936	10	33	41	23	0	85	0	0	111	0	0	1	0	0	0
W- O2 1000_20231003	G3	25402	330	25072	15	34	29	15	1	88	0	1	146	1	0	0	0	0	0
H20	H3	25270	0	25270	0	0	0	0	0	0	0	0	0	0	0	0	0	0	0

Sample	Well	Nombre partitions valides	Nombre partitions positives	Nombre partitions négatives	KiZ1	KaZ4	KiZ3	KaZ2	KaZ4/KiZ1	KiZ3/KiZ1	KiZ3/KaZ4	KaZ2/KiZ1	KaZ2/KaZ4	KaZ2/KiZ3	KiZ3/KaZ4/KiZ1	KaZ2/KaZ4/KiZ1	KaZ2/KiZ3/KiZ1	KaZ2/KiZ3/KaZ4	KaZ2/KiZ3/KaZ4/KiZ1
A- Kitaake 1000_20231010	A1	25353	478	24875	27	0	54	0	0	397	0	0	0	0	0	0	0	0	0
B- Kalinga 1000_20231010	B1	25315	328	24987	0	65	0	16	0	0	0	0	247	0	0	0	0	0	0
C- 500500_20231010	C1	25406	327	25079	13	12	11	10	0	173	0	4	102	1	0	1	0	0	0
D- 500500_20231010	D1	25316	348	24968	25	22	27	3	0	165	0	1	104	0	0	1	0	0	0
E- 500500_20231010	E1	25284	383	24901	10	26	32	8	0	179	0	1	127	0	0	0	0	0	0
F- 500500_20231010	F1	25314	393	24921	22	31	26	15	0	164	0	4	131	0	0	0	0	0	0
G- 500500_20231010	G1	25449	358	25091	7	23	29	18	0	159	0	2	120	0	0	0	0	0	0
H- 500500_20231010	H1	25399	404	24995	9	29	19	9	2	201	0	4	128	0	0	3	0	0	0
I- 500500_20231010	A2	25120	374	24746	17	25	29	11	3	163	0	1	124	0	0	1	0	0	0
J- Hybride 1000_20231010	B2	25301	360	24941	20	30	22	12	2	117	0	5	148	2	0	0	1	1	0
K- Hybride 1000_20231010	C2	25365	358	25007	16	23	29	15	0	129	0	0	144	1	0	1	0	0	0
L- Hybride 1000_20231010	D2	25439	325	25114	21	23	29	15	1	103	0	1	132	0	0	0	0	0	0
M- Hybride 1000_20231010	E2	25390	306	25084	17	33	22	8	0	109	1	0	113	2	0	0	1	0	0
N- Hybride 1000_20231010	F2	25426	340	25086	6	26	41	20	0	146	0	0	101	0	0	0	0	0	0
O- Hybride 1000_20231010	G2	25296	332	24964	13	25	23	11	0	114	0	1	143	0	0	1	1	0	0
P- Hybride 1000_20231010	H2	25440	374	25066	13	25	20	11	0	163	0	2	138	0	0	2	0	0	0
Q- O2 1000_20231010	A3	25326	249	25077	6	23	32	7	0	75	0	3	101	2	0	0	0	0	0
R- O2 1000_20231010	B3	25468	227	25241	20	23	12	9	0	84	0	0	77	0	0	1	0	1	0
S- O2 1000_20231010	C3	25473	261	25212	6	20	25	9	0	94	0	1	105	1	0	0	0	0	0
T- O2 1000_20231010	D3	25301	224	25077	11	22	9	5	0	95	0	1	80	1	0	0	0	0	0
U- O2 1000_20231010	E3	25379	231	25148	4	31	15	8	0	90	0	2	78	1	0	1	0	1	0
V- O2 1000_20231010	F3	25378	249	25129	15	22	17	13	0	88	0	1	90	0	0	1	0	1	1
W- O2 1000_20231010	G3	25421	213	25208	13	13	27	20	0	52	0	1	86	1	0	0	0	0	0
H20	H3	25320	0	25320	0	0	0	0	0	0	0	0	0	0	0	0	0	0	0

Sample	Well	Nombre partitions valides	Nombre partitions positives	Nombre partitions négatives	KiZ1	KaZ4	KiZ3	KaZ2	KaZ4/KiZ1	KiZ3/KiZ1	KiZ3/KaZ4	KaZ2/KiZ1	KaZ2/KaZ4	KaZ2/KiZ3	KiZ3/KaZ4/KiZ1	KaZ2/KaZ4/KiZ1	KaZ2/KiZ3/KiZ1	KaZ2/KiZ3/KaZ4	KaZ2/KiZ3/KaZ4/KiZ1
A- Kitaake 1000_20231020	A1	25400	361	25039	40	0	46	0	0	275	0	0	0	0	0	0	0	0	0
B- Kalinga 1000_20231020	B1	25325	406	24919	0	62	0	27	0	0	0	0	317	0	0	0	0	0	0
C- 500500_20231020	C1	25385	377	25008	25	28	25	11	0	141	0	3	144	0	0	0	0	0	0
D- 500500_20231020	D1	25408	368	25040	11	37	20	13	1	132	0	0	153	0	0	0	0	0	1
E- 500500_20231020	E1	25379	342	25037	21	57	33	5	0	108	0	0	118	0	0	0	0	0	0
F- 500500_20231020	F1	25224	354	24870	8	42	34	2	0	116	0	0	150	0	0	2	0	0	0
G- 500500_20231020	G1	25377	338	25039	17	41	27	13	0	121	0	4	113	0	0	2	0	0	0
H- 500500_20231020	H1	25341	343	24998	13	37	28	10	3	116	0	0	135	0	0	1	0	0	0
I- 500500_20231020	A2	25144	301	24843	13	35	24	17	0	99	1	3	107	0	0	2	0	0	0
J- Hybride 1000_20231020	B2	25332	265	25067	16	22	15	5	0	107	0	0	99	0	0	1	0	0	0
K- Hybride 1000_20231020	C2	25348	279	25069	11	39	33	2	0	94	0	0	99	0	0	1	0	0	0
L- Hybride 1000_20231020	D2	25389	231	25158	15	25	14	10	1	76	0	0	89	0	0	1	0	0	0
M- Hybride 1000_20231020	E2	25453	231	25222	11	39	10	5	0	94	0	0	70	2	0	0	0	0	0
N- Hybride 1000_20231020	F2	25288	292	24996	12	32	39	7	3	97	0	2	99	1	0	0	0	0	0
O- Hybride 1000_20231020	G2	25224	308	24916	11	36	27	7	1	125	0	1	99	0	0	1	0	0	0
P- Hybride 1000_20231020	H2	25460	338	25122	14	46	31	14	0	139	1	0	93	0	0	0	0	0	0
Q- O2 1000_20231020	A3	25227	196	25031	5	34	12	6	0	83	0	1	54	0	0	1	0	0	0
R- O2 1000_20231020	B3	25438	181	25257	6	18	19	4	0	70	0	0	64	0	0	0	0	0	0
S- O2 1000_20231020	C3	25448	180	25268	6	18	14	8	0	64	0	2	68	0	0	0	0	0	0
T- O2 1000_20231020	D3	25350	182	25168	2	21	15	12	0	73	0	0	57	1	0	0	0	1	0
U- O2 1000_20231020	E3	25426	175	25251	8	8	17	5	0	64	0	0	73	0	0	0	0	0	0
V- O2 1000_20231020	F3	25327	189	25138	9	7	18	7	2	70	0	0	76	0	0	0	0	0	0
W- O2 1000_20231020	G3	25429	193	25236	12	24	6	11	2	67	0	2	68	0	0	1	0	0	0
H20	H3	25366	0	25366	0	0	0	0	0	0	0	0	0	0	0	0	0	0	0

Sample	Well	Nombre partitions valides	Nombre partitions positives	Nombre partitions négatives	KiZ1	KaZ4	KiZ3	KaZ2	KaZ4/KiZ1	KiZ3/KiZ1	KiZ3/KaZ4	KaZ2/KiZ1	KaZ2/KaZ4	KaZ2/KiZ3	KiZ3/KaZ4/KiZ1	KaZ2/KaZ4/KiZ1	KaZ2/KiZ3/KiZ1	KaZ2/KiZ3/KaZ4	KaZ2/KiZ3/KaZ4/KiZ1
A- Kitaake 1000_20231024	A1	25411	328	25083	17	0	25	0	0	286	0	0	0	0	0	0	0	0	0
B- Kalinga 1000_20231024	B1	25319	328	24991	0	67	0	28	0	0	0	0	233	0	0	0	0	0	0
C- 500500_20231024	C1	25395	316	25079	9	33	54	20	0	77	0	0	123	0	0	0	0	0	0
D- 500500_20231024	D1	25405	335	25070	30	45	21	7	1	116	0	0	115	0	0	0	0	0	0
E- 500500_20231024	E1	25415	325	25090	14	31	34	4	2	106	0	0	134	0	0	0	0	0	0
F- 500500_20231024	F1	25383	356	25027	29	38	26	11	0	122	0	3	125	0	0	2	0	0	0
G- 500500_20231024	G1	25429	328	25101	14	39	27	5	0	117	0	3	123	0	0	0	0	0	0
H- 500500_20231024	H1	25412	383	25029	48	56	41	11	3	106	0	3	112	0	0	3	0	0	0
I- 500500_20231024	A2	25146	317	24829	17	42	44	6	0	80	0	1	125	0	0	2	0	0	0
J- Hybride 1000_20231024	B2	25324	247	25077	5	23	24	19	1	97	0	2	76	0	0	0	0	0	0
K- Hybride 1000_20231024	C2	25283	222	25061	14	24	28	6	2	91	0	0	56	0	0	1	0	0	0
L- Hybride 1000_20231024	D2	25408	200	25208	15	26	24	9	0	69	0	1	55	1	0	0	0	0	0
M- Hybride 1000_20231024	E2	25454	204	25250	23	23	20	5	0	50	0	0	81	2	0	0	0	0	0
N- Hybride 1000_20231024	F2	25310	200	25110	15	23	26	5	1	47	0	1	82	0	0	0	0	0	0
O- Hybride 1000_20231024	G2	25331	259	25072	7	34	25	8	1	82	0	1	101	0	0	0	0	0	0
P- Hybride 1000_20231024	H2	25452	317	25135	15	38	24	7	0	120	0	1	109	0	0	2	0	1	0
Q- O2 1000_20231024	A3	25372	143	25229	7	17	9	9	0	55	0	1	44	1	0	0	0	0	0
R- O2 1000_20231024	B3	25421	138	25283	8	16	21	7	0	31	0	0	55	0	0	0	0	0	0
S- O2 1000_20231024	C3	25437	128	25309	5	16	8	5	1	41	0	1	51	0	0	0	0	0	0
T- O2 1000_20231024	D3	25376	171	25205	5	27	20	4	0	56	0	2	57	0	0	0	0	0	0
U- O2 1000_20231024	E3	25406	135	25271	10	9	9	6	0	48	0	0	51	2	0	0	0	0	0
V- O2 1000_20231024	F3	25358	137	25221	4	13	28	8	0	31	0	0	51	1	0	1	0	0	0
W- O2 1000_20231024	G3	25455	183	25272	8	23	9	5	1	68	0	1	66	0	1	1	0	0	0
H20	H3	25339	0	25339	0	0	0	0	0	0	0	0	0	0	0	0	0	0	0

Sample	Well	Nombre partitions valides	Nombre partitions positives	Nombre partitions négatives	KiZ1	KaZ4	KiZ3	KaZ2	KaZ4/KiZ1	KiZ3/KiZ1	KiZ3/KaZ4	KaZ2/KiZ1	KaZ2/KaZ4	KaZ2/KiZ3	KiZ3/KaZ4/KiZ1	KaZ2/KaZ4/KiZ1	KaZ2/KiZ3/KiZ1	KaZ2/KiZ3/KaZ4	KaZ2/KiZ3/KaZ4/KiZ1
A- Kitaake 1000_20231027	A1	25398	352	25046	19	0	41	0	0	292	0	0	0	0	0	0	0	0	0
B- Kalinga 1000_20231027	B1	25383	327	25056	0	58	0	18	0	0	0	0	251	0	0	0	0	0	0
C- 500500_20231027	C1	25261	344	24917	13	27	15	10	0	139	0	2	137	0	0	1	0	0	0
D- 500500_20231027	D1	25262	229	25033	23	8	12	3	0	83	0	1	99	0	0	0	0	0	0
E- 500500_20231027	E1	25328	343	24985	12	23	15	9	0	147	0	1	134	1	0	1	0	0	0
F- 500500_20231027	F1	25398	303	25095	18	21	41	7	0	99	0	0	117	0	0	0	0	0	0
G- 500500_20231027	G1	25264	249	25015	24	15	17	4	1	83	0	1	104	0	0	0	0	0	0
H- 500500_20231027	H1	25447	351	25096	15	29	32	7	0	135	0	0	129	0	0	3	0	1	0
I- 500500_20231027	A2	25444	340	25104	12	28	35	2	0	137	0	4	121	0	0	1	0	0	0
J- Hybride 1000_20231027	B2	25458	254	25204	8	26	16	4	0	94	0	0	106	0	0	0	0	0	0
K- Hybride 1000_20231027	C2	25319	187	25132	18	24	12	4	0	65	0	1	63	0	0	0	0	0	0
L- Hybride 1000_20231027	D2	25432	243	25189	10	25	23	4	0	88	0	0	93	0	0	0	0	0	0
M- Hybride 1000_20231027	E2	25467	242	25225	7	25	15	3	0	93	0	0	99	0	0	0	0	0	0
N- Hybride 1000_20231027	F2	25327	231	25096	11	19	32	6	0	79	0	0	84	0	0	0	0	0	0
O- Hybride 1000_20231027	G2	25446	274	25172	29	25	21	5	0	78	0	0	114	1	0	0	0	1	0
P- Hybride 1000_20231027	H2	25459	290	25169	12	33	17	9	3	105	1	5	102	0	0	3	0	0	0
Q- O2 1000_20231027	A3	25317	186	25131	11	19	17	8	0	53	0	1	76	0	0	1	0	0	0
R- O2 1000_20231027	B3	25407	164	25243	5	14	20	2	0	52	0	0	69	2	0	0	0	0	0
S- O2 1000_20231027	C3	25472	155	25317	3	27	21	5	0	46	0	0	52	1	0	0	0	0	0
T- O2 1000_20231027	D3	25469	166	25303	2	20	24	4	0	36	0	0	80	0	0	0	0	0	0
U- O2 1000_20231027	E3	25442	169	25273	6	29	26	3	0	39	0	0	66	0	0	0	0	0	0
V- O2 1000_20231027	F3	25392	189	25203	15	20	5	6	0	59	0	0	84	0	0	0	0	0	0
W- O2 1000_20231027	G3	25461	190	25271	21	31	18	5	1	46	1	0	67	0	0	0	0	0	0
H20	H3	25400	0	25400	0	0	0	0	0	0	0	0	0	0	0	0	0	0	0

Sample	Well	Nombre partitions valides	Nombre partitions positives	Nombre partitions négatives	KiZ1	KaZ4	KiZ3	KaZ2	KaZ4/KiZ1	KiZ3/KiZ1	KiZ3/KaZ4	KaZ2/KiZ1	KaZ2/KaZ4	KaZ2/KiZ3	KiZ3/KaZ4/KiZ1	KaZ2/KaZ4/KiZ1	KaZ2/KiZ3/KiZ1	KaZ2/KiZ3/KaZ4	KaZ2/KiZ3/KaZ4/KiZ1
A- Kitaake 1000_20231107	A1	25424	440	24984	44	0	126	0	0	270	0	0	0	0	0	0	0	0	0
B- Kalinga 1000_20231107	B1	25307	374	24933	0	114	0	40	0	0	0	0	220	0	0	0	0	0	0
C- 500500_20231107	C1	25370	323	25047	40	41	62	41	0	61	0	0	78	0	0	0	0	0	0
D- 500500_20231107	D1	25382	308	25074	59	49	22	20	1	68	0	0	89	0	0	0	0	0	0
E- 500500_20231107	E1	25378	321	25057	45	34	43	28	0	40	0	0	130	1	0	0	0	0	0
F- 500500_20231107	F1	25157	304	24853	51	36	19	31	0	68	0	0	99	0	0	0	0	0	0
G- 500500_20231107	G1	25458	314	25144	25	32	68	33	0	42	0	0	114	0	0	0	0	0	0
H- 500500_20231107	H1	25266	394	24872	40	47	43	30	2	119	1	0	112	0	0	0	0	0	0
I- 500500_20231107	A2	25129	372	24757	34	38	33	21	1	126	0	0	119	0	0	0	0	0	0
J- Hybride 1000_20231107	B2	25353	314	25039	43	28	17	17	0	96	0	0	113	0	0	0	0	0	0
K- Hybride 1000_20231107	C2	25317	290	25027	20	34	27	17	0	87	0	0	105	0	0	0	0	0	0
L- Hybride 1000_20231107	D2	25322	298	25024	27	42	27	12	0	107	0	0	82	1	0	0	0	0	0
M- Hybride 1000_20231107	E2	25431	268	25163	14	27	50	26	0	62	0	0	89	0	0	0	0	0	0
N- Hybride 1000_20231107	F2	25334	311	25023	12	41	112	17	0	11	0	0	117	1	0	0	0	0	0
O- Hybride 1000_20231107	G2	25292	316	24976	6	43	146	24	0	2	0	0	95	0	0	0	0	0	0
P- Hybride 1000_20231107	H2	25434	390	25044	13	49	117	32	0	62	1	0	115	1	0	0	0	0	0
Q- O2 1000_20231107	A3	25277	164	25113	9	30	26	9	0	37	0	1	51	1	0	0	0	0	0
R- O2 1000_20231107	B3	25492	162	25330	8	18	42	18	0	19	0	0	56	1	0	0	0	0	0
S- O2 1000_20231107	C3	25471	154	25317	13	12	31	17	0	26	1	0	54	0	0	0	0	0	0
T- O2 1000_20231107	D3	25408	167	25241	15	16	22	19	0	40	0	0	54	1	0	0	0	0	0
U- O2 1000_20231107	E3	25465	172	25293	21	22	9	15	0	40	0	0	65	0	0	0	0	0	0
V- O2 1000_20231107	F3	25309	187	25122	9	21	29	21	0	52	0	0	55	0	0	0	0	0	0
W- O2 1000_20231107	G3	25463	215	25248	17	25	30	18	0	55	0	0	70	0	0	0	0	0	0
H20	H3	25329	0	25329	0	0	0	0	0	0	0	0	0	0	0	0	0	0	0

Supporting Table 2: dPCR results for Chr.7 plants (3/4).

Plant 7a/1 : Plant O2.

Sample	Well	Nombre partitions valides	Nombre partitions positives	Nombre partitions negatives	KiZ1	KaZ4	KiZ3	KaZ2	KaZ4/KiZ1	KiZ3/KiZ1	KiZ3/KaZ4	KaZ2/KiZ1	KaZ2/KaZ4	KaZ2/KiZ3	KiZ3/KaZ4/KiZ1	KaZ2/KaZ4/KiZ1	KaZ2/KiZ3/KiZ1	KaZ2/KiZ3/KaZ4	KaZ2/KiZ3/KaZ4/KiZ1
A1- Kitaake 1000_20240326	A1	25220	338	24882	99	0	93	0	0	146	0	0	0	0	0	0	0	0	0
B1- Kalinga 1000_20240326	B1	25274	353	24921	0	88	0	71	0	0	0	0	194	0	0	0	0	0	0
C1- 500-500_20240326	C1	25340	187	25153	10	10	80	4	0	77	0	0	6	0	0	0	0	0	0
D1- 500-500_20240326	D1	25397	262	25135	22	32	57	38	0	41	0	0	72	0	0	0	0	0	0
E1- 500-500_20240326	E1	25282	217	25065	28	42	44	24	0	27	0	0	52	0	0	0	0	0	0
F1- 500-500_20240326	F1	25322	309	25013	48	35	54	32	0	47	0	2	91	0	0	0	0	0	0
G1- 500-500_20240326	G1	25415	288	25127	9	38	82	45	0	30	0	0	84	0	0	0	0	0	0
H1- 500-500_20240326	H1	25430	318	25112	39	49	50	40	0	29	0	0	111	0	0	0	0	0	0
I1- 500-500_20240326	A2	25076	286	24790	21	44	62	23	0	37	0	0	99	0	0	0	0	0	0
J1- Hybride 1000_20240326	B2	25438	293	25145	27	66	52	26	0	31	1	0	90	0	0	0	0	0	0
K1- Hybride 1000_20240326	C2	25299	249	25050	41	50	24	27	0	40	0	0	67	0	0	0	0	0	0
L1- Hybride 1000_20240326	D2	25473	295	25178	54	76	32	10	0	33	0	0	90	0	0	0	0	0	0
M1- Hybride 1000_20240326	E2	25460	263	25197	38	5	41	107	0	43	0	0	28	0	0	0	0	1	0
N1- Hybride 1000_20240326	F2	25376	293	25083	38	61	44	30	0	48	0	1	71	0	0	0	0	0	0
O1- Hybride 1000_20240326	G2	25326	220	25106	13	55	55	20	0	2	0	0	73	2	0	0	0	0	0
P1- Hybride 1000_20240326	H2	25398	288	25110	16	52	73	23	0	54	0	0	69	1	0	0	0	0	0
Q1- O5 1000_20240326	A3	25163	244	24919	23	28	41	48	0	24	0	0	80	0	0	0	0	0	0
R1- O5 1000_20240326	B3	25469	314	25155	25	34	42	36	0	71	0	1	104	1	0	0	0	0	0
S1- O5 1000_20240326	C3	25447	297	25150	23	50	49	23	0	47	0	0	105	0	0	0	0	0	0
T1- O5 1000_20240326	D3	25353	262	25091	35	40	42	36	0	37	0	0	71	1	0	0	0	0	0
U1- O5 1000_20240326	E3	25427	291	25136	36	58	47	29	0	31	0	0	89	0	0	1	0	0	0
V1- O5 1000_20240326	F3	25349	249	25100	10	32	72	42	0	24	0	0	69	0	0	0	0	0	0
W1- O5 1000_20240326	G3	25433	298	25135	28	74	37	22	0	50	0	1	85	1	0	0	0	0	0
H201	H3	25251	1	25250	1	0	0	0	0	0	0	0	0	0	0	0	0	0	0

Sample	Well	Nombre partitions valides	Nombre partitions positives	Nombre partitions negatives	KiZ1	KaZ4	KiZ3	KaZ2	KaZ4/KiZ1	KiZ3/KiZ1	KiZ3/KaZ4	KaZ2/KiZ1	KaZ2/KaZ4	KaZ2/KiZ3	KiZ3/KaZ4/KiZ1	KaZ2/KaZ4/KiZ1	KaZ2/KiZ3/KiZ1	KaZ2/KiZ3/KaZ4	KaZ2/KiZ3/KaZ4/KiZ1
A2- Kitaake 1000_20240326	A1	25300	204	25096	57	0	90	0	0	57	0	0	0	0	0	0	0	0	0
B2- Kalinga 1000_20240326	B1	25372	402	24970	0	89	0	49	0	0	0	0	263	0	0	1	0	0	0
C2- 500-500_20240326	C1	25405	320	25085	23	31	53	45	0	52	0	0	116	0	0	0	0	0	0
D2- 500-500_20240326	D1	25415	280	25135	42	33	33	59	0	26	0	0	87	0	0	0	0	0	0
E2- 500-500_20240326	E1	25293	294	24999	20	76	56	13	0	51	0	1	77	0	0	0	0	0	0
F2- 500-500_20240326	F1	25277	419	24858	36	55	56	32	0	102	0	0	138	0	0	0	0	0	0
G2- 500-500_20240326	G1	25315	263	25052	17	43	43	30	1	16	0	0	113	0	0	0	0	0	0
H2- 500-500_20240326	H1	25463	313	25150	3	33	123	54	0	8	0	1	91	0	0	0	0	0	0
I2- 500-500_20240326	A2	25402	325	25077	18	60	81	21	0	37	0	0	108	0	0	0	0	0	0
J2- Hybride 1000_20240326	B2	25404	258	25146	21	36	38	51	0	11	0	0	101	0	0	0	0	0	0
K2- Hybride 1000_20240326	C2	25382	281	25101	40	70	6	37	0	7	0	0	121	0	0	0	0	0	0
L2- Hybride 1000_20240326	D2	25430	232	25198	22	32	25	31	0	48	0	0	74	0	0	0	0	0	0
M2- Hybride 1000_20240326	E2	25362	207	25155	11	19	63	67	0	14	0	0	32	1	0	0	0	0	0
N2- Hybride 1000_20240326	F2	25353	308	25045	33	21	60	50	0	29	0	0	115	0	0	0	0	0	0
O2- Hybride 1000_20240326	G2	25434	250	25184	20	68	45	18	0	21	0	0	78	0	0	0	0	0	0
P2- Hybride 1000_20240326	H2	25446	224	25222	33	22	30	68	0	19	0	0	52	0	0	0	0	0	0
Q2- O5 1000_20240326	A3	25260	322	24938	41	45	30	42	0	52	0	0	112	0	0	0	0	0	0
R2- O5 1000_20240326	B3	25295	358	24937	5	28	143	51	0	9	0	0	120	1	0	0	1	0	0
S2- O5 1000_20240326	C3	25389	218	25171	13	28	52	52	0	12	0	0	61	0	0	0	0	0	0
T2- O5 1000_20240326	D3	25435	268	25167	23	51	36	19	0	42	0	0	96	1	0	0	0	0	0
U2- O5 1000_20240326	E3	25333	287	25046	25	39	55	21	0	27	0	0	119	0	0	0	0	1	0
V2- O5 1000_20240326	F3	25432	284	25148	23	37	61	31	0	41	0	0	91	0	0	0	0	0	0
W2- O5 1000_20240326	G3	25425	311	25114	21	24	46	58	0	53	0	0	109	0	0	0	0	0	0
H202	H3	25450	0	25450	0	0	0	0	0	0	0	0	0	0	0	0	0	0	0

Sample	Well	Nombre partitions valides	Nombre partitions positives	Nombre partitions negatives	KiZ1	KaZ4	KiZ3	KaZ2	KaZ4/KiZ1	KiZ3/KiZ1	KiZ3/KaZ4	KaZ2/KiZ1	KaZ2/KaZ4	KaZ2/KiZ3	KiZ3/KaZ4/KiZ1	KaZ2/KaZ4/KiZ1	KaZ2/KiZ3/KiZ1	KaZ2/KiZ3/KaZ4	KaZ2/KiZ3/KaZ4/KiZ1
A- Kitaake 1000_20240329	A1	25309	419	24890	19	0	206	1	0	193	0	0	0	0	0	0	0	0	0
B- Kalinga 1000_20240329	B1	25244	388	24856	0	131	0	38	0	0	0	0	219	0	0	0	0	0	0
C- 500-500_20240329	C1	25231	370	24861	17	48	66	31	0	102	0	0	106	0	0	0	0	0	0
D- 500-500_20240329	D1	25306	365	24941	56	64	25	28	0	73	0	0	119	0	0	0	0	0	0
E- 500-500_20240329	E1	25368	287	25081	41	63	18	25	0	37	0	0	103	0	0	0	0	0	0
F- 500-500_20240329	F1	25372	382	24990	19	49	95	26	0	54	0	0	139	0	0	0	0	0	0
G- 500-500_20240329	G1	25333	336	24997	48	93	30	9	0	49	1	0	106	0	0	0	0	0	0
H- 500-500_20240329	H1	25390	398	24992	10	43	111	45	0	44	0	0	145	0	0	0	0	0	0
I- 500-500_20240329	A2	25390	298	25092	28	74	49	21	1	39	0	0	86	0	0	0	0	0	0
J- Hybride 1000_20240329	B2	25370	310	25060	40	35	40	28	0	59	0	0	108	0	0	0	0	0	0
K- Hybride 1000_20240329	C2	25331	332	24999	9	56	72	25	0	70	0	0	100	0	0	0	0	0	0
L- Hybride 1000_20240329	D2	25350	355	24995	29	27	47	30	0	104	1	0	115	2	0	0	0	0	0
M- Hybride 1000_20240329	E2	25411	288	25123	13	40	71	15	0	28	0	0	120	1	0	0	0	0	0
N- Hybride 1000_20240329	F2	25333	333	25000	18	42	53	22	0	67	1	0	130	0	0	0	0	0	0
O- Hybride 1000_20240329	G2	25321	377	24944	19	47	86	25	0	65	0	0	134	1	0	0	0	0	0
P- Hybride 1000_20240329	H2	25436	376	25060	7	42	144	38	0	27	0	0	117	1	0	0	0	0	0
Q- O5 1000_20240329	A3	25186	331	24855	40	29	34	18	0	71	0	1	138	0	0	0	0	0	0
R- O5 1000_20240329	B3	25238	341	24897	26	41	49	15	0	77	0	0	133	0	0	0	0	0	0
S- O5 1000_20240329	C3	25411	330	25081	19	51	50	25	0	71	0	0	113	1	0	0	0	0	0
T- O5 1000_20240329	D3	25349	319	25030	22	41	31	18	0	73	0	0	134	0	0	0	0	0	0
U- O5 1000_20240329	E3	25338	334	25004	39	28	29	17	0	78	0	0	143	0	0	0	0	0	0
V- O5 1000_20240329	F3	25317	337	24980	19	28	51	28	1	58	0	0	152	0	0	0	0	0	0
W- O5 1000_20240329	G3	25368	364	25004	39	37	45	25	0	87	0	1	130	0	0	0	0	0	0
H20	H3	25410	0	25410	0	0	0	0	0	0	0	0	0	0	0	0	0	0	0

Sample	Well	Nombre partitions valides	Nombre partitions positives	Nombre partitions negatives	KiZ1	KaZ4	KiZ3	KaZ2	KaZ4/KiZ1	KiZ3/KiZ1	KiZ3/KaZ4	KaZ2/KiZ1	KaZ2/KaZ4	KaZ2/KiZ3	KiZ3/KaZ4/KiZ1	KaZ2/KaZ4/KiZ1	KaZ2/KiZ3/KiZ1	KaZ2/KiZ3/KaZ4	KaZ2/KiZ3/KaZ4/KiZ1
A1- Kitaake 1000_20240326	A1	25198	336	24862	18	0	74	1	0	243	0	0	0	0	0	0	0	0	0
B1- Kalinga 1000_20240326	B1	25185	258	24927	0	87	0	14	1	0	0	0	156	0	0	0	0	0	0
C1- 500-500_20240326	C1	25176	276	24900	17	30	41	8	0	98	0	0	82	0	0	0	0	0	0
D1- 500-500_20240326	D1	25287	294	24993	23	27	40	6	0	86	0	0	111	0	0	1	0	0	0
E1- 500-500_20240326	E1	25217	265	24952	33	40	36	18	0	84	0	0	54	0	0	0	0	0	0
F1- 500-500_20240326	F1	25323	274	25049	15	39	70	15	0	66	0	0	69	0	0	0	0	0	0
G1- 500-500_20240326	G1	25304	312	24992	13	41	63	15	0	83	0	0	97	0	0	0	0	0	0
H1- 500-500_20240326	H1	25366	282	25084	15	58	61	15	0	71	0	0	61	0	0	1	0	0	0
I1- 500-500_20240326	A2	25192	287	24905	9	48	86	21	0	62	0	1	60	0	0	0	0	0	0
J1- Hybride 1000_20240326	B2	25440	249	25191	12	28	42	38	0	66	0	0	62	0	0	0	0	1	0
K1- Hybride 1000_20240326	C2	25385	208	25177	11	30	50	25	0	37	0	0	55	0	0	0	0	0	0
L1- Hybride 1000_20240326	D2	25384	232	25152	14	33	53	12	0	48	0	0	72	0	0	0	0	0	0
M1- Hybride 1000_20240326	E2	25417	239	25178	11	43	50	9	0	63	0	0	62	0	0	1	0	0	0
N1- Hybride 1000_20240326	F2	25442	240	25202	17	52	47	12	0	55	0	0	57	0	0	0	0	0	0
O1- Hybride 1000_20240326	G2	25345	243	25102	11	40	50	23	0	28	0	0	90	0	0	1	0	0	0
P1- Hybride 1000_20240326	H2	25423	261	25162	25	50	25	20	0	80	0	2	59	0	0	0	0	0	0
Q1- O5 1000_20240326	A3	25311	262	25049	16	40	51	17	0	72	0	0	65	0	0	0	0	1	0
R1- O5 1000_20240326	B3	25468	289	25179	23	47	52	19	0	70	0	1	77	0	0	0	0	0	0
S1- O5 1000_20240326	C3	25450	278	25172	16	27	60	21	0	54	0	0	100	0	0	0	0	0	0
T1- O5 1000_20240326	D3	24825	297	24528	24	27	66	39	0	73	0	0	68	0	0	0	0	0	0
U1- O5 1000_20240326	E3	25449	294	25155	19	48	50	21	0	62	0	0	93	0	0	0	0	1	0
V1- O5 1000_20240326	F3	25361	218	25143	15	33	36	20	0	51	0	0	63	0	0	0	0	0	0
W1- O5 1000_20240326	G3	25434	304	25130	26	43	71	21	0	55	1	0	84	1	0	2	0	0	0
H201	H3	25304	0	25304	0	0	0	0	0	0	0	0	0	0	0	0	0	0	0

Sample	Well	Nombre partitions valides	Nombre partitions positives	Nombre partitions negatives	KiZ1	KaZ4	KiZ3	KaZ2	KaZ4/KiZ1	KiZ3/KiZ1	KiZ3/KaZ4	KaZ2/KiZ1	KaZ2/KaZ4	KaZ2/KiZ3	KiZ3/KaZ4/KiZ1	KaZ2/KaZ4/KiZ1	KaZ2/KiZ3/KiZ1	KaZ2/KiZ3/KaZ4	KaZ2/KiZ3/KaZ4/KiZ1
A2- Kitaake 1000_20240326	A1	25338	382	24956	35	0	47	0	0	300	0	0	0	0	0	0	0	0	0
B2- Kalinga 1000_20240326	B1	25351	281	25070	1	68	0	18	0	0	0	0	194	0	0	0	0	0	0
C2- 500-500_20240326	C1	25381	316	25065	19	57	41	11	0	89	0	1	98	0	0	0	0	0	0
D2- 500-500_20240326	D1	25302	311	24991	29	51	27	11	0	103	0	0	90	0	0	0	0	0	0
E2- 500-500_20240326	E1	22114	222	21892	12	38	42	11	0	54	0	0	65	0	0	0	0	0	0
F2- 500-500_20240326	F1	25250	318	24932	22	42	45	12	0	80	0	0	117	0	0	0	0	0	0
G2- 500-500_20240326	G1	25211	286	24925	11	28	45	15	0	78	0	0	108	0	0	1	0	0	0
H2- 500-500_20240326	H1	25395	332	25063	16	50	70	14	1	68	0	0	112	0	0	1	0	0	0
I2- 500-500_20240326	A2	25308	306	25002	19	36	45	22	0	91	0	0	93	0	0	0	0	0	0
J2- Hybride 1000_20240326	B2	25399	298	25101	14	44	54	13	0	84	0	0	89	0	0	0	0	0	0
K2- Hybride 1000_20240326	C2	25393	297	25096	17	23	59	16	0	73	0	0	109	0	0	0	0	0	0
L2- Hybride 1000_20240326	D2	25448	284	25164	14	56	48	16	0	58	0	0	92	0	0	0	0	0	0
M2- Hybride 1000_20240326	E2	25488	270	25218	16	45	48	8	0	79	0	0	73	1	0	0	0	0	0
N2- Hybride 1000_20240326	F2	25377	244	25133	16	37	35	9	0	57	0	0	90	0	0	0	0	0	0
O2- Hybride 1000_20240326	G2	25338	303	25035	29	62	34	9	0	60	0	0	108	0	0	0	0	1	0
P2- Hybride 1000_20240326	H2	25349	291	25058	7	39	64	36	1	45	0	0	99	0	0	0	0	0	0
Q2- O5 1000_20240326	A3	25368	293	25075	27	64	30	12	0	82	0	0	77	1	0	0	0	0	0
R2- O5 1000_20240326	B3	25273	273	25000	11	24	64	25	1	54	0	0	93	1	0	0	0	0	0
S2- O5 1000_20240326	C3	25424	333	25091	22	42	27	16	1	110	0	1	113	0	0	0	1	0	0
T2- O5 1000_20240326	D3	25404	330	25074	25	53	47	12	0	76	0	0	117	0	0	0	0	0	0
U2- O5 1000_20240326	E3	25380	249	25131	24	34	22	20	0	74	0	0	75	0	0	0	0	0	0
V2- O5 1000_20240326	F3	25377	276	25101	15	37	39	11	0	80	0	0	93	0	0	1	0	0	0
W2- O5 1000_20240326	G3	25338	249	25089	13	32	47	11	0	58	0	0	88	0	0	0	0	0	0
H202	H3	25453	0	25453	0	0	0	0	0	0	0	0	0	0	0	0	0	0	0

Sample	Well	Nombre partitions valides	Nombre partitions positives	Nombre partitions negatives	KiZ1	KaZ4	KiZ3	KaZ2	KaZ4/KiZ1	KiZ3/KiZ1	KiZ3/KaZ4	KaZ2/KiZ1	KaZ2/KaZ4	KaZ2/KiZ3	KiZ3/KaZ4/KiZ1	KaZ2/KaZ4/KiZ1	KaZ2/KiZ3/KiZ1	KaZ2/KiZ3/KaZ4	KaZ2/KiZ3/KaZ4/KiZ1
A1- Kitaake 1000_20240419	A1	25195	229	24966	31	0	70	0	0	127	0	0	0	1	0	0	0	0	0
B1- Kalinga 1000_20240419	B1	25331	339	24992	0	115	0	37	0	0	0	0	187	0	0	0	0	0	0
C1- 500-500_20240419	C1	25283	297	24986	28	45	20	22	2	62	0	0	118	0	0	0	0	0	0
D1- 500-500_20240419	D1	25250	271	24979	21	41	34	27	0	42	0	0	106	0	0	0	0	0	0
E1- 500-500_20240419	E1	25164	318	24846	9	33	63	33	0	71	0	0	109	0	0	0	0	0	0
F1- 500-500_20240419	F1	25378	305	25073	34	30	32	20	0	60	0	0	129	0	0	0	0	0	0
G1- 500-500_20240419	G1	25393	304	25089	22	43	48	19	0	45	0	0	127	0	0	0	0	0	0
H1- 500-500_20240419	H1	25394	338	25056	11	45	90	25	0	45	0	1	121	0	0	0	0	0	0
I1- 500-500_20240419	A2	25333	327	25006	9	45	78	23	0	42	0	0	130	0	0	0	0	0	0
J1- Hybride 1000_20240419	B2	25373	348	25025	44	33	26	14	0	87	0	0	144	0	0	0	0	0	0
K1- Hybride 1000_20240419	C2	25228	334	24894	41	45	36	20	0	90	0	0	101	1	0	0	0	0	0
L1- Hybride 1000_20240419	D2	25377	296	25081	4	43	83	31	0	40	0	0	95	0	0	0	0	0	0
M1- Hybride 1000_20240419	E2	25341	287	25054	25	32	29	29	0	53	0	0	119	0	0	0	0	0	0
N1- Hybride 1000_20240419	F2	25297	330	24967	20	32	32	44	0	123	0	0	79	0	0	0	0	0	0
O1- Hybride 1000_20240419	G2	25378	339	25039	15	30	49	22	0	88	0	0	135	0	0	0	0	0	0
P1- Hybride 1000_20240419	H2	25457	350	25107	3	44	138	26	0	16	0	0	122	1	0	0	0	0	0
Q1- O5 1000_20240419	A3	25215	285	24930	27	28	18	33	0	79	0	0	99	1	0	0	0	0	0
R1- O5 1000_20240419	B3	25366	252	25114	15	42	32	7	0	49	0	0	107	0	0	0	0	0	0
S1- O5 1000_20240419	C3	25251	327	24924	6	27	55	14	0	87	0	1	137	0	0	0	0	0	0
T1- O5 1000_20240419	D3	25434	278	25156	23	35	32	21	0	71	0	0	96	0	0	0	0	0	0
U1- O5 1000_20240419	E3	25437	290	25147	29	36	40	16	0	70	0	0	99	0	0	0	0	0	0
V1- O5 1000_20240419	F3	25411	289	25122	17	33	54	22	0	59	0	0	104	0	0	0	0	0	0
W1- O5 1000_20240419	G3	25403	322	25081	18	28	51	31	0	56	0	0	138	0	0	0	0	0	0
H201	H3	25366	0	25366	0	0	0	0	0	0	0	0	0	0	0	0	0	0	0

Sample	Well	Nombre partitions valides	Nombre partitions positives	Nombre partitions negatives	KiZ1	KaZ4	KiZ3	KaZ2	KaZ4/KiZ1	KiZ3/KiZ1	KiZ3/KaZ4	KaZ2/KiZ1	KaZ2/KaZ4	KaZ2/KiZ3	KiZ3/KaZ4/KiZ1	KaZ2/KaZ4/KiZ1	KaZ2/KiZ3/KiZ1	KaZ2/KiZ3/KaZ4	KaZ2/KiZ3/KaZ4/KiZ1
A1- Kitaake 1000_20240423	A1	25320	262	25058	36	0	100	0	0	126	0	0	0	0	0	0	0	0	0
B1- Kalinga 1000_20240423	B1	25330	400	24930	0	64	0	31	0	0	0	0	305	0	0	0	0	0	0
C1- 500-500_20240423	C1	25381	343	25038	26	42	34	17	0	65	0	1	158	0	0	0	0	0	0
D1- 500-500_20240423	D1	25281	328	24953	17	38	51	19	0	59	0	0	144	0	0	0	0	0	0
E1- 500-500_20240423	E1	25403	303	25100	18	27	49	20	0	51	0	1	137	0	0	0	0	0	0
F1- 500-500_20240423	F1	25301	340	24961	26	48	39	16	0	65	0	0	146	0	0	0	0	0	0
G1- 500-500_20240423	G1	25378	332	25046	20	59	33	17	0	57	0	0	146	0	0	0	0	0	0
H1- 500-500_20240423	H1	25353	323	25030	15	41	81	29	0	13	0	0	144	0	0	0	0	0	0
I1- 500-500_20240423	A2	25422	324	25098	5	50	76	12	0	40	0	0	141	0	0	0	0	0	0
J1- Hybride 1000_20240423	B2	25431	306	25125	21	22	91	34	0	21	0	0	115	2	0	0	0	0	0
K1- Hybride 1000_20240423	C2	25362	387	24975	13	33	146	29	0	41	0	0	125	0	0	0	0	0	0
L1- Hybride 1000_20240423	D2	25396	360	25036	43	52	47	35	0	60	1	0	122	0	0	0	0	0	0
M1- Hybride 1000_20240423	E2	25382	374	25008	21	25	41	41	0	120	0	0	125	1	0	0	0	0	0
N1- Hybride 1000_20240423	F2	25353	360	24993	45	31	67	45	0	69	0	0	103	0	0	0	0	0	0
O1- Hybride 1000_20240326	G2	25338	365	24973	12	30	113	54	0	23	0	0	132	1	0	0	0	0	0
P1- Hybride 1000_20240423	H2	25456	348	25108	4	41	119	45	0	1	0	0	135	3	0	0	0	0	0
Q1- O5 1000_20240423	A3	25261	327	24934	26	46	50	29	0	64	0	1	111	0	0	0	0	0	0
R1- O5 1000_20240423	B3	25415	364	25051	39	43	62	31	0	72	0	1	116	0	0	0	0	0	0
S1- O5 1000_20240423	C3	25368	332	25036	27	35	73	30	0	46	0	2	119	0	0	0	0	0	0
T1- O5 1000_20240423	D3	25347	369	24978	43	38	26	27	0	113	0	0	122	0	0	0	0	0	0
U1- O5 1000_20240423	E3	25375	323	25052	22	48	40	35	0	56	0	0	121	1	0	0	0	0	0
V1- O5 1000_20240423	F3	25426	348	25078	16	32	86	33	0	46	0	0	135	0	0	0	0	0	0
W1- O5 1000_20240423	G3	25303	350	24953	24	48	51	25	0	76	0	0	126	0	0	0	0	0	0
H201	H3	25324	0	25324	0	0	0	0	0	0	0	0	0	0	0	0	0	0	0

Sample	Well	Nombre partitions valides	Nombre partitions positives	Nombre partitions negatives	KiZ1	KaZ4	KiZ3	KaZ2	KaZ4/KiZ1	KiZ3/KiZ1	KiZ3/KaZ4	KaZ2/KiZ1	KaZ2/KaZ4	KaZ2/KiZ3	KiZ3/KaZ4/KiZ1	KaZ2/KaZ4/KiZ1	KaZ2/KiZ3/KiZ1	KaZ2/KiZ3/KaZ4	KaZ2/KiZ3/KaZ4/KiZ1
A2- Kitaake 1000_20240423	A1	25311	258	25053	38	0	106	0	0	114	0	0	0	0	0	0	0	0	0
B2- Kalinga 1000_20240423	B1	25325	368	24957	0	118	0	40	0	0	0	0	210	0	0	0	0	0	0
C2- 500-500_20240423	C1	25336	267	25069	31	47	53	36	0	26	0	0	74	0	0	0	0	0	0
D2- 500-500_20240423	D1	25276	343	24933	35	72	36	18	0	82	0	0	100	0	0	0	0	0	0
E2- 500-500_20240423	E1	25256	295	24961	28	37	36	37	0	48	0	0	109	0	0	0	0	0	0
F2- 500-500_20240423	F1	25361	361	25000	37	79	49	14	0	72	0	0	110	0	0	0	0	0	0
G2- 500-500_20240423	G1	25259	327	24932	23	35	42	32	0	85	0	0	109	1	0	0	0	0	0
H2- 500-500_20240423	H1	25356	325	25031	15	39	86	50	0	42	0	0	93	0	0	0	0	0	0
I2- 500-500_20240423	A2	25129	311	24818	23	53	35	32	0	59	0	0	109	0	0	0	0	0	0
J2- Hybride 1000_20240423	B2	25410	358	25052	40	55	60	21	0	71	0	0	111	0	0	0	0	0	0
K2- Hybride 1000_20240423	C2	25267	312	24955	37	30	33	33	0	104	0	0	74	0	0	0	0	1	0
L2- Hybride 1000_20240423	D2	25282	361	24921	34	37	68	45	0	80	0	0	94	3	0	0	0	0	0
M2- Hybride 1000_20240423	E2	25443	318	25125	32	52	72	27	0	46	0	0	88	1	0	0	0	0	0
N2- Hybride 1000_20240423	F2	25403	293	25110	30	36	65	37	0	43	0	0	81	1	0	0	0	0	0
O2- Hybride 1000_20240423	G2	25224	400	24824	48	37	59	30	0	97	0	0	129	0	0	0	0	0	0
P2- Hybride 1000_20240423	H2	25369	350	25019	23	38	96	23	0	57	0	1	112	0	0	0	0	0	0
Q2- O5 1000_20240423	A3	25267	319	24948	11	38	106	36	0	29	0	0	98	1	0	0	0	0	0
R2- O5 1000_20240423	B3	25444	341	25103	25	50	49	32	0	77	0	1	106	1	0	0	0	0	0
S2- O5 1000_20240423	C3	25429	328	25101	29	24	37	36	1	99	0	0	100	2	0	0	0	0	0
T2- O5 1000_20240423	D3	25319	331	24988	35	48	51	22	0	81	1	1	91	0	0	0	0	1	0
U2- O5 1000_20240423	E3	25386	307	25079	32	53	32	24	0	71	0	0	95	0	0	0	0	0	0
V2- O5 1000_20240423	F3	25381	315	25066	24	50	41	28	0	78	0	0	94	0	0	0	0	0	0
W2- O5 1000_20240423	G3	25482	348	25134	16	50	129	36	0	11	0	0	105	1	0	0	0	0	0
H202	H3	25273	0	25273	0	0	0	0	0	0	0	0	0	0	0	0	0	0	0

Supplementary Table 2: dPCR results for Chr.7 plants (4/4).

Plant 7a/2 : Plant O5.
