# Supplementary material for: dCas9-SPO11-1 locally stimulates meiotic recombination in rice
Source: Front Plant Sci. 2025 May 1;16:1580225. doi: 10.3389/fpls.2025.1580225 (PMC12078263; doi:10.3389/fpls.2025.1580225)
Supplement: Supplementary file 11 [file DataSheet11.pdf]

Sample	Well	Nombre partitions valides	Nombre partitions positives	Nombre partitions négatives	KiZ1	KaZ4	KiZ3	KaZ2	KaZ4/KiZ1	KiZ3/KiZ1	KiZ3/KaZ4	KaZ2/KiZ1	KaZ2/KaZ4	KaZ2/KiZ3	KiZ3/KaZ4/KiZ1	KaZ2/KaZ4/KiZ1	KaZ2/KiZ3/KiZ1	KaZ2/KiZ3/KaZ4	KaZ2/KiZ3/KaZ4/KiZ1
A- Kitaake 2000	A1	25465	789	24676	21	0	32	32	0	106	0	39	0	117	0	0	442	0	0
B- Kalinga 2000	B1	25290	469	24821	0	67	0	126	0	0	2	0	274	0	0	0	0	0	0
C- 10001000	C1	25383	606	24777	25	47	67	88	0	251	0	0	128	0	0	0	0	0	0
D- 10001000	D1	25285	675	24610	43	41	64	76	0	269	0	0	182	0	0	0	0	0	0
E- 10001000	E1	25366	662	24704	31	51	72	68	0	266	0	1	173	0	0	0	0	0	0
F- 10001000	F1	25348	717	24631	55	41	60	88	0	245	0	1	227	0	0	0	0	0	0
G- 10001000	G1	25434	740	24694	32	67	117	78	0	242	1	0	202	0	1	0	0	0	0
H- 10001000	H1	25181	734	24447	40	63	58	85	1	275	0	0	209	1	1	1	0	0	0
H20	H3	25285	2	25283	0	0	0	2	0	0	0	0	0	0	0	0	0	0	0
I- 10001000	A2	25261	723	24538	47	59	66	71	1	284	1	1	193	0	0	0	0	0	0
J- Hybride 2000	B2	25446	638	24808	19	30	52	72	0	257	0	1	203	3	0	0	0	1	0
K- Hybride 2000	C2	25388	603	24785	18	31	37	53	0	248	0	0	212	1	0	2	0	1	0
L- Hybride 2000	D2	25442	616	24826	30	30	46	49	0	268	0	0	190	0	0	0	0	2	1
M- Hybride 2000	E2	25436	594	24842	33	34	37	60	0	224	0	1	200	1	2	1	1	0	0
N- Hybride 2000	F2	25428	692	24736	41	64	66	65	2	241	0	0	210	1	1	1	0	0	0
Q- T479 29.1 2000	A3	25318	378	24940	10	18	50	44	0	133	0	0	122	1	0	0	0	0	0
R- T479 29.1 2000	B3	25391	423	24968	23	17	15	47	0	171	0	0	150	0	0	0	0	0	0
S- T479 29.1 2000	C3	25382	475	24907	14	26	30	56	0	208	0	0	140	1	0	0	0	0	0
T- T479 29.1 2000	D3	25426	478	24948	17	29	45	36	0	177	0	0	172	0	1	1	0	0	0
U- T479 29.1 2000	E3	25445	518	24927	22	41	25	55	0	212	0	1	162	0	0	0	0	0	0
V- T479 29.1 2000	F3	25471	554	24917	18	36	47	49	0	212	0	0	189	1	0	1	0	1	0
W- T479 29.1 2000	G3	25375	584	24791	20	40	42	43	1	236	0	0	198	2	0	1	1	0	0

Sample	Well	Nombre partitions valides	Nombre partitions positives	Nombre partitions négatives	KiZ1	KaZ4	KiZ3	KaZ2	KaZ4/KiZ1	KiZ3/KiZ1	KiZ3/KaZ4	KaZ2/KiZ1	KaZ2/KaZ4	KaZ2/KiZ3	KiZ3/KaZ4/KiZ1	KaZ2/KaZ4/KiZ1	KaZ2/KiZ3/KiZ1	KaZ2/KiZ3/KaZ4	KaZ2/KiZ3/KaZ4/KiZ1
A- Kitaake 2000	A1	25456	363	25093	28	2	28	0	0	305	0	0	0	0	0	0	0	0	0
B- Kalinga 2000	B1	25345	525	24820	0	59	0	160	0	0	0	0	306	0	0	0	0	0	0
C- 10001000	C1	25414	469	24945	18	25	26	83	0	141	1	1	174	0	0	0	0	0	0
D- 10001000	D1	25366	488	24878	12	32	23	63	0	159	0	1	198	0	0	0	0	0	0
E- 10001000	E1	24510	423	24087	14	27	32	47	0	144	0	0	159	0	0	0	0	0	0
F- 10001000	F1	25225	422	24803	11	52	24	48	1	160	0	0	126	0	0	0	0	0	0
G- 10001000	G1	25454	555	24899	19	47	35	79	0	197	0	0	177	0	0	0	1	0	0
H- 10001000	H1	25381	571	24810	12	57	23	56	0	233	0	0	186	0	2	0	1	0	1
H20	H3	25368	4	25364	1	0	3	0	0	0	0	0	0	0	0	0	0	0	0
I- 10001000	A2	25333	546	24787	20	33	21	87	1	206	0	0	176	1	0	1	0	0	0
J- Hybride 2000	B2	25459	491	24968	22	45	46	56	0	175	2	0	143	1	1	0	0	0	0
K- Hybride 2000	C2	22168	330	21838	42	26	25	22	2	127	0	0	85	0	0	1	0	0	0
L- Hybride 2000	D2	25444	247	25197	9	26	54	15	1	74	0	0	67	0	1	0	0	0	0
M- Hybride 2000	E2	25476	346	25130	12	16	52	45	0	127	0	0	94	0	0	0	0	0	0
N- Hybride 2000	F2	23989	360	23629	14	22	38	28	1	151	0	0	102	2	1	0	0	1	0
O- Hybride 2000	G2	25268	414	24854	31	37	27	25	0	194	0	0	97	1	0	0	1	1	0
P- Hybride 2000	H2	21383	552	20831	24	45	27	43	0	245	1	0	160	1	3	1	1	1	0
Q- T479 29.1 2000	A3	25458	604	24854	11	32	26	42	0	268	0	0	217	3	5	0	0	0	0
R- T479 29.1 2000	B3	21414	413	21001	13	29	55	39	0	169	1	0	105	1	0	0	0	0	1
S- T479 29.1 2000	C3	25424	580	24844	15	23	28	63	1	258	0	0	187	1	2	1	0	0	1
T- T479 29.1 2000	D3	25498	608	24890	19	30	28	37	4	274	0	0	212	0	2	1	0	1	0
U- T479 29.1 2000	E3	25506	600	24906	15	42	31	57	2	251	0	1	199	0	2	0	0	0	0
V- T479 29.1 2000	F3	25414	584	24830	24	24	41	69	1	227	0	0	196	1	0	1	0	0	0
W- T479 29.1 2000	G3	25459	637	24822	22	45	40	58	0	247	0	0	223	1	0	1	0	0	0

Sample	Well	Nombre partitions valides	Nombre partitions positives	Nombre partitions négatives	KiZ1	KaZ4	KiZ3	KaZ2	KaZ4/KiZ1	KiZ3/KiZ1	KiZ3/KaZ4	KaZ2/KiZ1	KaZ2/KaZ4	KaZ2/KiZ3	KiZ3/KaZ4/KiZ1	KaZ2/KaZ4/KiZ1	KaZ2/KiZ3/KiZ1	KaZ2/KiZ3/KaZ4	KaZ2/KiZ3/KaZ4/KiZ1
A- Kitaake 2000	A1	25455	594	24861	63	0	80	0	0	451	0	0	0	0	0	0	0	0	0
B- Kalinga 2000	B1	25156	806	24350	0	73	0	151	0	0	0	0	582	0	0	0	0	0	0
C- 10001000	C1	25393	733	24660	36	42	48	92	1	213	0	2	298	0	0	1	0	0	0
D- 10001000	D1	25414	741	24673	45	40	40	69	1	235	0	0	311	0	0	0	0	0	0
E- 10001000	E1	25424	736	24688	36	54	60	80	1	219	0	0	285	0	0	0	0	1	0
F- 10001000	F1	25358	711	24647	43	43	59	81	3	183	0	0	296	0	0	2	1	0	0
G- 10001000	G1	25459	720	24739	41	53	57	92	0	225	1	0	248	0	3	0	0	0	0
H- 10001000	H1	25431	768	24663	49	69	80	95	0	199	0	0	275	0	0	1	0	0	0
H2O	H3	25408	1	25407	0	0	0	1	0	0	0	0	0	0	0	0	0	0	0
I- 10001000	A2	25299	569	24730	57	72	37	84	0	185	0	0	133	0	0	1	0	0	0
J- Hybride 2000	B2	25446	696	24750	27	56	101	75	0	241	0	0	192	1	3	0	0	0	0
K- Hybride 2000	C2	25396	753	24643	55	47	66	71	0	263	1	0	248	1	1	0	0	0	0
L- Hybride 2000	D2	25427	715	24712	44	60	89	66	0	261	0	1	193	1	0	0	0	0	0
M- Hybride 2000	E2	25483	663	24820	37	64	68	64	2	222	0	0	199	3	2	1	0	1	0
N- Hybride 2000	F2	25462	708	24754	56	78	57	62	2	223	1	2	224	0	0	0	3	0	0
O- Hybride 2000	G2	25301	771	24530	49	77	70	113	2	269	0	0	185	2	1	1	1	1	0
P- Hybride 2000	H2	25441	925	24516	56	104	79	86	1	312	0	0	283	1	2	1	0	0	0
Q- T479 29.1 2000	A3	24906	612	24294	10	63	58	43	4	244	3	0	185	1	1	0	0	0	0
R- T479 29.1 2000	B3	25430	568	24862	13	50	52	39	0	213	0	1	197	1	0	0	0	1	1
S- T479 29.1 2000	C3	25478	611	24867	24	50	57	77	2	216	0	1	179	2	2	1	0	0	0
T- T479 29.1 2000	D3	25446	688	24758	28	58	67	60	2	239	1	0	230	1	1	1	0	0	0
U- T479 29.1 2000	E3	25469	668	24801	41	60	61	74	0	218	0	0	211	1	0	1	0	1	0
V- T479 29.1 2000	F3	25438	644	24794	40	74	60	62	1	235	0	0	169	0	0	3	0	0	0
W- T479 29.1 2000	G3	25476	753	24723	43	129	92	70	0	226	0	1	191	1	0	0	0	0	0

Supplementary Table 3: dPCR results table for Chr.9 plants (1/2).

Plant 9a : T479 29.1.

Sample	Well	Nombre partitions valides	Nombre partitions positives	Nombre partitions négatives	KiZ1	KaZ4	KiZ3	KaZ2	KaZ4/KiZ1	KiZ3/KiZ1	KiZ3/KaZ4	KaZ2/KiZ1	KaZ2/KaZ4	KaZ2/KiZ3	KiZ3/KaZ4/KiZ1	KaZ2/KaZ4/KiZ1	KaZ2/KiZ3/KiZ1	KaZ2/KiZ3/KaZ4	KaZ2/KiZ3/KaZ4/KiZ1
A- Kitaake 2000	A1	25439	548	24891	88	0	113	0	0	347	0	0	0	0	0	0	0	0	0
B- Kalinga 2000	B1	25350	695	24655	0	102	0	127	0	0	0	0	466	0	0	0	0	0	0
H20	A3	25389	0	25389	0	0	0	0	0	0	0	0	0	0	0	0	0	0	0
J- Hybride 2000	C1	25429	803	24626	43	68	100	78	1	246	0	0	267	0	0	0	0	0	0
K- Hybride 2000	D1	25419	829	24590	33	43	56	71	1	330	0	0	293	1	1	0	0	0	0
L- Hybride 2000	E1	25430	857	24573	39	58	62	77	1	317	0	1	298	0	3	1	0	0	0
M- Hybride 2000	F1	25338	787	24551	29	63	34	75	3	335	0	0	247	1	0	0	0	0	0
N- Hybride 2000	G1	25516	799	24717	35	73	55	63	4	336	1	1	229	1	0	1	0	0	0
O- Hybride 2000	H1	25473	866	24607	43	57	70	86	1	331	1	2	270	1	2	1	1	0	0
P- Hybride 2000	A2	25379	800	24579	30	87	96	120	0	259	0	0	207	0	1	0	0	0	0
Q- T479 32.1 2000	B2	25446	715	24731	16	70	65	72	0	277	2	0	211	0	1	0	1	0	0
R- T479 32.1 2000	C2	25275	678	24597	21	22	31	77	1	323	0	1	200	1	0	0	0	0	1
S- T479 32.1 2000	D2	25440	602	24838	18	59	187	65	0	120	0	0	153	0	0	0	0	0	0
T- T479 32.1 2000	E2	25470	561	24909	21	32	34	51	2	229	1	0	191	0	0	0	0	0	0
U- T479 32.1 2000	F2	25414	596	24818	21	33	67	48	1	239	0	0	187	0	0	0	0	0	0
V- T479 32.1 2000	G2	25306	741	24565	30	55	81	54	1	277	0	0	241	0	0	2	0	0	0
W- T479 32.1 2000	H2	25447	802	24645	26	72	64	79	3	322	1	1	231	1	2	0	0	0	0

Sample	Well	Nombre partitions valides	Nombre partitions positives	Nombre partitions négatives	KiZ1	KaZ4	KiZ3	KaZ2	KaZ4/KiZ1	KiZ3/KiZ1	KiZ3/KaZ4	KaZ2/KiZ1	KaZ2/KaZ4	KaZ2/KiZ3	KiZ3/KaZ4/KiZ1	KaZ2/KaZ4/KiZ1	KaZ2/KiZ3/KiZ1	KaZ2/KiZ3/KaZ4	KaZ2/KiZ3/KaZ4/KiZ1
A- Kitaake 2000	A1	25439	473	24966	32	0	67	0	0	374	0	0	0	0	0	0	0	0	0
B- Kalinga 2000	B1	25341	487	24854	0	64	0	132	0	0	0	0	291	0	0	0	0	0	0
C- 10001000	C1	24716	429	24287	32	37	68	58	0	110	0	0	124	0	0	0	0	0	0
D- 10001000	D1	25431	524	24907	24	54	57	42	1	193	1	1	151	0	0	0	0	0	0
E- 10001000	E1	25360	577	24783	20	37	41	69	2	202	0	0	206	0	0	0	0	0	0
F- 10001000	F1	25103	531	24572	11	39	72	48	0	155	0	0	206	0	0	0	0	0	0
G- 10001000	G1	25229	583	24646	8	21	62	71	0	215	0	0	205	0	0	0	0	1	0
H- 10001000	H1	25405	580	24825	25	27	62	73	1	197	0	0	195	0	0	0	0	0	0
H20	H3	25424	0	25424	0	0	0	0	0	0	0	0	0	0	0	0	0	0	0
I- 10001000	A2	25395	568	24827	15	37	41	72	1	227	0	0	175	0	0	0	0	0	0
J- Hybride 2000	B2	25238	511	24727	52	27	41	50	0	166	0	0	175	0	0	0	0	0	0
K- Hybride 2000	C2	25305	538	24767	15	40	60	33	2	184	0	0	202	2	0	0	0	0	0
L- Hybride 2000	D2	25446	469	24977	29	25	46	44	1	182	0	0	140	2	0	0	0	0	0
M- Hybride 2000	E2	25480	391	25089	26	43	51	32	2	137	0	0	98	2	0	0	0	0	0
N- Hybride 2000	F2	25393	530	24863	14	40	39	35	0	259	0	0	141	0	0	0	1	1	0
O- Hybride 2000	G2	25026	439	24587	17	28	74	42	0	172	0	0	104	2	0	0	0	0	0
P- Hybride 2000	H2	25466	652	24814	30	43	62	52	1	259	0	0	199	4	0	1	0	1	0
Q- T479 32.1 2000	A3	25393	507	24886	18	27	26	45	3	211	0	0	176	1	0	0	0	0	0
R- T479 32.1 2000	B3	25459	505	24954	29	32	39	37	1	172	1	0	192	0	0	2	0	0	0
S- T479 32.1 2000	C3	25201	457	24744	17	45	54	38	0	180	0	0	121	2	0	0	0	0	0
T- T479 32.1 2000	D3	25376	353	25023	9	45	66	17	0	146	0	0	69	0	0	1	0	0	0
U- T479 32.1 2000	E3	25455	480	24975	20	44	53	35	1	209	0	0	116	0	0	1	0	0	1
V- T479 32.1 2000	F3	25434	532	24902	8	24	114	45	0	155	0	0	181	4	1	0	0	0	0
W- T479 32.1 2000	G3	25480	629	24851	23	58	49	56	1	212	1	0	228	1	0	0	0	0	0

Sample	Well	Nombre partitions valides	Nombre partitions positives	Nombre partitions négatives	KiZ1	KaZ4	KiZ3	KaZ2	KaZ4/KiZ1	KiZ3/KiZ1	KiZ3/KaZ4	KaZ2/KiZ1	KaZ2/KaZ4	KaZ2/KiZ3	KiZ3/KaZ4/KiZ1	KaZ2/KaZ4/KiZ1	KaZ2/KiZ3/KiZ1	KaZ2/KiZ3/KaZ4	KaZ2/KiZ3/KaZ4/KiZ1
A- Kitaake 2000	A1	25449	803	24646	34	0	96	0	0	673	0	0	0	0	0	0	0	0	0
B- Kalinga 2000	B1	25336	695	24641	0	60	0	185	0	0	0	0	450	0	0	0	0	0	0
C- 10001000	C1	25399	738	24661	23	50	70	64	3	296	0	0	232	0	0	0	0	0	0
D- 10001000	D1	24435	555	23880	25	41	76	52	2	189	0	0	170	0	0	0	0	0	0
E- 10001000	E1	25435	849	24586	24	29	69	79	1	335	0	1	311	0	0	0	0	0	0
F- 10001000	F1	25361	795	24566	19	45	39	74	1	358	0	0	255	0	3	1	0	0	0
G- 10001000	G1	25475	816	24659	21	39	50	83	0	347	0	1	273	0	0	2	0	0	0
H- 10001000	H1	25441	795	24646	20	65	44	91	2	333	0	0	240	0	0	0	0	0	0
H20	H3	25443	0	25443	0	0	0	0	0	0	0	0	0	0	0	0	0	0	0
I- 10001000	A2	25275	751	24524	28	59	73	55	0	272	0	0	263	0	0	1	0	0	0
J- Hybride 2000	B2	25469	612	24857	16	30	43	55	0	244	1	1	221	0	0	0	1	0	0
K- Hybride 2000	C2	25419	657	24762	23	30	50	68	1	245	0	0	239	0	0	1	0	0	0
M- Hybride 2000	E2	25463	504	24959	20	29	22	44	1	208	0	0	179	0	0	1	0	0	0
N- Hybride 2000	F2	25456	614	24842	21	54	61	40	3	234	2	0	194	2	2	0	0	0	1
O- Hybride 2000	G2	25030	744	24286	18	21	78	47	2	258	1	0	313	0	2	2	0	2	0
P- Hybride 2000	H2	25479	834	24645	30	44	84	70	0	301	0	0	302	2	0	1	0	0	0
Q- T479 32.1 2000	A3	25419	517	24902	26	45	38	33	0	213	0	0	161	0	0	0	0	1	0
R- T479 32.1 2000	B3	25466	569	24897	17	11	31	63	2	227	0	0	210	1	3	4	0	0	0
S- T479 32.1 2000	C3	25429	590	24839	28	20	54	53	1	224	0	0	207	0	1	1	1	0	0
T- T479 32.1 2000	D3	25476	629	24847	27	50	39	33	3	255	2	0	218	0	1	0	0	1	0
U- T479 32.1 2000	E3	25376	587	24789	19	32	50	46	1	221	1	1	216	0	0	0	0	0	0
V- T479 32.1 2000	F3	25439	617	24822	13	38	56	61	1	248	0	0	200	0	0	0	0	0	0
W- T479 32.1 2000	G3	25449	733	24716	18	35	42	70	1	320	0	1	242	1	0	1	1	0	1

Supplementary Table 3: dPCR results table for Chr.9 plants (2/2).
Plant 9b : T479 32.1.
