# Supplementary material for: dCas9-SPO11-1 locally stimulates meiotic recombination in rice
Source: Front Plant Sci. 2025 May 1;16:1580225. doi: 10.3389/fpls.2025.1580225 (PMC12078263; doi:10.3389/fpls.2025.1580225)
Supplement: Supplementary file 12 [file DataSheet12.pdf]

Supplementary Table 4: crRNA sequences.

	gRNA n°	crRNA sequence
Chr.7	1	TAAGCAGAAGCCATGTGCTA
	2	GTGCGTGTTTATAGCAAAT
	3	CGTAAGTAGTCGTTGATATG
	4	AGAGTCTATCTTCTTGGGTG
	5	TGACGACAGTGGAAAAGCGT
	6	GGGTTAAACCGACATCGACC
	7	CATTTGTTCTCTTGGTCTAT
	8	AGCGGGGAGGACATTACACA
	9	TGATGATGGACTCCTAGCAA
	10	GGATTCAAATACCCTTCACA
	11	TTTGGGTTTAATCCTCCGAT
Chr.9	1	TTCTGATTCTTGTCAACTAG
	2	AAGTGAGTATACAGCTAAAA
	3	GGATACACTCAGATTCTAGA
	4	TTGCTGCACCGGCCACAACG
	5	TGAGAGCTAAAAAGAGGCCC
	6	ACCACTCCCCCCTGTGATGA
	7	GCCTAACGACGGTCAGCTCG
	8	GTGATCTTCTTCGTTACTGA
	9	AAATTTGGGGTTGTATCAGA
	10	GATAGGGAACTTCGGTTAAT
	11	CTAGCTTGATTTGTCTATCG
