# Supplementary material for: dCas9-SPO11-1 locally stimulates meiotic recombination in rice
Source: Front Plant Sci. 2025 May 1;16:1580225. doi: 10.3389/fpls.2025.1580225 (PMC12078263; doi:10.3389/fpls.2025.1580225)
Supplement: Supplementary file 13 [file DataSheet13.pdf]

	name	sequence	Size
**Cas9 insertion**	OsCas9-F2	TGCCTGCGGAGGATAGCATGAAGCTC	896 bp
	OsCas9-R2	TACCACGAGAAGTACCCGACCATCT	
**SPO11-1**	SPO11-1-F1	CCAAAATTCTTGTGGGTGCT	800 bp
	SPO11-1-R1	CGGAGGAGCAGTAGTTCTGG	
**NPTII insertion**	NPT-II-F	GCGATAGAAGGCGATGCG	393 bp
	NPT-II-R	CCGGCTACCTGCCCATTCGA	
**tRNA:gRNA insertion**	OsU3 F2 - Common	CTGGGTACGTTGGAAACCAC	2041 bp
	gRNA-7-11-R	ATCGGAGGATTAAACCCAAA	
	gRNA-9-11-R	CGATAGACAAATCAAGCTAGTGC	
**gRNA cDNA**	qsg(*choose*)-F1	Supplemental Table 4	92 bp
	qSgRNA	CGACTCGGTGCCACTTTTTCAAGTTG	
**Chr.7 Region**	Chr.7-F2	TGCAAGGAATCCCCCATTCA	1895 to 1920 bp
	Chr.7-R2	GGGAAGACCGAAGAAATGGGAT	
**Chr.7 Recombinant Plant Region**	Chr.7-O20-F1	TGGGGTTTCTTCTAGAGCCG	974 to 976 bp
	Chr.7-O20-R1	TGTGGACTTGGTCCATGCAG	
	Chr.7-O20-F2	TGCTCCAAACGGGTCCATAG	431 bp
	Chr.7-O20-R2	ATGCTTCACAAGGCAAAGACAC	
	Chr.7-O20-F3	GGGCCTTAGTACTTTCCAGTGA	796 bp
	Chr.7-O20-R3	TGTCCTTTGATCCTTTCACACT	
	Chr.7-O20-F4	AAATGGCCTTCCACTGCCTT	802 to 791 bp
	Chr.7-O20-R4	AGATTCCGATTGGCACAACTT	

Supplementary Table 5: PCR primers.
Primers used for plant genotyping and description.
