# Supplementary material for: dCas9-SPO11-1 locally stimulates meiotic recombination in rice
Source: Front Plant Sci. 2025 May 1;16:1580225. doi: 10.3389/fpls.2025.1580225 (PMC12078263; doi:10.3389/fpls.2025.1580225)
Supplement: Supplementary file 14 [file DataSheet14.pdf]

| | Name | Sequence | Size |
|---|---|---|---|
| **qPCR33 (Actin 1, Os03g50885.1)** | qPCR33 F | GCGTGGACAAAGTTTTCAACCG | 153 pb |
| | qPCR33 R | TCTGGTACCCTCATCAGGCATC | |
| **Hpt** | qHPT-F | ctatttctttgccctcggacga | 60 pb |
| | qHPT-R | ggaccgatggctgtgtagaag | |
| **NPTII** | qNPT-F | aggaagcggtcagcccat | 60 pb |
| | qNPT-R | gcgttggctacccgtgatat | |
| **EXP' (Kitaake Os07g010600.1)** | EXP' F | AGGAACATGGAGAAGAACAAGG | 112 pb |
| | EXP' R | CAGAGGTGGTGCAGATGAAA | |
| **Fusion** | qfd9SPO11-F3 | cacaactcggcggcgatC | 70 pb |
| | qfd9SPO11-R3 | ttctccctccccgccatAC | |
| **OsSPO11-1** | qPCR_OsSPO11-1 _F | GATGCAAATTTCCTGCGTGT | 107 pb |
| | qPCR_OsSPO11-1 _R | TCAGACGACAAGTGAAGTAGGC | |
| **dCAS9** | qdCAS9-F1 | atgccaacctcgataaggtg | 95 pb |
| | qdCAS9-R1 | gagggtgaagaggtggatga | |
| **sgRNA** | qSgRNA - Common R | CGACTCGGTGCCACTTTTTCAAGTTG | 92 pb |
| | q7sg1-F1 | TAAGCAGaagcCATGTGCTA | |
| | q7sg2-F1 | GTGCGTGTTtataGCAAATG | |
| | q7sg3-F1 | CGTAAGTagtcGTTGATATG | |
| | q7sg6-F1 | GGGTTAAaccgACATCGACC | |
| | q7sg11-F1 | TTTGGGTTTAATCCTCCGAT | |
| | q9sg4-F1 | TTGCTGCACCGGCCACAACG | |
| | q9sg5-F1 | TGAGAGCTAAAAAGAGGCCC | |
| | q9sg8-F1 | GTGATCTTCTTCGTTACTGA | |
| | q9sg12-F1 | CTAGCTTGATTTGTCTATCG | |

**Supporting Table 6: qPCR primers.**
