# Supplementary material for: dCas9-SPO11-1 locally stimulates meiotic recombination in rice
Source: Front Plant Sci. 2025 May 1;16:1580225. doi: 10.3389/fpls.2025.1580225 (PMC12078263; doi:10.3389/fpls.2025.1580225)
Supplement: Supplementary file 15 [file DataSheet15.pdf]

Name	Target	Position	Sequence primer 1	Sequence primer 2	Sequence common primer
Ch9_Z1	Chr.9	11 927 000	GAAGGTGACCAAGTTCATGCTAAATGATATACTTTGACAAGTTCTGAGACA	GAAGGTCGGAGTCAACGGATTGATATACTTTGACAAGTTCTGAGACC	GGTTGTGTCATATAGATCCCTAAACTCTT
Ch9_Z2	Chr.9	11 966 000	GAAGGTGACCAAGTTCATGCTAGAGTTGAGTGGTGGGAGGAGA	GAAGGTCGGAGTCAACGGATTAGAGTTGAGTGGTGGGAGGAGT	GCCTRCAAAAATCGTACCAACCCAAAA
Ch9_Z3	Chr.9	11 981 000	GAAGGTGACCAAGTTCATGCTATGCAAATTTAAATAGAGATACTAAATGCCTTT	GAAGGTCGGAGTCAACGGATTATGCAAATTTAAATAGAGATACTAAATGCCTTA	GTCAACAAGCTGACTTAAAAGTGTAAGCTA
Ch9_Z4	Chr.9	11 994 000	GAAGGTGACCAAGTTCATGCTCCTGCAAAATAAGTCAACCAAAACAAAAC	GAAGGTCGGAGTCAACGGATTACCTGCAAAATAAGTCAACCAAAACAAAAA	GAATAAGACGAATGGTCAAACACGTACTA
Ch7_Z1	Chr.7	16 995 400	GAAGGTGACCAAGTTCATGCTGTAGCTAGCAAGCTAATTAAACGTAGTA	GAAGGTCGGAGTCAACGGATTGTAGCTAGCAAGCTAATTAAACGTAGTT	GAGAGTGTGAGGTCAGYTATACCCAT
Ch7_Z2	Chr.7	17 002 250	GAAGGTGACCAAGTTCATGCTCATATATGTAATGTAATAGTAGTCCTGAAGT	GAAGGTCGGAGTCAACGGATTATATATGTAATGTAATAGTAGTCCTGAAGG	GGCAGAGCTGSCTTTATTTAAAAGGAAAA
Ch7_Z3	Chr.7	17 009 480	GAAGGTGACCAAGTTCATGCTATGTGTCCCGCTCTGTCGCTA	GAAGGTCGGAGTCAACGGATTGTGTCCCGCTCTGTCGCTG	AAAAAGGAAGAAAAATAGAGCGAGACAGAA
Ch7_Z4	Chr.7	17 017 550	GAAGGTGACCAAGTTCATGCTGTGCATGGATAACCTAATAGGATAATCT	GAAGGTCGGAGTCAACGGATTGCATGGATAACCTAATAGGATAATCC	CTCTACAKGATGCAATGCTCTGGTTTT
Ch7_ZA	Chr.7	13 356 485	GAAACTCACTTTTGTTGCTAAGTTAAACCACAGAGAACACACACACTTGACACTTCTACAGAAAAAAAATGGATTTTGTGCCTGAATTTCGTAGTAGAGA[G/A]CATACACAAATTCATAAACTCAGTCTAATTTGGAATGAAAACTTGAGTTTGTAATATTCAAATGTACATCAAATCAGGTTGTTCTGGTAAATCGGCTTAG		
Ch7_ZB	Chr.7	13 374 324	GCCCACGGGCACGCGTCGCTCCCGCGGTCTTCCTCCCGTTGCTTCCTCATATCCGCCGATAAAACGGATAAGCCCGGCCACCCACCAAACCCTGCACTTT[T/A]AAATCCGTCGTCCTTCTAGCTAGTGTTGCCCACCTTGATTTGCTCGTACTTTGATTCTTTCTGCCTCTGCGTACGTGCGTGAGAGAGCTAAGCATAGAGC		
Ch7_ZC	Chr.7	18 116 930	GACAGTCACTGTGAAATGCTACTGTT	ACAGTCACTGTGAAATGCTACTGTC	GAAGTGATGGCTATGATATCATGGTGATT
Ch7_ZD	Chr.7	21 193 177	GAACTCGTACCCACGTTTAGGCT	AACTCGTACCCACGTTTAGGCC	CCAATAGACGCGCTGATTTTCTAGTTTTA

Supplementary Table 7: Kasp primers.

Target	Primer Name	Primer sequence	Size (pb)	Kitaake Position	KalingaIII Position
**EXP (Kitaake *Os06g078500.1*)**	OsEXPchip_F	CGGTTAGCTAGAGTTCATGTGA	217	Chr.6 : 5971123 to 5971339	Chr.6 : 6117682 to 6117898
	OsEXPchip_R	ATTGGAGTAGTGGAGTGCCAAA			
**Chr.7 area 1**	qPCR_ch7_F1	TGGTGATGATGGTGATGAGC	100	Chr.7 : 17005733 to 17005832	Chr.7 : 18629138 to 18629231
	qPCR_ch7_R1	AAGCCATGTGCTAGGGAAAG			
**Ch7 area 2**	qPCR_ch7_F3	TCCCATTTCTTCGGTCTTCC	100	Chr.7 : 17007266 to 17007365	Chr.7 : 18630696 to 18630795
	qPCR_ch7_R3	GTGTCGCTTTCCAAATTGC			
**Chr.9 area 1**	qPCR_ch9_F12	CATGGATCTGTACATCCGAGTAG	113	Chr.9 : 11974872 to 11974984	Chr.9 : 13102329 to 13102441
	qPCR_ch9_R12	CGTGTTCGTTCTTGTGCTTG			
**Chr. 9 area 2**	qPCR_ch9_F18	TTGATTGCACCCCTAGATGG	105	Chr.9 : 11976250 to 11976354	Chr.9 : 13103714 to 13103818
	qPCR_ch9_R18	TCAAATCTAGCCCCCACTTG			

Supplementary Table 8: ChIP qPCR primers.

Chromosome	Probe names	Sequence primer F	Sequence primer R	Sequence Probe
Chr.7	1 - Kitaake: 7#1	GAGAGGCATGTTATTTGACG	ATCCGAAATGAGTCGTAGC	AGTTATACCCATGACTACTACGT
	2 - KalingaIII: 7#2	TGGCAGAGCTGGCTTTAT	CTGGTGTGGGACTGTTGT	TACATGTTGGTTCTATTCCTTCAGGACTAC
	3 - Kitaake: 7#3	ACGAAAAGCATTAAAAGGGC	TATGTTGCACAAAGGCTCC	ACAGAACAGAGAAAGTAGCGACAGAGC
	4 – KalingaIII: 7#4	CAGCCTCTGCCTCTACA	ATTCTATGGATAAATGTGCATGG	TTTTCTTCTGCCTGGATTATCCTATTAGGT
Chr.9	1 - Kitaake: 9#1	AGGGTATTGAAGGGATTGAGT	TCCATACATTAGTTTCACCAGAA	CAGGGGCATATAGGGAATTTTCTCTTAGTT
	2 - KalingaIII: 9#2	GTAGCCATGAAGCTTAACCC	ATGTGTCACAAGGCTAATCC	ATGCTAAAACAATGGGAATGGGAAGTACAT
	3 - Kitaake: 9#3	TCCATACATTAGTTTCACCAGAA	TCCTCATTCCCAAACATCAC	AGTTATTGATACTTAAAGGAAAATAAATTG
	4 - KalingaIII: 9#4	TATAGTACTCCCTCCCTCTGT	GAATAAGACGAATGGTCAAACAC	TACAATGTTTGACACTGTTAACTTTTTAGT

Supplementary Table 9: dPCR primers and probes.

MSU	RAP	Kitaake
LOC_Os03g50885.1	Os03g0718100	OsKitaake03g316400
LOC_Os03g54091.1	Os03g0752200	OsKitaake03g341100.1
LOC_Os06g11070.1	Os06g0214100	OsKitaake06g078500.1
LOC_Os07g02340.1	Os07g0114300	OsKitaake07g010600.1
LOC_Os07g29290.1	Os07g0475400	OsKitaake07g138900.1
nc	nc	OsKitaake07g138950.1
LOC_Os09g20420.1	Os09g0370000	OsKitaake09g076100.1
LOC_Os09g20430.1	Os09g0370200	OsKitaake09g076200.2
LOC_Os09g20440.1	Os09g0370300	OsKitaake09g076300.1
LOC_Os09g20460.1	Os09g0370500	OsKitaake09g076400.1

Supporting Table 10: Gene names and correspondences.
